# Performance analysis of markers for prostate cell typing in single-cell data

**DOI:** 10.1016/j.gendis.2023.101157

**Published:** 2023-10-26

**Authors:** Yanting Shen, Xiawei Fei, Junyan Xu, Rui Yang, Qinyu Ge, Zhong Wang

**Affiliations:** a Department of Urology, Shanghai Ninth People's Hospital, Shanghai Jiaotong University School of Medicine, Shanghai 200011, China; b Department of Urology and Andrology, Gongli Hospital, The Second Military Medical University, Shanghai 200135, China; c State Key Laboratory of Bioelectronics, School of Biological Science and Medical Engineering, Southeast University, Nanjing, Jiangsu 210096, China; d Wuxi Maternal and Child Health Hospital, Wuxi School of Medicine, Jiangnan University, Jiangsu 214002, China; e Department of Urology, Qingpu Branch of Zhongshan Hospital Affiliated to Fudan University, Shanghai 201799, China; f University of Shanghai for Science and Technology, Shanghai 200093, China

Cell typing is an important step in the single-cell RNA sequencing (scRNA-seq) analysis. Although some cell marker databases and cell typing tools have been proposed, limited roles are in prostate cell typing. Through literature review, we found prostate cell typing relied much on researchers' knowledge and experience, thus different markers were used to label the same cell type, leading to the divergences between studies, emphasizing the importance of a sound epistemological foundation for prostate cell typing in single-cell data. Therefore, we designed this study to provide performance analysis for prostate cell markers using eight integrated human prostate scRNA-seq datasets of 170,438 cells from 41 peoples (methods were described in Supplementary Data 2 in detail). Using unsupervised learning, information entropy, F1-score, and local outlier factor score, an objective performance analysis report was obtained, based on which, stable and specific human prostate main and fine cell markers were proposed. Our findings will help decide to select suitable markers for human prostate cell typing in single-cell data.

An extensive literature review of human prostate scRNA-seq studies was conducted to summarize prostate cell types and markers. As expected, all the included studies labeled the prostate main cell types (epithelial and stromal cells), but divergences were found in their fine cell types (Table S1). Among epithelial fine cell types, luminal and basal cells were labeled in about 50% of the included studies, while club, hillock, and neuroendocrine cells were only labeled in about 15%–20% of the included studies. Among stromal fine cell types, endothelial cells, fibroblasts, and smooth muscle cells were labeled in more than 50% of the included studies, whereas myofibroblasts and mesenchymal cells were only labeled in less than 25% of the included studies. In addition, after stromal sub-clustering, pericytes were labeled in about 15% of the included studies, suggesting their constituent for stromal cells in human prostate tissues. We then summarized markers of these cell types (Table S2), and evaluated their stability and specificity using eight integrated human prostate scRNA-seq datasets, which were different in sample types, sampling positions, and cell proportion (Table S3 and Fig. S1). Mesenchymal cells were excluded because the markers were not provided in the included studies.

Given the importance of accurate main cell typing for the subsequent fine cell typing, we evaluated the performance of human prostate main cell markers. Firstly, we used Uniform Manifold Approximation and Projection (UMAP) to visualize the similarity between markers of the same cell type and the heterogeneity between markers of the different cell types,^1^^,^^2^ assuming that ideal markers of each cell type should be clustered relatively independently in UMAPs of all the integrated datasets. As shown in Figure S2, we found the abnormal discrete distribution of some epithelial (*AR*, *TEAD1*, *IER3*, *EGR1*, *DST*, S100A6, *ID1*, *SERPINB1*, *PLA2G2A*, *CHGB*, *RARRES1*, *EZH2*, and *SIAH2*) and stromal cell markers (*C1S* and *FBLN1*), suggesting their poor cell typing abilities. Then, we used the entropy evaluation method to calculate the information entropy of avg_log2FC, pct.1, diff_pct, and *p*_value_adj for each marker. These four values, gained by differential expression gene analysis, are widely considered important for determining whether a gene can be treated as the characteristic gene to assign a cluster to a certain cell type. In previous studies, researchers usually set thresholds for them subjectively to select characteristic genes, which made cell typing between studies divergent. However, the entropy evaluation method can largely avoid this defect.^3^ After differential expression gene analysis, we screened eight epithelial markers (*KRT8*, *KRT18*, *KRT15*, *KRT17*, *KRT19*, *KRT7*, *AGR2*, and *CLDN4*) that were significantly up-regulated in epithelial cell clusters and 26 stromal markers (*CLDN5*, *SELE*, *VWF*, *ENG*, *IGFBP7*, *IFI27*, *EMCN*, *CD200*, *C7*, *VIM*, *PTGDS*, *GJA4*, *RGS5*, *MT1A*, *COL1A2*, *MYH11*, *ACTG2*, *BGN*, *THY1*, *PDGFRB*, *NRP1*, *ANGPT2*, *COL3A1*, *COL4A1*, *COL4A2*, and *COL18A1*) that were significantly up-regulated in stromal cell clusters (Fig. S3). Pearson correlation analysis showed that the information entropy between normal tissues had a high similarity, as did benign prostatic hyperplasia (BPH) tissues and tumor tissues (Fig. 1A), suggesting the cell typing abilities of these 34 screened markers were mainly affected by disease rather than sampling location and cell proportion. We also calculated the total information entropy and rank sum and proposed that for all types of human prostate tissues, *KRT18*, *KRT8*, and *CLDN4* were the top three robust epithelial cell typing markers, as well as *IGFBP7*, *VIM*, and *IFI27* were the top three robust stromal cell typing markers (Fig. 1B and Table S4). Finally, we used K-Means clustering^4^ and F1-score for validation. According to the average expression levels of the eight epithelial markers, the non-epithelial Seurat clusters were clustered and distributed away from the epithelial Seurat clusters in almost all the integrated datasets (Fig. 1C). Similarly, according to the average expression levels of the 26 stromal makers, the stromal Seurat clusters were also obviously distributed far away the non-stromal Seurat clusters in all the integrated datasets (Fig. 1D). Furthermore, the F1-scores of the 34 screened markers were all more than 0.8 (Table S5–7 and Fig. S4, 5). These findings showed that the 34 screened markers were stable and specific for prostate main cell typing.

**Figure 1:**
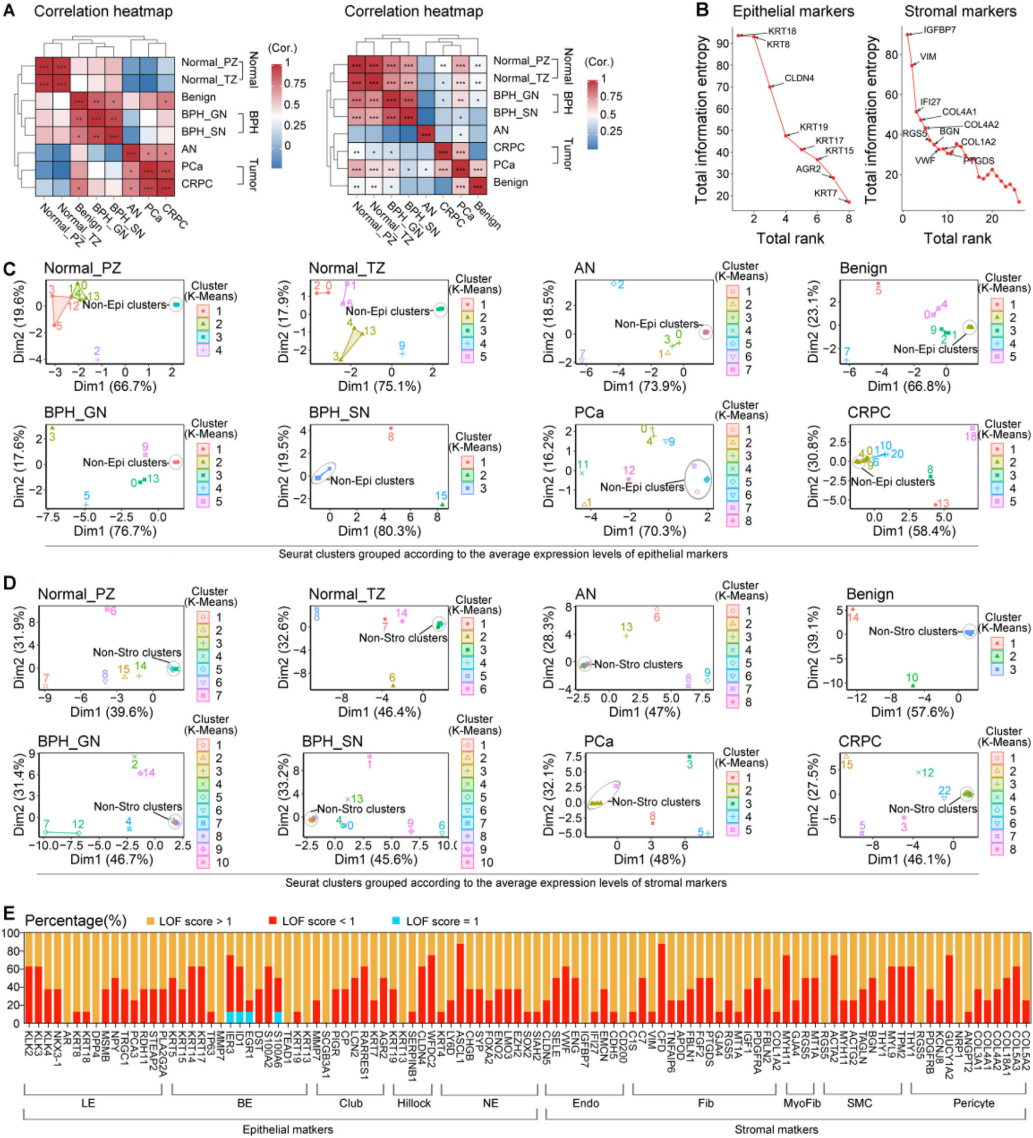
Performance analysis of the human prostate cell markers. **(A)** Pearson correlation heatmaps of the information entropy of the eight epithelial markers (left) and 26 stromal markers (right). **(B)** Total rank and information entropy of the eight epithelial (left) and 26 stromal (right) markers across all the integrated human prostate scRNA-seq datasets. **(C, D)** Regrouping the Seurat clusters of the eight integrated human prostate scRNA-seq datasets according to the average expression levels of the eight epithelial markers **(C)** and 26 stromal markers **(D)**. **(E)** Percentage of the integrated datasets with local outlier factor (LOF) score <1, LOF score = 1, and LOF score >1 for each fine cell marker. Eight integrated human prostate scRNA-seq datasets including Normal_PZ, Normal_TZ, BPH_GN, BPH_SN, AN, Benign, PCa, and CRPC were described in detail in Table S3. Non-Epi clusters: non-epithelial clusters included stromal clusters and immune clusters; Non-Stro clusters: non-stromal clusters included epithelial clusters and immune clusters.

For human prostate fine cell marker evaluation, UMAPs were also performed, and showed obvious differences in the dispersion degree of the markers for the same cell type across the eight integrated datasets (Fig. S6), indicating the relative poor fine cell typing stability across different prostate sample types. Given the co-expression of the fine cell markers, we then calculated local outlier factor scores^5^ to strictly and quantitatively identify the markers with abnormal discrete distribution in UMAPs. As shown in Figure 1E, luminal cell markers *AR* and *DPP4*, basal cell makers *MMP7*, *TEAD1*, and *KRT13*, club cell marker *SCGB3A1*, hillock cell marker *KRT13*, endothelial cell markers *IGFBP7* and *CD200*, smooth muscle cell marker *RGS5*, and pericyte markers *RGS5* and *NRP1* had local outlier factor scores greater than one in all the integrated datasets. They were considered to be with unstable fine-cell typing abilities. Thus, they were removed from the list of candidate markers, and a new stable marker set of prostate fine cell types was constructed (Table S8). Finally, for validation, we calculated F1-scores of luminal cell, endothelial cell, basal cell, and fibroblast markers in more than 60% of integrated datasets (Table S9), and found in 80% of them, F1-scores of luminal cell markers *KLK2*, *KLK3*, *KLK4*, *NKX3-1*, and *STEAP2*, basal cell markers *KRT15*, *KRT17*, and *KRT19*, fibroblast markers *APOD*, *FBLN1*, *FGF2*, *PDGFRA*, and *FBLN2*, and endothelial cell markers *CLDN5*, *SELE*, *VWF*, *ENG*, *IFI27*, *EMCN*, and *CDH5* were more than 0.6, indicating their relatively high specific and stable performance for prostate fine cell typing. Besides, F1-scores of smooth muscle cell markers were calculated in one integrated dataset, and *MYH11* and *ACTG2* showed relatively high specificity for smooth muscle cell typing with F1-scores more than 0.6. Club cell, hillock cell, neuroendocrine cell, myofibroblast, and pericyte markers were not subjected to validation due to their poor stability and specificity. Nevertheless, we provide them with an objective preliminary evaluation (Fig. 1E), which can help researchers make decisions when they do the cell typing.

In summary, we provided an objective performance analysis report of human prostate cell markers in this study, based on which, stable and specific human prostate main and fine cell markers were proposed. Our findings will benefit cell typing of human prostate scRNA-seq studies.

## Author contributions

Zhong Wang, Qinyu Ge, and Rui Yang had full access to the data in the study and took responsibility for the integrity of the data, the accuracy of the data analysis, and the critical revision of the manuscript for important intellectual content. Yanting Shen and Xiawei Fei took the main responsibility for the concept, design, data analysis, and paper written. Junyan Xu assisted in data processing and manuscript revising.

## Conflict of interests

The authors declare no competing financial interest.

## Funding

This study was supported by the 10.13039/501100001809National Natural Science Foundation of China, China (No. 62101319, 82170788), and the Medical Discipline Construction Project of the Health System of Pudong New Area (China) (No. PYWgf2021-06).
