# Supplementary material for: Performance analysis of markers for prostate cell typing in single-cell data
Source: Genes Dis. 2023 Oct 26;11(6):101157. doi: 10.1016/j.gendis.2023.101157 (PMC11295451; doi:10.1016/j.gendis.2023.101157)
Supplement: Multimedia component 1 [file mmc1.docx]

**Supplementary Data 1**

Supplementary Tables and Figures

| Supplementary Tables | **Numbered pages** |
| --- | --- |
| **Supplementary Table S1.** Human prostate cell types | 2 |
| **Supplementary Table S2.** Cell markers commonly used in the included human prostate scRNA-seq studies | 3 |
| **Supplementary Table S3.** Information of the eight integrated human prostate scRNA-seq datasets | 4 |
| **Supplementary Table S4.** Epithelial markers ranked by their information entropy | 6 |
| **Supplementary Table S5.** Validating the performance of the 34 epithelial and stromal cell markers | 7 |
| **Supplementary Table S6.** DEGA for the KMeans-clusters reclustered according to the eight epithelial markers | 8 |
| **Supplementary Table S7.** DEGA for the KMeans-clusters reclustered according to the 26 stromal marker genes | 17 |
| **Supplementary Table S8.** Reconstructed prostate fine cell marker gene sets | 30 |
| **Supplementary Table S9.** F1-scores of marker genes for fine cell typing | 31 |
| Supplementary Figures | **Numbered pages** |
| **Supplementary Figure S1.** Main cell proportion of the eight integrated human prostate scRNA-seq datasets | 33 |
| **Supplementary Figure S2.** UMAPs for epithelial and stromal markers of the eight integrated human prostate scRNA-seq datasets | 34 |
| **Supplementary Figure S3.** Venn plot of the significantly upregulated epithelial and stromal cell markers | 35 |
| **Supplementary Figure S4.** DotPlots of the 34 main cell markers in the KMeans-clusters reclustered according to the average expression levels of the eight epithelial markers | 36 |
| **Supplementary Figure S5.** DotPlots of the 34 main cell markers in the KMeans-clusters reclustered according to the average expression levels of the 26 stromal markers | 37 |
| **Supplementary Figure S6.** Evaluating the performance of human prostate fine cell markers using UMAP approach. | 38 |

Supplementary Tables

**Supplementary Table S1.** Human prostate cell types

| **Cell type (main)** | **Cell type (fine)** | **Human prostate scRNA-seq studies** |
| --- | --- | --- |
| Epithelial cell | ~ | Dong B, et al. (2020); Ge G, et al. (2022); Yan Q, et al. (2022); Joseph DB, et al. (2021); Wong HY, et al. (2022); Heidegger I, et al. (2022) |
| Epithelial cell | LE cell | Song H, et al. (2022); Chen S, et al. (2021); Ma X, et al. (2020); Tuong ZK, et al. (2021); Henry GH, et al. (2018); Chen Y, et al. (2021); Crowley L, et al. (2020) |
| Epithelial cell | BE, BE/Intermediate cell | Song H, et al. (2022); Chen S, et al. (2021); Ma X, et al. (2020); Tuong ZK, et al. (2021); Henry GH, et al. (2018); Chen Y, et al. (2021); Crowley L, et al. (2020) |
| Epithelial cell | Club cell | Song H, et al. (2022); Tuong ZK, et al. (2021); Henry GH, et al. (2018) |
| Epithelial cell | Hillock cell | Song H, et al. (2022); Tuong ZK, et al. (2021); Henry GH, et al. (2018) |
| Epithelial cell | NE cell | Ma X, et al. (2020); Henry GH, et al. (2018) |
| Stromal cell | Endo cell | Song H, et al. (2022); Chen S, et al. (2021); Ma X, et al. (2020); Tuong ZK, et al. (2021); Henry GH, et al. (2018); Chen Y, et al. (2021); Crowley L, et al. (2020); Dong B, et al. (2020); Ge G, et al. (2022); Yan Q, et al. (2022); Joseph DB, et al. (2021); Wong HY, et al. (2022); Heidegger I, et al. (2022) |
| Stromal cell | Fib | Song H, et al. (2022); Chen S, et al. (2021); Ma X, et al. (2020); Tuong ZK, et al. (2021); Henry GH, et al. (2018); Chen Y, et al. (2021); Dong B, et al. (2020); Ge G, et al. (2022); Yan Q, et al. (2022); Joseph DB, et al. (2021); Wong HY, et al. (2022); Heidegger I, et al. (2022) |
| Stromal cell | SMC | Song H, et al. (2022); Ma X, et al. (2020); Henry GH, et al. (2018); Chen Y, et al. (2021); Crowley L, et al. (2020); Joseph DB, et al. (2021); Wong HY, et al. (2022) |
| Stromal cell | MyoFib | Ma X, et al. (2020); Dong B, et al. (2020); Yan Q, et al. (2022) |
| Stromal cell | Mesenchymal cell | Ma X, et al. (2020) |
| Stromal cell | ^1^Pericyte | Joseph DB, et al. (2021); Heidegger I, et al. (2022) |

^1^ After stromal sub-clustering, two human prostate scRNA-seq studies labeled pericytes.

**Supplementary Table S2.** Cell markers commonly used in the included human prostate scRNA-seq studies

| **Cell type (main)** | **Cell type (fine)** | **Cell Markers** |
| --- | --- | --- |
| Epithelial cell | LE cell | *KLK2, KLK3, KLK4, ACPP, NKX3-1, AR, KRT8, KRT18, DPP4, MSMB, NPY, TRGC1, PCA3, RDH11, STEAP2, PLA2G2A* |
|  | BE cell | *KRT5, KRT15, KRT14, KRT17, TP63, KRT19, MMP7, IER3, ID1, EGR1, KRT13, DST, S100A2, S100A6, TEAD1, TAZ, HMWCK* |
|  | Club cell | *SCGB3A1, PIGR, MMP7, CP, LCN2, RARRES1, KRT7, AGR2* |
|  | Hillock cell | *KRT13, SERPINB1, CLDN4, APOBEC2, KRT19, WFDC2* |
|  | NE cell | *KRT4, LY6D, ASCL1, CHGA, CHGB, SYP, FOXA2, NKX2-1, ENO2, MYCN, POU3F2, LMO3, INSM1, EZH2, SOX2, SIAH2, PLP1, MPZ* |
| Stromal cell | Endo cell | *CLDN5, SELE, PECAM1, VWF, ENG, IGFBP7, ACKR1, IFI27, EMCN, CDH5, CD200* |
|  | Fib | *C1S, DCN, C7, VIM, LUM, CFD, TNFAIP6, APOD, FBLN1, FGF2, PTGDS, RSPO3, GJA4, RGS5, MT1A, IGF1, PDGFRA,* *FBLN2, COL1A2* |
|  | SMC | *ACTA2, MYH11, RGS5, ACTG2, DES, TAGLN, BGN, THY1, MYL9, TPM2* |
|  | MyoFib | *MYH11, GJA4, RGS5, MT1A* |
|  | Pericyte | *THY1, RGS5, PDGFRB, KCNJ8, GUCY1A2, GUCY1A1, GUCY1B1, NRP1, ANGPT2, COL3A1, COL4A1, COL4A2, COL18A1, COL5A3, COL5A2* |

**Supplementary Table S3.** Information of the eight integrated human prostate scRNA-seq datasets

| **Integrated scRNA-seq datasets** | **Description** | **scRNA-seq matrices** | **Number of samples** | **Data filtering criteria** | **Number of cells after filtering** |
| --- | --- | --- | --- | --- | --- |
| Normal_PZ | Normal prostate tissue of peripheral zone (PZ) taken from the young healthy donors | GSM5252457, GSM5252459, GSM5252461 | 3 | nFeature_RNA>200&nFeature_RNA<4000&nCount_RNA<20000 | 20,821 |
| Normal_TZ | Normal prostate tissue of transition zone (TZ) taken from the young healthy donors | GSM5252458, GSM5252460, GSM5252462 | 3 | nFeature_RNA>200&nFeature_RNA<4000&nCount_RNA<20000 | 18,930 |
| AN | Adjacent normal (AN) prostate tissue taken from the PCa patients | GSM5353230, GSM5353231, GSM5353234, GSM5353235, GSM5353238, GSM5353239, GSM5353241, GSM5353242, | 4 | nFeature_RNA>200&nFeature_RNA<2000&nCount_RNA<5000 | 3,479 |
| Benign | Benign prostate tissue taken from the bladder cancer patients | GSM4556600, GSM4556601, GSM4556602 | 3 | nFeature_RNA>200&nFeature_RNA<4000&nCount_RNA<20000 | 5,174 |
| BPH_GN | Prostate gland nodule (GN) tissue taken from the benign prostatic hyperplasia (BPH) patients without 5AIR treatment | GSM5252126, GSM5252128, GSM5252130, GSM5252132, GSM5252134 | 5 | nFeature_RNA>200&nFeature_RNA<4000&nCount_RNA<15000 | 32,275 |
| BPH_SN | Prostate stromal nodule (SN) tissue taken from the benign prostatic hyperplasia (BPH) patients without 5AIR treatment | GSM5252127, GSM5252129, GSM5252131,GSM5252133 | 4 | nFeature_RNA>200&nFeature_RNA<3000&nCount_RNA<10000 | 30,804 |
| PCa | Carcinoma tissue taken from the prostate cancer (PCa) patients | GSM4203181(10 samples), GSM4773521, GSM4773522, GSM5353236, GSM5353237 | 13 | nFeature_RNA>200&nFeature_RNA<4000&nCount_RNA<20000 | 33,909 |
| CRPC | Carcinoma tissue taken from the castration Resistant Prostate Cancer (CRPC ) patients | GSM4089151, GSM4089152, GSM4089153,GSM4089154, GSM4711414, GSM4711415 | 6 | nFeature_RNA>200&nFeature_RNA<6000&nCount_RNA<50000 | 25,046 |
| Total | − | − | 41 | − | 170438 |

**Supplementary Table S4.** Epithelial markers ranked by their information entropy

| **Marker** | **Normal_PZ** | **Normal_TZ** | **AN** | **Benign** | **BPH_GN** | **BPH_SN** | **PCa** | **CRPC** | **Rank sum** | **Total entropy** | 1**Total rank** |
| --- | --- | --- | --- | --- | --- | --- | --- | --- | --- | --- | --- |
| **Epithelial markers ranked by their information entropy** | | | | | | | | | | | |
| KRT8 | 1 | 3 | 6 | 2 | 1 | 1 | 2 | 2 | 18 | 93.073 | 2 |
| KRT18 | 4 | 4 | 2 | 1 | 2 | 2 | 1 | 1 | 17 | 93.606 | 1 |
| KRT15 | 6 | 7 | 5 | 5 | 4 | 5 | 7 | 7 | 46 | 36.591 | 6 |
| KRT17 | 3 | 1 | 8 | 6 | 3 | 3 | 8 | 8 | 40 | 41.182 | 5 |
| KRT19 | 2 | 2 | 3 | 4 | 6 | 6 | 5 | 4 | 32 | 47.511 | 4 |
| KRT7 | 7 | 6 | 7 | 8 | 8 | 7 | 6 | 6 | 55 | 17.011 | 8 |
| AGR2 | 8 | 8 | 4 | 7 | 7 | 8 | 4 | 5 | 51 | 28.19 | 7 |
| CLDN4 | 5 | 5 | 1 | 3 | 5 | 4 | 3 | 3 | 29 | 69.988 | 3 |
| **Stromal markers ranked by their information entropy** | | | | | | | | | | | |
| CLDN5 | 13 | 11 | 1 | 8 | 16 | 22 | 13 | 19 | 103 | 35.35 | 12 |
| SELE | 11 | 9 | 21 | 5 | 6 | 9 | 21 | 22 | 104 | 29.534 | 14 |
| VWF | 15 | 17 | 14 | 3 | 12 | 15 | 7 | 15 | 98 | 32.547 | 8 |
| ENG | 19 | 20 | 4 | 6 | 18 | 20 | 5 | 11 | 103 | 33.939 | 13 |
| IGFBP7 | 1 | 2 | 9 | 1 | 1 | 1 | 1 | 1 | 17 | 89.824 | 1 |
| IFI27 | 5 | 6 | 8 | 2 | 5 | 10 | 3 | 10 | 49 | 51.336 | 3 |
| EMCN | 23 | 23 | 15 | 7 | 22 | 21 | 9 | 20 | 140 | 22.534 | 20 |
| CD200 | 16 | 12 | 25 | 12 | 21 | 23 | 23 | 26 | 158 | 13.999 | 23 |
| C7 | 20 | 21 | 11 | 14 | 13 | 6 | 25 | 24 | 134 | 19.88 | 19 |
| VIM | 2 | 1 | 2 | 4 | 4 | 7 | 2 | 3 | 25 | 74.423 | 2 |
| PTGDS | 9 | 3 | 19 | 9 | 11 | 5 | 24 | 21 | 101 | 30.571 | 10 |
| GJA4 | 10 | 13 | 20 | 23 | 14 | 16 | 19 | 18 | 133 | 17.878 | 18 |
| RGS5 | 6 | 10 | 5 | 16 | 9 | 11 | 4 | 13 | 74 | 37.131 | 6 |
| MT1A | 4 | 5 | 24 | 18 | 2 | 2 | 22 | 25 | 102 | 31.336 | 11 |
| COL1A2 | 17 | 18 | 6 | 24 | 10 | 4 | 17 | 4 | 100 | 32.844 | 9 |
| MYH11 | 12 | 15 | 3 | 17 | 20 | 13 | 12 | 14 | 106 | 27.411 | 15 |
| ACTG2 | 21 | 19 | 22 | 22 | 26 | 26 | 26 | 23 | 185 | 6.37 | 26 |
| BGN | 8 | 8 | 16 | 20 | 15 | 14 | 10 | 2 | 93 | 34.888 | 7 |
| THY1 | 25 | 26 | 26 | 21 | 25 | 25 | 16 | 9 | 173 | 12.44 | 25 |
| PDGFRB | 22 | 22 | 18 | 26 | 24 | 19 | 14 | 12 | 157 | 16.773 | 22 |
| NRP1 | 26 | 24 | 17 | 10 | 23 | 24 | 18 | 17 | 159 | 15.166 | 24 |
| ANGPT2 | 18 | 16 | 23 | 19 | 8 | 12 | 15 | 16 | 127 | 18.796 | 17 |
| COL3A1 | 24 | 25 | 13 | 25 | 17 | 17 | 20 | 6 | 147 | 19.354 | 21 |
| COL4A1 | 3 | 4 | 10 | 13 | 3 | 3 | 6 | 7 | 49 | 47.275 | 4 |
| COL4A2 | 7 | 7 | 7 | 11 | 7 | 8 | 8 | 5 | 60 | 43.207 | 5 |
| COL18A1 | 14 | 14 | 12 | 15 | 19 | 18 | 11 | 8 | 111 | 27.857 | 16 |

^1^According to the sum of rank

**Supplementary Table S5.** Validating the performance of the 34 epithelial and stromal cell markers

|  | **Accuracy** | **Error** | **Precision** | **Recall** | **F1-score** |
| --- | --- | --- | --- | --- | --- |
| **Validating the performance of the eight epithelial marker genes** | | | | | |
| Normal_PZ | 1 | 0 | 1 | 1 | 1 |
| Normal_TZ | 1 | 0 | 1 | 1 | 1 |
| AN | 1 | 0 | 1 | 1 | 1 |
| Benign | 1 | 0 | 1 | 1 | 1 |
| BPH_GN | 1 | 0 | 1 | 1 | 1 |
| BPH_SN | 1 | 0 | 1 | 1 | 1 |
| ^1^ PCa | 0.923 | 0.077 | 1 | 1 | 1 |
| CRPC | 0.875 | 0.125 | 1 | 0.7 | 0.824 |
| **Validating the performance of the 26 stromal marker genes** | | | | | |
| Normal_PZ | 1 | 0 | 1 | 1 | 1 |
| Normal_TZ | 1 | 0 | 1 | 1 | 1 |
| AN | 0.929 | 0.071 | 0.8 | 1 | 0.889 |
| Benign | 1 | 0 | 1 | 1 | 1 |
| BPH_GN | 1 | 0 | 1 | 1 | 1 |
| ^2^ BPH_SN | 0.941 | 0.059 | 1 | 1 | 1 |
| ^3^ PCa | 0.923 | 0.077 | 1 | 1 | 1 |
| CRPC | 1 | 0 | 1 | 1 | 1 |

^1-2^The cell type of KMeans-cluster4 could not be determined;

^3^The cell type of KMeans-cluster5 could not be determined.

**Supplementary Table S6.** DEGA for the KMeans-clusters reclustered according to the eight epithelial markers

| ***p*_val** | **avg_log2FC** | **pct.1** | **pct.2** | ***p*_val_adj** | **KMeans-clusters** | **gene** |
| --- | --- | --- | --- | --- | --- | --- |
| **Normal_PZ** | | | | | | |
| 7.81E-177 | 0.636954911 | 0.928 | 0.752 | 1.87E-172 | 1 | *KRT8* |
| 3.55E-167 | 0.567161605 | 0.927 | 0.737 | 8.49E-163 | 1 | *KRT18* |
| 0 | 0.942408703 | 0.981 | 0.813 | 0 | 1 | *KRT17* |
| 0 | 0.8915286 | 0.935 | 0.697 | 0 | 1 | *KRT19* |
| 5.37E-282 | 0.628309082 | 0.88 | 0.6 | 1.28E-277 | 1 | *KRT7* |
| 2.97E-305 | 0.85636265 | 0.911 | 0.697 | 7.09E-301 | 1 | *CLDN4* |
| 9.05E-248 | 0.431719495 | 0.894 | 0.661 | 2.16E-243 | 2 | *KRT8* |
| 4.91E-238 | 0.378970499 | 0.881 | 0.649 | 1.17E-233 | 2 | *KRT18* |
| 0 | 2.116343271 | 0.838 | 0.401 | 0 | 2 | *KRT15* |
| 0 | 0.944057523 | 0.957 | 0.717 | 0 | 2 | *KRT17* |
| 6.34E-211 | 0.255339854 | 0.86 | 0.606 | 1.51E-206 | 2 | *KRT19* |
| 2.02E-130 | 0.557066804 | 0.728 | 0.564 | 4.84E-126 | 2 | *KRT7* |
| 0 | 1.417413438 | 0.193 | 0.005 | 0 | 3 | *CLDN5* |
| 0 | 2.09038768 | 0.174 | 0.004 | 0 | 3 | *SELE* |
| 0 | 1.156169659 | 0.205 | 0.005 | 0 | 3 | *VWF* |
| 0 | 0.851682056 | 0.16 | 0.008 | 0 | 3 | *ENG* |
| 0 | 4.152312637 | 0.717 | 0.229 | 0 | 3 | *IGFBP7* |
| 0 | 2.613685369 | 0.299 | 0.014 | 0 | 3 | *IFI27* |
| 0 | 0.622244714 | 0.135 | 0.001 | 0 | 3 | *EMCN* |
| 4.53E-304 | 1.00508507 | 0.203 | 0.037 | 1.08E-299 | 3 | *CD200* |
| 0 | 0.960333921 | 0.134 | 0.002 | 0 | 3 | *C7* |
| 0 | 3.896743152 | 0.849 | 0.083 | 0 | 3 | *VIM* |
| 0 | 3.287186378 | 0.194 | 0.022 | 0 | 3 | *PTGDS* |
| 0 | 1.862300458 | 0.223 | 0.007 | 0 | 3 | *GJA4* |
| 0 | 3.53718372 | 0.24 | 0.015 | 0 | 3 | *RGS5* |
| 0 | 3.392589821 | 0.301 | 0.046 | 0 | 3 | *MT1A* |
| 0 | 0.921034309 | 0.18 | 0.002 | 0 | 3 | *COL1A2* |
| 0 | 1.751004025 | 0.194 | 0.017 | 0 | 3 | *MYH11* |
| 7.10E-152 | 1.540974566 | 0.093 | 0.014 | 1.70E-147 | 3 | *ACTG2* |
| 0 | 1.682211046 | 0.285 | 0.006 | 0 | 3 | *BGN* |
| 0 | 0.450094175 | 0.098 | 0.001 | 0 | 3 | *THY1* |
| 0 | 0.684432798 | 0.14 | 0.001 | 0 | 3 | *PDGFRB* |
| 1.43E-125 | 0.383460206 | 0.129 | 0.034 | 3.43E-121 | 3 | *NRP1* |
| 0 | 1.183625515 | 0.154 | 0.016 | 0 | 3 | *ANGPT2* |
| 0 | 0.62385661 | 0.115 | 0.002 | 0 | 3 | *COL3A1* |
| 0 | 1.652657702 | 0.427 | 0.04 | 0 | 3 | *COL4A1* |
| 0 | 1.11392996 | 0.346 | 0.062 | 0 | 3 | *COL4A2* |
| 6.24E-240 | 0.854727864 | 0.262 | 0.08 | 1.49E-235 | 3 | *COL18A1* |
| 0 | 2.774841247 | 0.601 | 0.121 | 0 | 4 | *AGR2* |
| 0 | 1.447329697 | 0.94 | 0.703 | 0 | 4 | *CLDN4* |
| **Normal_TZ** | | | | | | |
| 6.68E-187 | 0.411100044 | 0.882 | 0.681 | 1.59E-182 | 1 | *KRT8* |
| 0 | 0.72059029 | 0.915 | 0.672 | 0 | 1 | *KRT18* |
| 2.71E-222 | 0.534080708 | 0.767 | 0.54 | 6.44E-218 | 1 | *KRT15* |
| 0 | 1.190715633 | 0.99 | 0.773 | 0 | 1 | *KRT17* |
| 0 | 0.829920316 | 0.933 | 0.677 | 0 | 1 | *KRT19* |
| 0 | 1.216741136 | 0.838 | 0.555 | 0 | 1 | *KRT7* |
| 4.50E-186 | 0.401171249 | 0.862 | 0.661 | 1.07E-181 | 1 | *CLDN4* |
| 1.66E-140 | 0.529629182 | 0.977 | 0.728 | 3.94E-136 | 2 | *KRT19* |
| 0 | 2.088046168 | 0.814 | 0.146 | 0 | 2 | *AGR2* |
| 0 | 1.015497326 | 0.972 | 0.686 | 0 | 2 | *CLDN4* |
| 0 | 1.578621301 | 0.216 | 0.004 | 0 | 3 | *CLDN5* |
| 0 | 2.363042515 | 0.205 | 0.005 | 0 | 3 | *SELE* |
| 0 | 0.872070421 | 0.183 | 0.003 | 0 | 3 | *VWF* |
| 0 | 0.714068718 | 0.184 | 0.009 | 0 | 3 | *ENG* |
| 0 | 3.616997433 | 0.768 | 0.306 | 0 | 3 | *IGFBP7* |
| 0 | 2.688649084 | 0.312 | 0.041 | 0 | 3 | *IFI27* |
| 0 | 0.572248116 | 0.142 | 0.002 | 0 | 3 | *EMCN* |
| 0 | 1.261057259 | 0.256 | 0.048 | 0 | 3 | *CD200* |
| 0 | 1.029041679 | 0.144 | 0.002 | 0 | 3 | *C7* |
| 0 | 4.278482839 | 0.897 | 0.045 | 0 | 3 | *VIM* |
| 0 | 3.971678855 | 0.302 | 0.026 | 0 | 3 | *PTGDS* |
| 0 | 1.807381623 | 0.168 | 0.005 | 0 | 3 | *GJA4* |
| 0 | 2.842050935 | 0.169 | 0.008 | 0 | 3 | *RGS5* |
| 0 | 2.930863341 | 0.301 | 0.035 | 0 | 3 | *MT1A* |
| 0 | 0.826528883 | 0.173 | 0.002 | 0 | 3 | *COL1A2* |
| 0 | 1.605578478 | 0.154 | 0.011 | 0 | 3 | *MYH11* |
| 2.91E-203 | 1.822129629 | 0.09 | 0.007 | 6.92E-199 | 3 | *ACTG2* |
| 0 | 1.476023758 | 0.291 | 0.003 | 0 | 3 | *BGN* |
| 1.39E-275 | 0.388447336 | 0.086 | 0.001 | 3.31E-271 | 3 | *THY1* |
| 0 | 0.730619518 | 0.151 | 0.002 | 0 | 3 | *PDGFRB* |
| 5.51E-201 | 0.386513449 | 0.153 | 0.028 | 1.31E-196 | 3 | *NRP1* |
| 0 | 1.344801986 | 0.156 | 0.014 | 0 | 3 | *ANGPT2* |
| 2.56E-301 | 0.451569781 | 0.096 | 0.002 | 6.09E-297 | 3 | *COL3A1* |
| 0 | 1.455021645 | 0.437 | 0.038 | 0 | 3 | *COL4A1* |
| 0 | 0.995407739 | 0.359 | 0.051 | 0 | 3 | *COL4A2* |
| 0 | 0.754917157 | 0.254 | 0.046 | 0 | 3 | *COL18A1* |
| 9.93E-53 | 1.59083812 | 0.528 | 0.253 | 2.36E-48 | 4 | *AGR2* |
| 9.72E-17 | 0.365696044 | 0.921 | 0.73 | 2.31E-12 | 4 | *CLDN4* |
| 4.55E-188 | 0.673944868 | 0.849 | 0.721 | 1.08E-183 | 5 | *KRT8* |
| 3.76E-276 | 1.021729511 | 0.768 | 0.572 | 8.95E-272 | 5 | *KRT15* |
| 7.42E-78 | 0.352262878 | 0.944 | 0.82 | 1.77E-73 | 5 | *KRT17* |
| AN | | | | | | |
| 1.07E-39 | 2.377007503 | 0.083 | 0.004 | 1.83E-35 | 1 | *SELE* |
| 1.48E-77 | 2.623736734 | 0.173 | 0.012 | 2.54E-73 | 1 | *VWF* |
| 6.00E-74 | 1.607813369 | 0.172 | 0.013 | 1.03E-69 | 1 | *ENG* |
| 2.19E-51 | 1.648699673 | 0.289 | 0.092 | 3.74E-47 | 1 | *IGFBP7* |
| 6.44E-42 | 1.266334762 | 0.123 | 0.016 | 1.10E-37 | 1 | *IFI27* |
| 1.83E-37 | 1.148721315 | 0.094 | 0.008 | 3.13E-33 | 1 | *EMCN* |
| 9.17E-07 | 0.324934176 | 0.026 | 0.006 | 0.015699456 | 1 | *CD200* |
| 3.49E-52 | 2.176459146 | 0.107 | 0.004 | 5.97E-48 | 1 | *C7* |
| 4.00E-195 | 1.960620331 | 0.714 | 0.199 | 6.85E-191 | 1 | *VIM* |
| 2.53E-29 | 1.089554442 | 0.051 | 0 | 4.34E-25 | 1 | *PTGDS* |
| 7.99E-43 | 2.455261076 | 0.124 | 0.016 | 1.37E-38 | 1 | *COL1A2* |
| 3.90E-10 | 0.668476494 | 0.06 | 0.019 | 6.67E-06 | 1 | *BGN* |
| 6.05E-07 | 0.422779134 | 0.048 | 0.018 | 0.010361314 | 1 | *PDGFRB* |
| 1.31E-31 | 1.153157934 | 0.156 | 0.042 | 2.25E-27 | 1 | *NRP1* |
| 6.13E-20 | 1.3475253 | 0.079 | 0.017 | 1.05E-15 | 1 | *COL3A1* |
| 1.02E-28 | 0.995892279 | 0.105 | 0.02 | 1.75E-24 | 1 | *COL4A1* |
| 4.75E-30 | 1.031470836 | 0.118 | 0.024 | 8.14E-26 | 1 | *COL4A2* |
| 1.61E-28 | 0.915934812 | 0.49 | 0.251 | 2.76E-24 | 2 | *KRT18* |
| 3.26E-34 | 1.208878952 | 0.374 | 0.148 | 5.58E-30 | 2 | *AGR2* |
| 3.65E-17 | 0.714461129 | 0.499 | 0.306 | 6.25E-13 | 2 | *CLDN4* |
| 3.73E-15 | 0.382606309 | 0.262 | 0.146 | 6.39E-11 | 3 | *KRT8* |
| 1.78E-26 | 0.562955397 | 0.402 | 0.221 | 3.04E-22 | 3 | *KRT18* |
| 4.82E-12 | 0.258315875 | 0.245 | 0.143 | 8.24E-08 | 3 | *AGR2* |
| 4.87E-23 | 0.465786185 | 0.132 | 0.039 | 8.34E-19 | 3 | *CLDN5* |
| 4.69E-80 | 3.861755717 | 0.857 | 0.063 | 8.04E-76 | 4 | *CLDN5* |
| 2.54E-10 | 0.71238947 | 0.314 | 0.054 | 4.34E-06 | 4 | *VWF* |
| 2.21E-21 | 1.994490961 | 0.429 | 0.053 | 3.78E-17 | 4 | *ENG* |
| 5.64E-18 | 1.485189041 | 0.686 | 0.142 | 9.65E-14 | 4 | *IGFBP7* |
| 4.98E-111 | 3.515350229 | 0.829 | 0.038 | 8.53E-107 | 4 | *IFI27* |
| 5.39E-66 | 2.51190222 | 0.543 | 0.027 | 9.22E-62 | 4 | *EMCN* |
| 2.32E-23 | 2.183254925 | 1 | 0.336 | 3.97E-19 | 4 | *VIM* |
| 8.06E-46 | 2.013478519 | 0.314 | 0.013 | 1.38E-41 | 4 | *GJA4* |
| 4.69E-20 | 1.793784875 | 0.371 | 0.042 | 8.03E-16 | 4 | *RGS5* |
| 6.37E-10 | 1.267441391 | 0.257 | 0.041 | 1.09E-05 | 4 | *COL4A1* |
| 3.76E-07 | 1.12316079 | 0.314 | 0.078 | 0.006446207 | 4 | *COL18A1* |
| 3.67E-23 | 1.010244384 | 0.358 | 0.162 | 6.28E-19 | 5 | *KRT8* |
| 1.27E-10 | 0.486525615 | 0.421 | 0.264 | 2.18E-06 | 5 | *KRT18* |
| 0 | 4.306241477 | 0.854 | 0.05 | 0 | 5 | *KRT15* |
| 9.84E-92 | 1.370328094 | 0.182 | 0.007 | 1.69E-87 | 5 | *KRT17* |
| 7.79E-174 | 2.582494851 | 0.676 | 0.129 | 1.33E-169 | 5 | *KRT19* |
| 2.05E-69 | 1.490254589 | 0.263 | 0.037 | 3.52E-65 | 5 | *KRT7* |
| 1.99E-10 | 0.561982978 | 0.462 | 0.314 | 3.42E-06 | 5 | *CLDN4* |
| 3.85E-17 | 0.412619754 | 0.19 | 0.065 | 6.58E-13 | 5 | *COL18A1* |
| 6.18E-13 | 0.886847022 | 0.399 | 0.176 | 1.06E-08 | 6 | *KRT8* |
| 1.86E-12 | 0.835193864 | 0.523 | 0.272 | 3.18E-08 | 6 | *KRT18* |
| 2.63E-12 | 0.95522351 | 0.346 | 0.136 | 4.51E-08 | 6 | *KRT15* |
| 1.33E-06 | 0.374211717 | 0.092 | 0.025 | 0.022745503 | 6 | *KRT17* |
| 3.75E-77 | 2.729371655 | 0.725 | 0.169 | 6.42E-73 | 6 | *KRT19* |
| 1.06E-107 | 2.594611092 | 0.484 | 0.044 | 1.81E-103 | 6 | *KRT7* |
| 1.35E-59 | 2.166763962 | 0.641 | 0.157 | 2.32E-55 | 6 | *AGR2* |
| 6.60E-69 | 2.447376465 | 0.863 | 0.307 | 1.13E-64 | 6 | *CLDN4* |
| 2.09E-42 | 2.253691073 | 0.416 | 0.126 | 3.58E-38 | 7 | *IGFBP7* |
| 1.23E-88 | 1.986402636 | 0.855 | 0.302 | 2.11E-84 | 7 | *VIM* |
| 7.51E-59 | 1.630685709 | 0.137 | 0.006 | 1.29E-54 | 7 | *GJA4* |
| 3.56E-161 | 4.560113182 | 0.38 | 0.019 | 6.10E-157 | 7 | *RGS5* |
| 1.88E-14 | 0.76476614 | 0.059 | 0.007 | 3.22E-10 | 7 | *MT1A* |
| 1.62E-09 | 0.259607827 | 0.125 | 0.04 | 2.77E-05 | 7 | *COL1A2* |
| 5.07E-169 | 6.013994765 | 0.408 | 0.022 | 8.68E-165 | 7 | *MYH11* |
| 1.76E-32 | 1.021696801 | 0.09 | 0.006 | 3.01E-28 | 7 | *ACTG2* |
| 1.77E-40 | 1.389348358 | 0.169 | 0.02 | 3.04E-36 | 7 | *BGN* |
| 7.01E-09 | 0.446020001 | 0.039 | 0.006 | 0.000120038 | 7 | *THY1* |
| 1.12E-46 | 1.454863008 | 0.165 | 0.016 | 1.91E-42 | 7 | *PDGFRB* |
| 1.81E-10 | 0.380764969 | 0.059 | 0.01 | 3.11E-06 | 7 | *ANGPT2* |
| 4.47E-18 | 1.191158694 | 0.129 | 0.027 | 7.66E-14 | 7 | *COL3A1* |
| 9.43E-09 | 0.663608155 | 0.114 | 0.038 | 0.000161514 | 7 | *COL4A1* |
| 3.41E-17 | 1.149666434 | 0.161 | 0.042 | 5.84E-13 | 7 | *COL4A2* |
| 9.86E-24 | 1.497091102 | 0.239 | 0.067 | 1.69E-19 | 7 | *COL18A1* |
| **Benign** | | | | | | |
| 5.31E-51 | 0.97124197 | 0.973 | 0.736 | 1.12E-46 | 1 | *KRT8* |
| 3.22E-37 | 0.940801899 | 0.961 | 0.736 | 6.82E-33 | 1 | *KRT18* |
| 1.72E-135 | 2.299551895 | 0.919 | 0.343 | 3.64E-131 | 1 | *KRT15* |
| 8.87E-169 | 3.81121856 | 0.896 | 0.313 | 1.88E-164 | 1 | *KRT17* |
| 2.89E-85 | 2.404909313 | 0.785 | 0.407 | 6.12E-81 | 1 | *KRT19* |
| 1.17E-47 | 0.971686881 | 0.463 | 0.162 | 2.47E-43 | 1 | *KRT7* |
| 3.3E-12 | 0.760621594 | 0.669 | 0.516 | 6.99E-08 | 1 | *CLDN4* |
| 4.09E-61 | 0.273861113 | 0.537 | 0.18 | 8.65E-57 | 1 | *IGFBP7* |
| 3.28E-15 | 0.34833996 | 0.313 | 0.151 | 6.94E-11 | 1 | *COL18A1* |
| 2.44E-35 | 1.045156082 | 0.064 | 0.007 | 5.16E-31 | 2 | *SELE* |
| 3.04E-39 | 0.719642235 | 0.079 | 0.011 | 6.43E-35 | 2 | *VWF* |
| 3.59E-76 | 0.679774995 | 0.177 | 0.031 | 7.6E-72 | 2 | *ENG* |
| 3.78E-33 | 1.317820775 | 0.111 | 0.03 | 8E-29 | 2 | *IFI27* |
| 8.18E-52 | 0.312700942 | 0.064 | 0.001 | 1.73E-47 | 2 | *EMCN* |
| 2.16E-292 | 2.332982347 | 0.871 | 0.469 | 4.58E-288 | 2 | *VIM* |
| 3.97E-19 | 0.361189303 | 0.044 | 0.008 | 8.41E-15 | 2 | *PTGDS* |
| 1.03E-09 | 1.53258717 | 0.072 | 0.034 | 0.0000217 | 2 | *MT1A* |
| 1.15E-64 | 0.309970305 | 0.936 | 0.645 | 2.43E-60 | 3 | *KRT8* |
| 4.04E-106 | 0.566352304 | 0.942 | 0.641 | 8.55E-102 | 3 | *KRT18* |
| 1.56E-138 | 0.286801978 | 0.767 | 0.386 | 3.31E-134 | 3 | *CLDN4* |
| 4.42E-45 | 1.274066882 | 0.981 | 0.739 | 9.36E-41 | 4 | *KRT8* |
| 7.14E-54 | 1.218643696 | 0.988 | 0.738 | 1.51E-49 | 4 | *KRT18* |
| 4.2E-58 | 2.209776458 | 0.812 | 0.411 | 8.89E-54 | 4 | *KRT19* |
| 6.13E-120 | 2.233675702 | 0.685 | 0.154 | 1.3E-115 | 4 | *KRT7* |
| 2.1E-137 | 2.849838606 | 0.796 | 0.209 | 4.44E-133 | 4 | *AGR2* |
| 1.76E-106 | 2.660221886 | 0.962 | 0.502 | 3.73E-102 | 4 | *CLDN4* |
| 0 | 2.1018741 | 0.874 | 0.196 | 0 | 5 | *KRT15* |
| 2.39E-254 | 0.688463806 | 0.716 | 0.216 | 5.05E-250 | 5 | *KRT17* |
| 3.16E-220 | 0.676641175 | 0.773 | 0.304 | 6.7E-216 | 5 | *KRT19* |
| 1.4E-83 | 0.329817379 | 0.359 | 0.115 | 2.97E-79 | 5 | *KRT7* |
| 1.79E-110 | 0.481798512 | 0.347 | 0.092 | 3.79E-106 | 5 | *COL18A1* |
| **BPH_GN** | | | | | | |
| 0 | 1.273381523 | 0.135 | 0.013 | 0 | 1 | *CLDN5* |
| 0 | 2.825454266 | 0.151 | 0.021 | 0 | 1 | *SELE* |
| 0 | 1.317171574 | 0.17 | 0.01 | 0 | 1 | *VWF* |
| 0 | 0.828151221 | 0.202 | 0.011 | 0 | 1 | *ENG* |
| 0 | 3.307601213 | 0.538 | 0.337 | 0 | 1 | *IGFBP7* |
| 0 | 2.16788389 | 0.212 | 0.027 | 0 | 1 | *IFI27* |
| 0 | 0.724836843 | 0.132 | 0.004 | 0 | 1 | *EMCN* |
| 1.34E-222 | 0.740828932 | 0.148 | 0.042 | 3.44E-218 | 1 | *CD200* |
| 0 | 1.371292859 | 0.156 | 0.008 | 0 | 1 | *C7* |
| 0 | 3.32577784 | 0.875 | 0.339 | 0 | 1 | *VIM* |
| 1.70E-244 | 2.163150409 | 0.121 | 0.021 | 4.38E-240 | 1 | *PTGDS* |
| 0 | 1.36303374 | 0.144 | 0.008 | 0 | 1 | *GJA4* |
| 0 | 2.480587292 | 0.153 | 0.017 | 0 | 1 | *RGS5* |
| 0 | 3.351412593 | 0.282 | 0.058 | 0 | 1 | *MT1A* |
| 0 | 1.47252909 | 0.21 | 0.008 | 0 | 1 | *COL1A2* |
| 6.02E-279 | 0.988960512 | 0.129 | 0.019 | 1.55E-274 | 1 | *MYH11* |
| 5.27E-146 | 0.38918075 | 0.055 | 0.003 | 1.36E-141 | 1 | *ACTG2* |
| 0 | 1.019274944 | 0.166 | 0.009 | 0 | 1 | *BGN* |
| 0 | 0.591289653 | 0.122 | 0.003 | 0 | 1 | *THY1* |
| 0 | 0.61765323 | 0.135 | 0.003 | 0 | 1 | *PDGFRB* |
| 7.60E-192 | 0.549148416 | 0.176 | 0.069 | 1.96E-187 | 1 | *NRP1* |
| 0 | 1.741455157 | 0.214 | 0.02 | 0 | 1 | *ANGPT2* |
| 0 | 0.87597797 | 0.136 | 0.006 | 0 | 1 | *COL3A1* |
| 0 | 1.389363917 | 0.333 | 0.11 | 0 | 1 | *COL4A1* |
| 0 | 1.008613959 | 0.29 | 0.1 | 0 | 1 | *COL4A2* |
| 1.72E-88 | 0.463522541 | 0.195 | 0.121 | 4.42E-84 | 1 | *COL18A1* |
| 0 | 1.930284148 | 0.961 | 0.395 | 0 | 2 | *KRT8* |
| 0 | 1.79020045 | 0.966 | 0.432 | 0 | 2 | *KRT18* |
| 1.75E-244 | 0.980229306 | 0.554 | 0.257 | 4.52E-240 | 2 | *KRT15* |
| 0 | 2.364612606 | 0.456 | 0.14 | 0 | 2 | *KRT19* |
| 0 | 1.62549906 | 0.556 | 0.066 | 0 | 2 | *KRT7* |
| 0 | 3.290527915 | 0.716 | 0.063 | 0 | 2 | *AGR2* |
| 0 | 3.33967286 | 0.952 | 0.212 | 0 | 2 | *CLDN4* |
| 0 | 1.107506004 | 0.913 | 0.321 | 0 | 3 | *KRT8* |
| 0 | 0.866332581 | 0.859 | 0.378 | 0 | 3 | *KRT18* |
| 0 | 1.263273937 | 0.627 | 0.192 | 0 | 3 | *KRT15* |
| 0 | 1.477959117 | 0.738 | 0.285 | 0 | 3 | *KRT17* |
| 0 | 1.001072176 | 0.954 | 0.401 | 0 | 4 | *KRT8* |
| 0 | 1.395708145 | 0.966 | 0.438 | 0 | 4 | *KRT18* |
| 0 | 2.08910121 | 0.821 | 0.238 | 0 | 4 | *KRT15* |
| 0 | 3.224306253 | 0.978 | 0.33 | 0 | 4 | *KRT17* |
| 0 | 2.152063856 | 0.476 | 0.142 | 0 | 4 | *KRT19* |
| 2.77E-227 | 0.765325269 | 0.303 | 0.092 | 7.14E-223 | 4 | *KRT7* |
| 0 | 1.578147253 | 0.684 | 0.241 | 0 | 4 | *CLDN4* |
| 2.40E-212 | 0.912203968 | 0.868 | 0.424 | 6.19E-208 | 5 | *KRT8* |
| 4.54E-160 | 0.648974948 | 0.869 | 0.46 | 1.17E-155 | 5 | *KRT18* |
| 0 | 1.197003465 | 0.719 | 0.255 | 0 | 5 | *CLDN4* |
| **BPH_SN** | | | | | | |
| 0 | 2.888184727 | 0.918 | 0.051 | 0 | 1 | *KRT8* |
| 0 | 2.650162465 | 0.883 | 0.086 | 0 | 1 | *KRT18* |
| 0 | 2.132802787 | 0.402 | 0.013 | 0 | 1 | *KRT15* |
| 0 | 4.359003655 | 0.786 | 0.08 | 0 | 1 | *KRT17* |
| 1.19E-193 | 2.515693984 | 0.243 | 0.058 | 3.10E-189 | 1 | *KRT19* |
| 0 | 1.367178138 | 0.193 | 0.013 | 0 | 1 | *KRT7* |
| 1.12E-146 | 0.387732315 | 0.094 | 0.011 | 2.92E-142 | 1 | *AGR2* |
| 0 | 1.412384407 | 0.259 | 0.034 | 0 | 1 | *CLDN4* |
| 0 | 3.005571395 | 0.907 | 0.089 | 0 | 2 | *KRT8* |
| 0 | 2.812017911 | 0.884 | 0.121 | 0 | 2 | *KRT18* |
| 9.33E-81 | 0.920186379 | 0.248 | 0.032 | 2.43E-76 | 2 | *KRT15* |
| 4.12E-18 | 1.28100677 | 0.295 | 0.115 | 1.07E-13 | 2 | *KRT17* |
| 1.56E-294 | 3.726932636 | 0.616 | 0.063 | 4.06E-290 | 2 | *KRT19* |
| 0 | 2.167450217 | 0.519 | 0.019 | 0 | 2 | *KRT7* |
| 0 | 3.106973307 | 0.698 | 0.01 | 0 | 2 | *AGR2* |
| 0 | 4.510711517 | 0.903 | 0.039 | 0 | 2 | *CLDN4* |
| 5.96E-30 | 0.668994962 | 0.079 | 0.008 | 1.55E-25 | 3 | *CLDN5* |
| 4.90E-65 | 2.53527711 | 0.178 | 0.028 | 1.27E-60 | 3 | *SELE* |
| 3.85E-49 | 0.966946796 | 0.123 | 0.011 | 1.00E-44 | 3 | *VWF* |
| 9.72E-59 | 0.692224277 | 0.146 | 0.014 | 2.53E-54 | 3 | *ENG* |
| 1.22E-73 | 1.899706446 | 0.652 | 0.686 | 3.17E-69 | 3 | *IGFBP7* |
| 5.13E-62 | 1.906409851 | 0.193 | 0.045 | 1.33E-57 | 3 | *IFI27* |
| 4.36E-36 | 0.523126761 | 0.086 | 0.005 | 1.13E-31 | 3 | *EMCN* |
| 1.06E-18 | 0.5219004 | 0.096 | 0.037 | 2.75E-14 | 3 | *CD200* |
| 1.98E-117 | 1.77119538 | 0.255 | 0.021 | 5.15E-113 | 3 | *C7* |
| 0 | 2.752683085 | 0.881 | 0.583 | 0 | 3 | *VIM* |
| 4.35E-69 | 3.176936052 | 0.333 | 0.176 | 1.13E-64 | 3 | *PTGDS* |
| 1.98E-45 | 1.028573309 | 0.114 | 0.01 | 5.16E-41 | 3 | *GJA4* |
| 5.97E-46 | 1.968094719 | 0.151 | 0.035 | 1.55E-41 | 3 | *RGS5* |
| 7.14E-161 | 3.26688218 | 0.476 | 0.21 | 1.86E-156 | 3 | *MT1A* |
| 1.46E-139 | 1.647210273 | 0.284 | 0.018 | 3.79E-135 | 3 | *COL1A2* |
| 7.49E-72 | 1.278603279 | 0.215 | 0.046 | 1.95E-67 | 3 | *MYH11* |
| 1.80E-52 | 0.718851983 | 0.154 | 0.026 | 4.67E-48 | 3 | *ACTG2* |
| 4.22E-58 | 0.916881542 | 0.145 | 0.014 | 1.10E-53 | 3 | *BGN* |
| 6.49E-28 | 0.345659416 | 0.062 | 0.001 | 1.69E-23 | 3 | *THY1* |
| 1.55E-49 | 0.551491082 | 0.114 | 0.005 | 4.04E-45 | 3 | *PDGFRB* |
| 3.16E-18 | 0.361423069 | 0.117 | 0.054 | 8.23E-14 | 3 | *NRP1* |
| 1.30E-51 | 1.290229412 | 0.151 | 0.027 | 3.39E-47 | 3 | *ANGPT2* |
| 1.49E-50 | 0.761185458 | 0.124 | 0.01 | 3.87E-46 | 3 | *COL3A1* |
| 1.17E-40 | 0.992626357 | 0.295 | 0.182 | 3.04E-36 | 3 | *COL4A1* |
| 6.91E-20 | 0.698178662 | 0.254 | 0.189 | 1.80E-15 | 3 | *COL4A2* |
| **PCa** | | | | | | |
| 8.66E-39 | 1.096005464 | 0.581 | 0.291 | 2.41E-34 | 1 | *IGFBP7* |
| 3.49E-224 | 1.715944489 | 0.274 | 0.02 | 9.72E-220 | 1 | *C7* |
| 9.56E-32 | 1.022948568 | 0.642 | 0.412 | 2.66E-27 | 1 | *VIM* |
| 7.62E-113 | 3.355527854 | 0.246 | 0.032 | 2.12E-108 | 1 | *PTGDS* |
| 0 | 4.091409781 | 0.536 | 0.049 | 0 | 1 | *COL1A2* |
| 7.68E-22 | 0.616537609 | 0.092 | 0.02 | 2.14E-17 | 1 | *ACTG2* |
| 5.01E-306 | 2.685704228 | 0.62 | 0.081 | 1.39E-301 | 1 | *BGN* |
| 1.00E-120 | 1.261850415 | 0.358 | 0.058 | 2.80E-116 | 1 | *THY1* |
| 1.48E-84 | 0.809615667 | 0.321 | 0.061 | 4.13E-80 | 1 | *PDGFRB* |
| 5.62E-304 | 3.437299853 | 0.453 | 0.042 | 1.57E-299 | 1 | *COL3A1* |
| 1.22E-19 | 0.768393111 | 0.254 | 0.103 | 3.39E-15 | 1 | *COL4A1* |
| 1.48E-43 | 0.686549041 | 0.335 | 0.103 | 4.12E-39 | 1 | *COL4A2* |
| 2.74E-36 | 0.642775299 | 0.341 | 0.121 | 7.62E-32 | 1 | *COL18A1* |
| 0 | 0.880416831 | 0.881 | 0.488 | 0 | 2 | *KRT8* |
| 0 | 0.959122493 | 0.907 | 0.542 | 0 | 2 | *KRT18* |
| 0 | 1.730065786 | 0.138 | 0.019 | 0 | 2 | *KRT15* |
| 3.18E-227 | 2.198771231 | 0.127 | 0.027 | 8.85E-223 | 2 | *KRT17* |
| 4.55E-273 | 1.799585591 | 0.49 | 0.27 | 1.27E-268 | 2 | *KRT19* |
| 0 | 1.111584702 | 0.184 | 0.024 | 0 | 2 | *KRT7* |
| 0 | 1.548542057 | 0.809 | 0.419 | 0 | 2 | *CLDN4* |
| 0 | 1.12463697 | 0.792 | 0.335 | 0 | 3 | *KRT8* |
| 0 | 1.203059301 | 0.846 | 0.383 | 0 | 3 | *KRT18* |
| 0 | 1.68297777 | 0.605 | 0.187 | 0 | 3 | *AGR2* |
| 0 | 0.462403446 | 0.658 | 0.318 | 0 | 3 | *CLDN4* |
| 9.79E-62 | 1.3349203 | 0.295 | 0.039 | 2.73E-57 | 4 | *KRT17* |
| 9.91E-32 | 1.83479813 | 0.596 | 0.297 | 2.76E-27 | 4 | *KRT19* |
| 6.18E-40 | 1.520660405 | 0.256 | 0.044 | 1.72E-35 | 4 | *KRT7* |
| 2.02E-27 | 1.627769545 | 0.686 | 0.468 | 5.63E-23 | 4 | *CLDN4* |
| 1.59E-14 | 0.938345484 | 0.244 | 0.082 | 4.44E-10 | 4 | *CLDN5* |
| 1.01E-37 | 1.571856638 | 0.244 | 0.041 | 2.82E-33 | 4 | *SELE* |
| 3.25E-08 | 0.830121412 | 0.269 | 0.13 | 0.00090354 | 4 | *VWF* |
| 3.02E-93 | 1.803145381 | 0.795 | 0.176 | 8.40E-89 | 4 | *IFI27* |
| 3.44E-17 | 0.526733936 | 0.776 | 0.413 | 9.58E-13 | 4 | *VIM* |
| 0 | 1.412660288 | 0.184 | 0.027 | 0 | 5 | *CLDN5* |
| 0 | 0.972996349 | 0.099 | 0.01 | 0 | 5 | *SELE* |
| 0 | 1.881675817 | 0.287 | 0.044 | 0 | 5 | *VWF* |
| 0 | 1.656872435 | 0.306 | 0.026 | 0 | 5 | *ENG* |
| 0 | 3.948636602 | 0.591 | 0.128 | 0 | 5 | *IGFBP7* |
| 0 | 2.948448208 | 0.398 | 0.057 | 0 | 5 | *IFI27* |
| 0 | 1.504788923 | 0.259 | 0.009 | 0 | 5 | *EMCN* |
| 0 | 0.37240947 | 0.096 | 0.004 | 0 | 5 | *CD200* |
| 0 | 2.465686205 | 0.853 | 0.171 | 0 | 5 | *VIM* |
| 1.71E-186 | 0.349088869 | 0.073 | 0.013 | 4.76E-182 | 5 | *PTGDS* |
| 0 | 0.883417313 | 0.157 | 0.005 | 0 | 5 | *GJA4* |
| 0 | 3.22850394 | 0.268 | 0.046 | 0 | 5 | *RGS5* |
| 0 | 0.773266935 | 0.11 | 0.011 | 0 | 5 | *MT1A* |
| 0 | 0.439897914 | 0.128 | 0.014 | 0 | 5 | *COL1A2* |
| 0 | 1.885094783 | 0.179 | 0.017 | 0 | 5 | *MYH11* |
| 3.59E-192 | 0.360902988 | 0.051 | 0.004 | 9.99E-188 | 5 | *ACTG2* |
| 0 | 1.105216797 | 0.203 | 0.022 | 0 | 5 | *BGN* |
| 0 | 0.919408901 | 0.151 | 0.011 | 0 | 5 | *THY1* |
| 0 | 1.078053035 | 0.162 | 0.009 | 0 | 5 | *PDGFRB* |
| 5.13E-138 | 0.524375056 | 0.214 | 0.124 | 1.43E-133 | 5 | *NRP1* |
| 0 | 0.993353962 | 0.183 | 0.032 | 0 | 5 | *ANGPT2* |
| 0 | 0.568289064 | 0.106 | 0.013 | 0 | 5 | *COL3A1* |
| 0 | 1.683176866 | 0.266 | 0.014 | 0 | 5 | *COL4A1* |
| 0 | 1.449124789 | 0.266 | 0.016 | 0 | 5 | *COL4A2* |
| 0 | 0.868858735 | 0.24 | 0.058 | 0 | 5 | *COL18A1* |
| 9.69E-25 | 0.431041582 | 0.907 | 0.586 | 2.70E-20 | 6 | *KRT18* |
| 1.22E-08 | 0.334828073 | 0.53 | 0.372 | 0.000339172 | 6 | *AGR2* |
| 0 | 1.353351921 | 0.922 | 0.391 | 0 | 7 | *VIM* |
| 5.67E-13 | 0.373072954 | 0.353 | 0.034 | 1.58E-08 | 8 | *KRT15* |
| 7.78E-70 | 1.249588214 | 0.882 | 0.04 | 2.16E-65 | 8 | *KRT17* |
| 2.29E-10 | 1.657934554 | 1 | 0.469 | 6.37E-06 | 8 | *CLDN4* |
| 2.29E-17 | 0.463718951 | 0.412 | 0.034 | 6.36E-13 | 8 | *PTGDS* |
| **CRPC** | | | | | | |
| 2.29E-83 | 0.577780637 | 0.927 | 0.612 | 4.62E-79 | 1 | *KRT8* |
| 2.6E-45 | 0.360472142 | 0.919 | 0.618 | 5.24E-41 | 1 | *KRT18* |
| 0 | 3.300174845 | 0.776 | 0.075 | 0 | 1 | *KRT15* |
| 0 | 3.760353765 | 0.796 | 0.107 | 0 | 1 | *KRT17* |
| 0 | 2.929956758 | 0.93 | 0.306 | 0 | 1 | *KRT19* |
| 0 | 1.487782269 | 0.621 | 0.11 | 0 | 1 | *KRT7* |
| 1.05E-105 | 1.23809085 | 0.868 | 0.552 | 2.11E-101 | 1 | *CLDN4* |
| 7.15E-38 | 0.642436351 | 0.066 | 0.025 | 1.44E-33 | 2 | *CLDN5* |
| 6.72E-13 | 0.847624308 | 0.1 | 0.076 | 1.35E-08 | 2 | *VWF* |
| 8.62E-156 | 0.703115326 | 0.176 | 0.049 | 1.74E-151 | 2 | *ENG* |
| 3.76E-195 | 3.208481376 | 0.496 | 0.385 | 7.58E-191 | 2 | *IGFBP7* |
| 7.74E-62 | 0.252770225 | 0.063 | 0.013 | 1.56E-57 | 2 | *EMCN* |
| 0 | 3.027620293 | 0.646 | 0.425 | 0 | 2 | *VIM* |
| 8.13E-73 | 0.424910043 | 0.07 | 0.012 | 1.64E-68 | 2 | *GJA4* |
| 4.04E-41 | 1.454859058 | 0.145 | 0.089 | 8.14E-37 | 2 | *RGS5* |
| 5.23E-119 | 2.022548936 | 0.225 | 0.114 | 1.05E-114 | 2 | *COL1A2* |
| 8.89E-48 | 0.778846994 | 0.093 | 0.04 | 1.79E-43 | 2 | *MYH11* |
| 2.31E-36 | 0.477044089 | 0.054 | 0.018 | 4.65E-32 | 2 | *ACTG2* |
| 1.02E-151 | 1.876772779 | 0.255 | 0.124 | 2.05E-147 | 2 | *BGN* |
| 2.18E-158 | 0.943121562 | 0.149 | 0.03 | 4.4E-154 | 2 | *THY1* |
| 3.4E-130 | 0.52816723 | 0.13 | 0.027 | 6.84E-126 | 2 | *PDGFRB* |
| 3.15E-125 | 1.706533129 | 0.197 | 0.083 | 6.34E-121 | 2 | *COL3A1* |
| 7.4E-130 | 1.022285276 | 0.18 | 0.066 | 1.49E-125 | 2 | *COL4A1* |
| 6.03E-132 | 1.128913078 | 0.204 | 0.085 | 1.22E-127 | 2 | *COL4A2* |
| 1.53E-237 | 0.875462476 | 0.976 | 0.604 | 3.08E-233 | 3 | *KRT8* |
| 1.32E-224 | 0.911207741 | 0.978 | 0.61 | 2.67E-220 | 3 | *KRT18* |
| 0 | 0.828376914 | 0.423 | 0.079 | 0 | 3 | *KRT15* |
| 0 | 2.012068957 | 0.633 | 0.102 | 0 | 3 | *KRT17* |
| 0 | 2.688271582 | 0.966 | 0.293 | 0 | 3 | *KRT19* |
| 0 | 2.516676433 | 0.868 | 0.089 | 0 | 3 | *KRT7* |
| 0 | 1.387214035 | 0.816 | 0.169 | 0 | 3 | *AGR2* |
| 0 | 1.889286189 | 0.975 | 0.542 | 0 | 3 | *CLDN4* |
| 0 | 1.033454575 | 0.974 | 0.539 | 0 | 4 | *KRT8* |
| 0 | 0.705765234 | 0.979 | 0.546 | 0 | 4 | *KRT18* |
| 0 | 1.621364623 | 0.429 | 0.145 | 0 | 4 | *AGR2* |
| 0 | 0.780094916 | 0.946 | 0.473 | 0 | 4 | *CLDN4* |
| 5.73E-93 | 3.215221912 | 0.864 | 0.618 | 1.15E-88 | 5 | *KRT8* |
| 2.39E-123 | 3.765425477 | 0.934 | 0.624 | 4.83E-119 | 5 | *KRT18* |
| 1.38E-60 | 2.779477696 | 0.662 | 0.32 | 2.77E-56 | 5 | *KRT19* |
| 1.11E-55 | 2.476974084 | 0.531 | 0.195 | 2.23E-51 | 5 | *AGR2* |
| 7.57E-31 | 2.126574906 | 0.658 | 0.56 | 1.53E-26 | 5 | *CLDN4* |
| 3.92E-99 | 2.387218499 | 0.864 | 0.36 | 7.89E-95 | 5 | *IFI27* |

Note: A *p*-value of "0" indicates an extremely small value approaching zero.

**Supplementary Table S7.** DEGA for the KMeans-clusters reclustered according to the 26 stromal marker genes

| ***p*_val** | **avg_log2FC** | **pct.1** | **pct.2** | ***p*_val_adj** | **KMeans-cluster** | **gene** |
| --- | --- | --- | --- | --- | --- | --- |
| **Normal_PZ** | | | | | | |
| 0 | 3.839132092 | 0.989 | 0.282 | 0 | 1 | *IGFBP7* |
| 0 | 2.643891445 | 0.916 | 0.181 | 0 | 1 | *VIM* |
| 0 | 3.450195723 | 0.774 | 0.012 | 0 | 1 | *GJA4* |
| 0 | 5.303899463 | 0.839 | 0.02 | 0 | 1 | *RGS5* |
| 0 | 5.066321683 | 0.829 | 0.058 | 0 | 1 | *MT1A* |
| 0 | 1.139636707 | 0.337 | 0.019 | 0 | 1 | *COL1A2* |
| 0 | 2.89344135 | 0.618 | 0.023 | 0 | 1 | *MYH11* |
| 3.07E-204 | 1.616887238 | 0.197 | 0.02 | 7.32E-200 | 1 | *ACTG2* |
| 0 | 2.794184578 | 0.698 | 0.026 | 0 | 1 | *BGN* |
| 0 | 0.793947323 | 0.201 | 0.01 | 0 | 1 | *THY1* |
| 0 | 1.471958316 | 0.397 | 0.009 | 0 | 1 | *PDGFRB* |
| 6.67E-46 | 0.508000515 | 0.153 | 0.045 | 1.59E-41 | 1 | *NRP1* |
| 2.79E-92 | 0.841768253 | 0.173 | 0.034 | 6.67E-88 | 1 | *ANGPT2* |
| 0 | 1.01046782 | 0.236 | 0.012 | 0 | 1 | *COL3A1* |
| 0 | 2.219527222 | 0.681 | 0.08 | 0 | 1 | *COL4A1* |
| 0 | 1.675334535 | 0.568 | 0.09 | 0 | 1 | *COL4A2* |
| 0 | 1.630661918 | 0.537 | 0.092 | 0 | 1 | *COL18A1* |
| 7.70E-36 | 2.400029745 | 0.855 | 0.308 | 1.84E-31 | 2 | *IGFBP7* |
| 1.55E-79 | 3.765750524 | 0.986 | 0.207 | 3.71E-75 | 2 | *VIM* |
| 2.65E-105 | 2.358740387 | 0.478 | 0.03 | 6.33E-101 | 2 | *COL1A2* |
| 3.83E-57 | 1.692417206 | 0.29 | 0.02 | 9.15E-53 | 2 | *COL3A1* |
| 1.96E-16 | 1.457533329 | 0.391 | 0.103 | 4.68E-12 | 2 | *COL4A1* |
| 9.28E-15 | 1.469622672 | 0.377 | 0.108 | 2.22E-10 | 2 | *COL4A2* |
| 4.78E-43 | 1.407533301 | 0.986 | 0.308 | 1.14E-38 | 3 | *IGFBP7* |
| 4.03E-132 | 3.210148181 | 0.681 | 0.048 | 9.63E-128 | 3 | *PTGDS* |
| 3.01E-39 | 1.006560183 | 0.304 | 0.03 | 7.19E-35 | 3 | *COL1A2* |
| 0 | 4.533647162 | 0.986 | 0.043 | 0 | 3 | *MYH11* |
| 0 | 5.715524814 | 0.971 | 0.024 | 0 | 3 | *ACTG2* |
| 1.02E-12 | 0.561711855 | 0.246 | 0.052 | 2.45E-08 | 3 | *BGN* |
| 2.85E-50 | 0.825750187 | 0.275 | 0.02 | 6.80E-46 | 3 | *COL3A1* |
| 4.08E-45 | 1.461824836 | 0.609 | 0.102 | 9.75E-41 | 3 | *COL4A1* |
| 1.89E-36 | 1.392768516 | 0.565 | 0.107 | 4.53E-32 | 3 | *COL4A2* |
| 0 | 2.85637874 | 0.883 | 0.194 | 0 | 4 | *KRT8* |
| 0 | 2.423797505 | 0.868 | 0.198 | 0 | 4 | *KRT18* |
| 0 | 2.992302729 | 0.709 | 0.168 | 0 | 4 | *KRT15* |
| 0 | 3.691371678 | 0.903 | 0.489 | 0 | 4 | *KRT17* |
| 0 | 3.242043473 | 0.829 | 0.214 | 0 | 4 | *KRT19* |
| 0 | 2.475682638 | 0.729 | 0.185 | 0 | 4 | *KRT7* |
| 5.63E-90 | 1.210138731 | 0.208 | 0.057 | 1.34E-85 | 4 | *AGR2* |
| 0 | 2.880428926 | 0.809 | 0.3 | 0 | 4 | *CLDN4* |
| 0 | 2.856162553 | 0.873 | 0.195 | 0 | 5 | *VIM* |
| 0 | 3.454071435 | 0.938 | 0.291 | 0 | 6 | *IGFBP7* |
| 0 | 2.499927912 | 0.593 | 0.007 | 0 | 6 | *C7* |
| 0 | 2.181697356 | 0.884 | 0.189 | 0 | 6 | *VIM* |
| 0 | 5.217375937 | 0.618 | 0.033 | 0 | 6 | *PTGDS* |
| 4.82E-41 | 0.564181095 | 0.238 | 0.083 | 1.15E-36 | 6 | *MT1A* |
| 0 | 1.642268027 | 0.425 | 0.019 | 0 | 6 | *COL1A2* |
| 0 | 1.641305754 | 0.538 | 0.038 | 0 | 6 | *BGN* |
| 0 | 0.902969952 | 0.241 | 0.01 | 0 | 6 | *THY1* |
| 8.08E-188 | 0.759601363 | 0.203 | 0.019 | 1.93E-183 | 6 | *PDGFRB* |
| 9.60E-232 | 0.971135458 | 0.205 | 0.015 | 2.29E-227 | 6 | *COL3A1* |
| 4.74E-264 | 1.232920741 | 0.523 | 0.091 | 1.13E-259 | 6 | *COL4A1* |
| 1.66E-165 | 0.992812231 | 0.444 | 0.099 | 3.97E-161 | 6 | *COL4A2* |
| 1.95E-135 | 0.939593724 | 0.407 | 0.101 | 4.65E-131 | 6 | *COL18A1* |
| 0 | 2.725719016 | 0.637 | 0.005 | 0 | 7 | *CLDN5* |
| 0 | 3.571423507 | 0.56 | 0.005 | 0 | 7 | *SELE* |
| 0 | 2.353326014 | 0.674 | 0.005 | 0 | 7 | *VWF* |
| 0 | 1.681016412 | 0.381 | 0.015 | 0 | 7 | *ENG* |
| 0 | 1.482459275 | 0.891 | 0.28 | 0 | 7 | *IGFBP7* |
| 0 | 4.15315451 | 0.929 | 0.016 | 0 | 7 | *IFI27* |
| 0 | 1.488290193 | 0.45 | 0.002 | 0 | 7 | *EMCN* |
| 0 | 2.187251915 | 0.662 | 0.034 | 0 | 7 | *CD200* |
| 0 | 2.186447648 | 0.911 | 0.174 | 0 | 7 | *VIM* |
| 3.70E-182 | 0.614082245 | 0.238 | 0.04 | 8.83E-178 | 7 | *NRP1* |
| 0 | 2.058822244 | 0.359 | 0.023 | 0 | 7 | *ANGPT2* |
| 0 | 1.280317604 | 0.511 | 0.083 | 0 | 7 | *COL4A1* |
| 3.05E-182 | 0.743216789 | 0.38 | 0.095 | 7.28E-178 | 7 | *COL4A2* |
| **Normal_TZ** | | | | | | |
| 1.85E-140 | 0.372557006 | 0.203 | 0.032 | 4.39E-136 | 1 | *ENG* |
| 0 | 3.079806841 | 0.957 | 0.359 | 0 | 1 | *IGFBP7* |
| 0 | 2.036375365 | 0.36 | 0.011 | 0 | 1 | *C7* |
| 0 | 2.391628676 | 0.96 | 0.156 | 0 | 1 | *VIM* |
| 0 | 5.606956927 | 0.774 | 0.04 | 0 | 1 | *PTGDS* |
| 0 | 1.319309839 | 0.427 | 0.065 | 0 | 1 | *MT1A* |
| 0 | 1.383343552 | 0.369 | 0.015 | 0 | 1 | *COL1A2* |
| 0 | 1.657081051 | 0.565 | 0.029 | 0 | 1 | *BGN* |
| 1.49E-261 | 0.531823496 | 0.158 | 0.009 | 3.55E-257 | 1 | *THY1* |
| 1.88E-251 | 0.60431018 | 0.212 | 0.019 | 4.48E-247 | 1 | *PDGFRB* |
| 2.56E-301 | 0.691588762 | 0.18 | 0.01 | 6.09E-297 | 1 | *COL3A1* |
| 0 | 1.059400641 | 0.508 | 0.088 | 0 | 1 | *COL4A1* |
| 1.97E-258 | 0.830969244 | 0.457 | 0.088 | 4.68E-254 | 1 | *COL4A2* |
| 0 | 1.010963011 | 0.443 | 0.065 | 0 | 1 | *COL18A1* |
| 0 | 2.864513126 | 0.671 | 0.005 | 0 | 2 | *CLDN5* |
| 0 | 3.817123952 | 0.629 | 0.006 | 0 | 2 | *SELE* |
| 0 | 1.86887866 | 0.578 | 0.003 | 0 | 2 | *VWF* |
| 0 | 1.299740773 | 0.343 | 0.022 | 0 | 2 | *ENG* |
| 0 | 1.71955878 | 0.916 | 0.356 | 0 | 2 | *IGFBP7* |
| 0 | 4.198996762 | 0.935 | 0.04 | 0 | 2 | *IFI27* |
| 0 | 1.354137877 | 0.448 | 0.002 | 0 | 2 | *EMCN* |
| 0 | 2.48703736 | 0.762 | 0.046 | 0 | 2 | *CD200* |
| 0 | 2.263454491 | 0.937 | 0.151 | 0 | 2 | *VIM* |
| 6.20E-302 | 0.697304556 | 0.293 | 0.035 | 1.48E-297 | 2 | *NRP1* |
| 0 | 2.225982365 | 0.332 | 0.022 | 0 | 2 | *ANGPT2* |
| 0 | 1.16525775 | 0.53 | 0.083 | 0 | 2 | *COL4A1* |
| 1.09E-197 | 0.721588322 | 0.389 | 0.088 | 2.58E-193 | 2 | *COL4A2* |
| 0 | 2.931764792 | 0.941 | 0.18 | 0 | 3 | *VIM* |
| 0 | 2.798632664 | 0.863 | 0.169 | 0 | 4 | *KRT8* |
| 0 | 2.495434895 | 0.866 | 0.194 | 0 | 4 | *KRT18* |
| 0 | 3.138525566 | 0.718 | 0.111 | 0 | 4 | *KRT15* |
| 0 | 4.77674438 | 0.937 | 0.397 | 0 | 4 | *KRT17* |
| 0 | 3.784433961 | 0.874 | 0.21 | 0 | 4 | *KRT19* |
| 0 | 2.866856017 | 0.755 | 0.139 | 0 | 4 | *KRT7* |
| 1.76E-179 | 1.352512536 | 0.299 | 0.051 | 4.19E-175 | 4 | *AGR2* |
| 0 | 3.311132517 | 0.82 | 0.274 | 0 | 4 | *CLDN4* |
| 0 | 3.169625844 | 0.969 | 0.364 | 0 | 5 | *IGFBP7* |
| 2.84E-35 | 0.457582341 | 0.102 | 0.024 | 6.76E-31 | 5 | *C7* |
| 0 | 2.630799526 | 0.888 | 0.167 | 0 | 5 | *VIM* |
| 0 | 3.596325237 | 0.65 | 0.01 | 0 | 5 | *GJA4* |
| 0 | 4.673559615 | 0.629 | 0.013 | 0 | 5 | *RGS5* |
| 0 | 4.486953543 | 0.724 | 0.057 | 0 | 5 | *MT1A* |
| 0 | 1.018807204 | 0.312 | 0.021 | 0 | 5 | *COL1A2* |
| 0 | 3.355184987 | 0.618 | 0.014 | 0 | 5 | *MYH11* |
| 0 | 3.688523103 | 0.36 | 0.008 | 0 | 5 | *ACTG2* |
| 0 | 2.322834495 | 0.603 | 0.033 | 0 | 5 | *BGN* |
| 1.03E-280 | 0.796898528 | 0.182 | 0.009 | 2.44E-276 | 5 | *THY1* |
| 0 | 1.655811653 | 0.422 | 0.013 | 0 | 5 | *PDGFRB* |
| 5.22E-24 | 0.314091784 | 0.129 | 0.046 | 1.24E-19 | 5 | *NRP1* |
| 1.76E-114 | 0.966284541 | 0.201 | 0.032 | 4.19E-110 | 5 | *ANGPT2* |
| 9.38E-307 | 0.794092866 | 0.203 | 0.011 | 2.23E-302 | 5 | *COL3A1* |
| 0 | 1.999255492 | 0.634 | 0.087 | 0 | 5 | *COL4A1* |
| 0 | 1.554857631 | 0.542 | 0.088 | 0 | 5 | *COL4A2* |
| 1.80E-198 | 1.195296946 | 0.379 | 0.071 | 4.28E-194 | 5 | *COL18A1* |
| 1.28E-19 | 2.366031248 | 0.9 | 0.385 | 3.04E-15 | 6 | *IGFBP7* |
| 3.29E-46 | 3.939286803 | 0.95 | 0.191 | 7.82E-42 | 6 | *VIM* |
| 4.22E-42 | 2.182070423 | 0.4 | 0.031 | 1.00E-37 | 6 | *COL1A2* |
| 1.81E-23 | 1.229720234 | 0.225 | 0.017 | 4.31E-19 | 6 | *COL3A1* |
| **AN** | | | | | | |
| 2.84E-17 | 1.272330691 | 0.218 | 0.062 | 4.87E-13 | 1 | *CLDN5* |
| 4.95E-267 | 4.46551324 | 0.411 | 0.003 | 8.47E-263 | 1 | *SELE* |
| 0 | 4.760608898 | 0.848 | 0.009 | 0 | 1 | *VWF* |
| 5.35E-223 | 3.008674048 | 0.563 | 0.027 | 9.16E-219 | 1 | *ENG* |
| 8.15E-190 | 2.946100793 | 0.858 | 0.105 | 1.40E-185 | 1 | *IGFBP7* |
| 6.20E-290 | 3.12029249 | 0.574 | 0.014 | 1.06E-285 | 1 | *IFI27* |
| 8.65E-259 | 2.88281725 | 0.452 | 0.007 | 1.48E-254 | 1 | *EMCN* |
| 4.28E-42 | 1.214114382 | 0.112 | 0.005 | 7.32E-38 | 1 | *CD200* |
| 1.96E-28 | 0.678634411 | 0.173 | 0.025 | 3.36E-24 | 1 | *C7* |
| 2.52E-102 | 2.339610021 | 0.934 | 0.307 | 4.32E-98 | 1 | *VIM* |
| 3.24E-08 | 0.468997006 | 0.127 | 0.041 | 0.000555109 | 1 | *RGS5* |
| 4.38E-80 | 2.303999183 | 0.411 | 0.054 | 7.49E-76 | 1 | *NRP1* |
| 1.06E-27 | 1.356676594 | 0.102 | 0.009 | 1.81E-23 | 1 | *ANGPT2* |
| 1.73E-122 | 2.103204227 | 0.376 | 0.023 | 2.96E-118 | 1 | *COL4A1* |
| 1.07E-86 | 1.890467052 | 0.35 | 0.033 | 1.83E-82 | 1 | *COL4A2* |
| 1.11E-08 | 0.615101277 | 0.188 | 0.073 | 0.000190717 | 1 | *COL18A1* |
| 1.88E-87 | 1.676905478 | 0.285 | 0.011 | 3.22E-83 | 2 | *KRT8* |
| 2.97E-145 | 2.249735356 | 0.433 | 0.02 | 5.08E-141 | 2 | *KRT18* |
| 1.19E-61 | 2.939841328 | 0.219 | 0.014 | 2.03E-57 | 2 | *KRT15* |
| 2.51E-13 | 0.451180135 | 0.043 | 0.001 | 4.29E-09 | 2 | *KRT17* |
| 1.17E-88 | 2.567109837 | 0.295 | 0.016 | 2.00E-84 | 2 | *KRT19* |
| 1.94E-27 | 1.117514464 | 0.097 | 0.004 | 3.32E-23 | 2 | *KRT7* |
| 4.07E-86 | 2.129652173 | 0.275 | 0.008 | 6.97E-82 | 2 | *AGR2* |
| 5.26E-181 | 2.984829048 | 0.508 | 0.021 | 9.01E-177 | 2 | *CLDN4* |
| 4.69E-80 | 3.861755717 | 0.857 | 0.063 | 8.04E-76 | 3 | *CLDN5* |
| 2.54E-10 | 0.71238947 | 0.314 | 0.054 | 4.34E-06 | 3 | *VWF* |
| 2.21E-21 | 1.994490961 | 0.429 | 0.053 | 3.78E-17 | 3 | *ENG* |
| 5.64E-18 | 1.485189041 | 0.686 | 0.142 | 9.65E-14 | 3 | *IGFBP7* |
| 4.98E-111 | 3.515350229 | 0.829 | 0.038 | 8.53E-107 | 3 | *IFI27* |
| 5.39E-66 | 2.51190222 | 0.543 | 0.027 | 9.22E-62 | 3 | *EMCN* |
| 2.32E-23 | 2.183254925 | 1 | 0.336 | 3.97E-19 | 3 | *VIM* |
| 8.06E-46 | 2.013478519 | 0.314 | 0.013 | 1.38E-41 | 3 | *GJA4* |
| 4.69E-20 | 1.793784875 | 0.371 | 0.042 | 8.03E-16 | 3 | *RGS5* |
| 6.37E-10 | 1.267441391 | 0.257 | 0.041 | 1.09E-05 | 3 | *COL4A1* |
| 3.76E-07 | 1.12316079 | 0.314 | 0.078 | 0.006446207 | 3 | *COL18A1* |
| 8.34E-33 | 0.909125424 | 0.699 | 0.315 | 1.43E-28 | 4 | *VIM* |
| 5.87E-17 | 0.762347756 | 0.207 | 0.064 | 1.01E-12 | 4 | *NRP1* |
| 1.06E-149 | 3.495598522 | 0.963 | 0.121 | 1.81E-145 | 5 | *IGFBP7* |
| 3.21E-45 | 2.023378528 | 0.872 | 0.326 | 5.49E-41 | 5 | *VIM* |
| 5.03E-149 | 2.586932754 | 0.321 | 0.006 | 8.61E-145 | 5 | *GJA4* |
| 0 | 5.765779496 | 0.872 | 0.019 | 0 | 5 | *RGS5* |
| 2.34E-38 | 1.45703329 | 0.138 | 0.007 | 4.00E-34 | 5 | *MT1A* |
| 4.93E-34 | 1.268286874 | 0.294 | 0.039 | 8.45E-30 | 5 | *COL1A2* |
| 0 | 7.257764797 | 0.954 | 0.021 | 0 | 5 | *MYH11* |
| 7.23E-76 | 1.781902225 | 0.202 | 0.006 | 1.24E-71 | 5 | *ACTG2* |
| 1.43E-111 | 2.370581555 | 0.394 | 0.019 | 2.46E-107 | 5 | *BGN* |
| 2.72E-23 | 0.951882152 | 0.092 | 0.005 | 4.65E-19 | 5 | *THY1* |
| 3.02E-125 | 2.41825883 | 0.385 | 0.015 | 5.17E-121 | 5 | *PDGFRB* |
| 3.27E-29 | 0.928872042 | 0.138 | 0.01 | 5.60E-25 | 5 | *ANGPT2* |
| 4.03E-55 | 2.220249021 | 0.303 | 0.026 | 6.89E-51 | 5 | *COL3A1* |
| 3.53E-31 | 1.50484877 | 0.266 | 0.036 | 6.04E-27 | 5 | *COL4A1* |
| 7.21E-57 | 2.131529195 | 0.376 | 0.04 | 1.23E-52 | 5 | *COL4A2* |
| 4.64E-53 | 2.423620732 | 0.459 | 0.068 | 7.94E-49 | 5 | *COL18A1* |
| 2.28E-36 | 0.594638542 | 0.617 | 0.297 | 3.91E-32 | 6 | *VIM* |
| 5.90E-108 | 2.676418767 | 0.812 | 0.122 | 1.01E-103 | 7 | *IGFBP7* |
| 6.39E-245 | 4.497803646 | 0.547 | 0.013 | 1.09E-240 | 7 | *C7* |
| 5.32E-39 | 1.455026053 | 0.844 | 0.324 | 9.11E-35 | 7 | *VIM* |
| 2.30E-229 | 3.159505527 | 0.352 | 0.002 | 3.93E-225 | 7 | *PTGDS* |
| 0 | 5.225418509 | 0.883 | 0.015 | 0 | 7 | *COL1A2* |
| 1.15E-24 | 0.344263599 | 0.25 | 0.043 | 1.97E-20 | 7 | *MYH11* |
| 8.42E-33 | 1.517057299 | 0.125 | 0.008 | 1.44E-28 | 7 | *ACTG2* |
| 6.43E-130 | 2.742951186 | 0.391 | 0.017 | 1.10E-125 | 7 | *BGN* |
| 6.28E-39 | 0.971338968 | 0.109 | 0.004 | 1.08E-34 | 7 | *THY1* |
| 1.14E-83 | 2.110703519 | 0.297 | 0.016 | 1.96E-79 | 7 | *PDGFRB* |
| 7.23E-175 | 3.672492274 | 0.477 | 0.017 | 1.24E-170 | 7 | *COL3A1* |
| 6.69E-20 | 1.650962409 | 0.203 | 0.037 | 1.15E-15 | 7 | *COL4A1* |
| 7.34E-44 | 2.028578394 | 0.312 | 0.041 | 1.26E-39 | 7 | *COL4A2* |
| 2.41E-31 | 1.90022736 | 0.344 | 0.07 | 4.12E-27 | 7 | *COL18A1* |
| 1.57E-27 | 2.556239853 | 0.976 | 0.335 | 2.69E-23 | 8 | *VIM* |
| **Benign** | | | | | | |
| 2.92E-21 | 5.137912442 | 1 | 0.2 | 6.19E-17 | 1 | *IGFBP7* |
| 9.49E-12 | 3.422168451 | 1 | 0.567 | 2.01E-07 | 1 | *VIM* |
| 0 | 2.479977218 | 0.8 | 0.001 | 0 | 1 | *GJA4* |
| 2.27E-286 | 5.025040977 | 0.933 | 0.007 | 4.80E-282 | 1 | *RGS5* |
| 1.92E-12 | 3.418459715 | 0.4 | 0.042 | 4.07E-08 | 1 | *MT1A* |
| 0 | 2.263223295 | 0.867 | 0.002 | 0 | 1 | *COL1A2* |
| 4.18E-264 | 4.737580072 | 1 | 0.01 | 8.85E-260 | 1 | *MYH11* |
| 0 | 3.688102848 | 0.8 | 0.002 | 0 | 1 | *ACTG2* |
| 1.55E-276 | 3.46471974 | 1 | 0.009 | 3.29E-272 | 1 | *BGN* |
| 5.02E-34 | 1.602910438 | 0.267 | 0.006 | 1.06E-29 | 1 | *THY1* |
| 0 | 1.988007947 | 0.667 | 0.002 | 0 | 1 | *PDGFRB* |
| 2.58E-27 | 0.599974411 | 0.4 | 0.018 | 5.47E-23 | 1 | *ANGPT2* |
| 7.57E-301 | 2.05500619 | 0.667 | 0.003 | 1.60E-296 | 1 | *COL3A1* |
| 5.73E-87 | 1.521789475 | 0.6 | 0.012 | 1.21E-82 | 1 | *COL4A1* |
| 9.30E-57 | 1.529963003 | 0.667 | 0.024 | 1.97E-52 | 1 | *COL4A2* |
| 1.25E-18 | 1.506907365 | 0.933 | 0.159 | 2.64E-14 | 1 | *COL18A1* |
| 6.66E-166 | 2.73945374 | 0.634 | 0.04 | 1.41E-161 | 2 | *CLDN5* |
| 0 | 3.862657557 | 0.762 | 0.006 | 0 | 2 | *SELE* |
| 0 | 3.217296743 | 0.95 | 0.009 | 0 | 2 | *VWF* |
| 4.36E-282 | 2.774862248 | 0.911 | 0.05 | 9.22E-278 | 2 | *ENG* |
| 4.69E-131 | 4.975850626 | 1 | 0.187 | 9.92E-127 | 2 | *IGFBP7* |
| 0 | 4.296695538 | 1 | 0.031 | 0 | 2 | *IFI27* |
| 0 | 2.017327886 | 0.792 | 0.001 | 0 | 2 | *EMCN* |
| 6.85E-102 | 1.215067501 | 0.574 | 0.055 | 1.45E-97 | 2 | *CD200* |
| 0 | 0.943179606 | 0.366 | 0.001 | 0 | 2 | *C7* |
| 8.24E-68 | 3.27185231 | 1 | 0.56 | 1.74E-63 | 2 | *VIM* |
| 0 | 2.199120176 | 0.505 | 0.007 | 0 | 2 | *PTGDS* |
| 3.02E-120 | 0.462767008 | 0.198 | 0.003 | 6.40E-116 | 2 | *THY1* |
| 2.14E-93 | 1.122253118 | 0.564 | 0.059 | 4.52E-89 | 2 | *NRP1* |
| 3.66E-60 | 0.562162673 | 0.238 | 0.015 | 7.75E-56 | 2 | *ANGPT2* |
| 8.33E-288 | 0.880723313 | 0.426 | 0.005 | 1.76E-283 | 2 | *COL4A1* |
| 6.77E-178 | 1.040027579 | 0.465 | 0.017 | 1.43E-173 | 2 | *COL4A2* |
| 2.75E-10 | 0.370466388 | 0.386 | 0.157 | 5.82E-06 | 2 | *COL18A1* |
| 2.02E-36 | 2.215786229 | 0.764 | 0.198 | 4.28E-32 | 3 | *KRT8* |
| 3.81E-29 | 1.913870525 | 0.761 | 0.319 | 8.06E-25 | 3 | *KRT18* |
| 3.43E-10 | 2.534907362 | 0.386 | 0.121 | 7.26E-06 | 3 | *KRT15* |
| 1.65E-15 | 2.368250895 | 0.44 | 0.069 | 3.48E-11 | 3 | *KRT19* |
| 1.37E-06 | 1.286379692 | 0.243 | 0.052 | 0.029004263 | 3 | *AGR2* |
| 1.07E-19 | 2.057074167 | 0.535 | 0.103 | 2.27E-15 | 3 | *CLDN4* |
| **BPH_GN** | | | | | | |
| 2.18E-105 | 0.787825137 | 0.847 | 0.433 | 5.62E-101 | 1 | *KRT8* |
| 5.96E-172 | 1.424802192 | 0.692 | 0.272 | 1.54E-167 | 1 | *KRT15* |
| 0 | 2.717182232 | 0.63 | 0.021 | 0 | 4 | *CLDN5* |
| 0 | 5.023403027 | 0.687 | 0.028 | 0 | 4 | *SELE* |
| 0 | 3.074516076 | 0.839 | 0.018 | 0 | 4 | *VWF* |
| 0 | 1.838039414 | 0.652 | 0.061 | 0 | 4 | *ENG* |
| 0 | 2.03949656 | 0.981 | 0.393 | 0 | 4 | *IGFBP7* |
| 0 | 3.890987277 | 0.959 | 0.04 | 0 | 4 | *IFI27* |
| 0 | 2.064543785 | 0.653 | 0.012 | 0 | 4 | *EMCN* |
| 0 | 2.241366471 | 0.711 | 0.033 | 0 | 4 | *CD200* |
| 0 | 1.263795565 | 0.989 | 0.616 | 0 | 4 | *VIM* |
| 0 | 1.289528993 | 0.512 | 0.087 | 0 | 4 | *NRP1* |
| 0 | 2.828928908 | 0.71 | 0.066 | 0 | 4 | *ANGPT2* |
| 0 | 1.420305438 | 0.731 | 0.184 | 0 | 4 | *COL4A1* |
| 0 | 1.019973763 | 0.605 | 0.165 | 0 | 4 | *COL4A2* |
| 0 | 2.81406592 | 0.993 | 0.404 | 0 | 5 | *IGFBP7* |
| 0 | 0.84297749 | 0.98 | 0.624 | 0 | 5 | *VIM* |
| 0 | 3.299421014 | 0.779 | 0.022 | 0 | 5 | *GJA4* |
| 0 | 4.824759022 | 0.845 | 0.025 | 0 | 5 | *RGS5* |
| 0 | 5.032434086 | 0.91 | 0.121 | 0 | 5 | *MT1A* |
| 0 | 1.602602079 | 0.573 | 0.084 | 0 | 5 | *COL1A2* |
| 0 | 2.743707854 | 0.653 | 0.029 | 0 | 5 | *MYH11* |
| 0 | 1.337900818 | 0.262 | 0.012 | 0 | 5 | *ACTG2* |
| 0 | 2.246432324 | 0.644 | 0.05 | 0 | 5 | *BGN* |
| 0 | 1.444898245 | 0.383 | 0.044 | 0 | 5 | *THY1* |
| 0 | 1.624006293 | 0.51 | 0.04 | 0 | 5 | *PDGFRB* |
| 1.66E-59 | 0.349373699 | 0.227 | 0.123 | 4.28E-55 | 5 | *NRP1* |
| 0 | 1.873591187 | 0.454 | 0.047 | 0 | 5 | *COL3A1* |
| 0 | 2.232776409 | 0.803 | 0.187 | 0 | 5 | *COL4A1* |
| 0 | 1.948029637 | 0.723 | 0.162 | 0 | 5 | *COL4A2* |
| 0 | 1.426694814 | 0.522 | 0.131 | 0 | 5 | *COL18A1* |
| 0 | 2.558724709 | 0.986 | 0.644 | 0 | 6 | *VIM* |
| 0 | 0.99801265 | 0.964 | 0.411 | 0 | 7 | *IGFBP7* |
| 0 | 3.410248857 | 0.846 | 0.03 | 0 | 7 | *C7* |
| 0 | 0.65781187 | 0.973 | 0.627 | 0 | 7 | *VIM* |
| 0 | 4.383264159 | 0.568 | 0.037 | 0 | 7 | *PTGDS* |
| 0 | 2.701051039 | 0.848 | 0.065 | 0 | 7 | *COL1A2* |
| 0 | 1.093938031 | 0.411 | 0.075 | 0 | 7 | *BGN* |
| 1.50E-243 | 0.503610066 | 0.24 | 0.059 | 3.85E-239 | 7 | *THY1* |
| 0 | 0.735271899 | 0.354 | 0.057 | 0 | 7 | *PDGFRB* |
| 0 | 1.302818377 | 0.449 | 0.05 | 0 | 7 | *COL3A1* |
| 1.75E-132 | 0.359196965 | 0.415 | 0.194 | 4.51E-128 | 7 | *COL4A2* |
| 9.25E-168 | 0.465494944 | 0.362 | 0.148 | 2.38E-163 | 7 | *COL18A1* |
| 0 | 3.013035123 | 0.86 | 0.077 | 0 | 9 | *CLDN5* |
| 1.29E-225 | 1.456884143 | 0.642 | 0.098 | 3.33E-221 | 9 | *VWF* |
| 4.00E-200 | 1.395266703 | 0.642 | 0.118 | 1.03E-195 | 9 | *ENG* |
| 5.54E-106 | 1.312984309 | 0.971 | 0.45 | 1.43E-101 | 9 | *IGFBP7* |
| 0 | 2.792136657 | 0.948 | 0.128 | 0 | 9 | *IFI27* |
| 0 | 1.472130194 | 0.636 | 0.074 | 0 | 9 | *EMCN* |
| 4.45E-30 | 0.476689546 | 0.295 | 0.102 | 1.15E-25 | 9 | *CD200* |
| 2.03E-121 | 1.539216555 | 0.994 | 0.651 | 5.23E-117 | 9 | *VIM* |
| 2.66E-90 | 0.848640712 | 0.404 | 0.085 | 6.85E-86 | 9 | *GJA4* |
| 3.15E-26 | 0.500218116 | 0.324 | 0.13 | 8.13E-22 | 9 | *NRP1* |
| 0 | 3.434787432 | 0.9 | 0.126 | 0 | 9 | *ANGPT2* |
| 1.02E-36 | 0.552898556 | 0.547 | 0.238 | 2.63E-32 | 9 | *COL4A1* |
| 3.88E-11 | 0.264205519 | 0.367 | 0.21 | 1.00E-06 | 9 | *COL4A2* |
| 4.77E-18 | 0.434592968 | 0.332 | 0.163 | 1.23E-13 | 9 | *COL18A1* |
| 0 | 2.654324393 | 0.931 | 0.137 | 0 | 10 | *KRT8* |
| 0 | 2.526875167 | 0.913 | 0.204 | 0 | 10 | *KRT18* |
| 0 | 2.257961046 | 0.592 | 0.087 | 0 | 10 | *KRT15* |
| 0 | 3.571806503 | 0.689 | 0.184 | 0 | 10 | *KRT17* |
| 0 | 2.535155012 | 0.312 | 0.076 | 0 | 10 | *KRT19* |
| 0 | 0.97010353 | 0.252 | 0.017 | 0 | 10 | *KRT7* |
| 0 | 1.82295454 | 0.268 | 0.024 | 0 | 10 | *AGR2* |
| 0 | 3.137251764 | 0.553 | 0.1 | 0 | 10 | *CLDN4* |
| **BPH_SN** | | | | | | |
| 6.74E-212 | 0.553524997 | 0.265 | 0.058 | 1.75E-207 | 2 | *KRT19* |
| 0 | 1.980330871 | 0.991 | 0.857 | 0 | 2 | *VIM* |
| 0 | 3.062689066 | 0.903 | 0.043 | 0 | 3 | *KRT8* |
| 0 | 2.801160891 | 0.871 | 0.079 | 0 | 3 | *KRT18* |
| 0 | 2.029930571 | 0.376 | 0.011 | 0 | 3 | *KRT15* |
| 0 | 4.228442656 | 0.709 | 0.078 | 0 | 3 | *KRT17* |
| 0 | 2.97913994 | 0.29 | 0.053 | 0 | 3 | *KRT19* |
| 0 | 1.559256749 | 0.235 | 0.009 | 0 | 3 | *KRT7* |
| 0 | 1.260866001 | 0.175 | 0.005 | 0 | 3 | *AGR2* |
| 0 | 2.639038805 | 0.344 | 0.027 | 0 | 3 | *CLDN4* |
| 1.50E-11 | 0.524879355 | 0.154 | 0.094 | 3.89E-07 | 4 | *KRT8* |
| 2.83E-10 | 0.514509032 | 0.184 | 0.126 | 7.35E-06 | 4 | *KRT18* |
| 7.05E-09 | 0.376183555 | 0.066 | 0.033 | 0.000183451 | 4 | *KRT15* |
| 0 | 1.373427403 | 0.515 | 0.104 | 0 | 4 | *KRT17* |
| 2.06E-16 | 0.275823583 | 0.13 | 0.066 | 5.37E-12 | 4 | *KRT19* |
| 1.77E-40 | 0.511044684 | 0.133 | 0.043 | 4.62E-36 | 4 | *CLDN4* |
| 2.08E-29 | 0.656768396 | 0.165 | 0.072 | 5.41E-25 | 4 | *CLDN5* |
| 0 | 1.175045525 | 0.76 | 0.15 | 0 | 4 | *SELE* |
| 1.73E-138 | 1.329669047 | 0.36 | 0.109 | 4.51E-134 | 4 | *VWF* |
| 7.85E-204 | 0.867159617 | 0.977 | 0.643 | 2.04E-199 | 4 | *IGFBP7* |
| 6.84E-281 | 1.366550311 | 0.626 | 0.17 | 1.78E-276 | 4 | *IFI27* |
| 9.88E-21 | 0.761765082 | 0.155 | 0.079 | 2.57E-16 | 4 | *EMCN* |
| 1.26E-12 | 0.593663789 | 0.151 | 0.09 | 3.28E-08 | 4 | *CD200* |
| 0 | 1.873536497 | 0.785 | 0.202 | 0 | 5 | *C7* |
| 1.56E-297 | 1.838203853 | 0.654 | 0.301 | 4.05E-293 | 5 | *PTGDS* |
| 0 | 1.755877253 | 0.812 | 0.229 | 0 | 5 | *COL1A2* |
| 5.49E-176 | 0.527188568 | 0.301 | 0.093 | 1.43E-171 | 5 | *PDGFRB* |
| 0 | 0.66727787 | 0.395 | 0.097 | 0 | 5 | *COL3A1* |
| 0 | 1.833742671 | 0.991 | 0.631 | 0 | 6 | *IGFBP7* |
| 0 | 2.606952449 | 0.742 | 0.065 | 0 | 6 | *GJA4* |
| 0 | 4.657762924 | 0.948 | 0.088 | 0 | 6 | *RGS5* |
| 0 | 2.555220698 | 0.924 | 0.428 | 0 | 6 | *MT1A* |
| 7.90E-165 | 1.003331512 | 0.529 | 0.25 | 2.06E-160 | 6 | *COL1A2* |
| 0 | 1.649493809 | 0.679 | 0.172 | 0 | 6 | *MYH11* |
| 1.09E-113 | 0.580356015 | 0.322 | 0.134 | 2.84E-109 | 6 | *ACTG2* |
| 0 | 2.350945076 | 0.696 | 0.099 | 0 | 6 | *BGN* |
| 0 | 1.059269007 | 0.279 | 0.044 | 0 | 6 | *THY1* |
| 0 | 1.250431448 | 0.428 | 0.085 | 0 | 6 | *PDGFRB* |
| 1.08E-39 | 0.354468103 | 0.201 | 0.107 | 2.82E-35 | 6 | *NRP1* |
| 2.50E-263 | 0.489561097 | 0.419 | 0.124 | 6.50E-259 | 6 | *ANGPT2* |
| 0 | 1.900025457 | 0.428 | 0.095 | 0 | 6 | *COL3A1* |
| 0 | 1.875231521 | 0.777 | 0.255 | 0 | 6 | *COL4A1* |
| 0 | 1.616595183 | 0.686 | 0.22 | 0 | 6 | *COL4A2* |
| 0 | 1.311771011 | 0.486 | 0.134 | 0 | 6 | *COL18A1* |
| 0 | 2.262481291 | 0.786 | 0.13 | 0 | 7 | *C7* |
| 6.03E-213 | 0.265671766 | 0.979 | 0.84 | 1.57E-208 | 7 | *VIM* |
| 0 | 3.261482745 | 0.705 | 0.247 | 0 | 7 | *PTGDS* |
| 0 | 0.770884598 | 0.79 | 0.393 | 0 | 7 | *MT1A* |
| 0 | 1.436831005 | 0.713 | 0.178 | 0 | 7 | *COL1A2* |
| 1.24E-103 | 0.262732524 | 0.194 | 0.09 | 3.24E-99 | 7 | *PDGFRB* |
| 4.95E-208 | 0.311252834 | 0.488 | 0.248 | 1.29E-203 | 7 | *COL4A1* |
| 3.11E-167 | 0.348556542 | 0.414 | 0.217 | 8.10E-163 | 7 | *COL4A2* |
| 1.53E-81 | 0.396744639 | 0.292 | 0.119 | 3.97E-77 | 9 | *KRT18* |
| 0 | 2.072456257 | 0.997 | 0.636 | 0 | 9 | *IGFBP7* |
| 0 | 2.29043006 | 0.624 | 0.081 | 0 | 9 | *GJA4* |
| 0 | 0.878224384 | 0.58 | 0.121 | 0 | 9 | *RGS5* |
| 0 | 3.086610095 | 0.936 | 0.435 | 0 | 9 | *MT1A* |
| 9.29E-108 | 0.536304748 | 0.531 | 0.254 | 2.42E-103 | 9 | *COL1A2* |
| 0 | 2.88007799 | 0.865 | 0.17 | 0 | 9 | *MYH11* |
| 0 | 1.665523889 | 0.467 | 0.129 | 0 | 9 | *ACTG2* |
| 0 | 1.058032286 | 0.472 | 0.119 | 0 | 9 | *BGN* |
| 2.25E-196 | 0.709249389 | 0.233 | 0.05 | 5.84E-192 | 9 | *THY1* |
| 7.62E-213 | 0.982288241 | 0.341 | 0.095 | 1.98E-208 | 9 | *PDGFRB* |
| 4.46E-93 | 0.671827879 | 0.279 | 0.108 | 1.16E-88 | 9 | *COL3A1* |
| 0 | 1.265900383 | 0.699 | 0.266 | 0 | 9 | *COL4A1* |
| 5.45E-262 | 1.052690749 | 0.6 | 0.232 | 1.42E-257 | 9 | *COL4A2* |
| 1.85E-51 | 0.48879676 | 0.286 | 0.15 | 4.83E-47 | 9 | *COL18A1* |
| 0 | 2.23730074 | 0.495 | 0.018 | 0 | 10 | *CLDN5* |
| 0 | 4.49140304 | 0.745 | 0.091 | 0 | 10 | *SELE* |
| 0 | 2.691709763 | 0.768 | 0.029 | 0 | 10 | *VWF* |
| 0 | 1.768382764 | 0.574 | 0.079 | 0 | 10 | *ENG* |
| 0 | 1.386626561 | 0.977 | 0.61 | 0 | 10 | *IGFBP7* |
| 0 | 3.884593461 | 0.943 | 0.081 | 0 | 10 | *IFI27* |
| 0 | 1.913149462 | 0.592 | 0.012 | 0 | 10 | *EMCN* |
| 0 | 1.959010645 | 0.585 | 0.026 | 0 | 10 | *CD200* |
| 0 | 0.584968278 | 0.987 | 0.847 | 0 | 10 | *VIM* |
| 0 | 0.877267511 | 0.371 | 0.078 | 0 | 10 | *NRP1* |
| 0 | 2.961736015 | 0.665 | 0.073 | 0 | 10 | *ANGPT2* |
| 0 | 0.954041731 | 0.637 | 0.241 | 0 | 10 | *COL4A1* |
| 0 | 0.718829395 | 0.527 | 0.213 | 0 | 10 | *COL4A2* |
| **PCa** | | | | | | |
| 8.66E-39 | 1.096005464 | 0.581 | 0.291 | 2.41E-34 | 1 | *IGFBP7* |
| 3.49E-224 | 1.715944489 | 0.274 | 0.02 | 9.72E-220 | 1 | *C7* |
| 9.56E-32 | 1.022948568 | 0.642 | 0.412 | 2.66E-27 | 1 | *VIM* |
| 7.62E-113 | 3.355527854 | 0.246 | 0.032 | 2.12E-108 | 1 | *PTGDS* |
| 0 | 4.091409781 | 0.536 | 0.049 | 0 | 1 | *COL1A2* |
| 7.68E-22 | 0.616537609 | 0.092 | 0.02 | 2.14E-17 | 1 | *ACTG2* |
| 5.01E-306 | 2.685704228 | 0.62 | 0.081 | 1.39E-301 | 1 | *BGN* |
| 1.00E-120 | 1.261850415 | 0.358 | 0.058 | 2.80E-116 | 1 | *THY1* |
| 1.48E-84 | 0.809615667 | 0.321 | 0.061 | 4.13E-80 | 1 | *PDGFRB* |
| 5.62E-304 | 3.437299853 | 0.453 | 0.042 | 1.57E-299 | 1 | *COL3A1* |
| 1.22E-19 | 0.768393111 | 0.254 | 0.103 | 3.39E-15 | 1 | *COL4A1* |
| 1.48E-43 | 0.686549041 | 0.335 | 0.103 | 4.12E-39 | 1 | *COL4A2* |
| 2.74E-36 | 0.642775299 | 0.341 | 0.121 | 7.62E-32 | 1 | *COL18A1* |
| 0 | 1.85621078 | 0.631 | 0.166 | 0 | 2 | *KRT8* |
| 0 | 2.069211223 | 0.675 | 0.24 | 0 | 2 | *KRT18* |
| 0 | 1.103597252 | 0.345 | 0.108 | 0 | 2 | *KRT19* |
| 0 | 1.929386861 | 0.445 | 0.085 | 0 | 2 | *AGR2* |
| 0 | 1.544727796 | 0.544 | 0.169 | 0 | 2 | *CLDN4* |
| 0 | 2.673628361 | 0.512 | 0.028 | 0 | 3 | *CLDN5* |
| 0 | 2.033377139 | 0.281 | 0.011 | 0 | 3 | *SELE* |
| 0 | 3.220918054 | 0.803 | 0.044 | 0 | 3 | *VWF* |
| 0 | 2.888958397 | 0.816 | 0.037 | 0 | 3 | *ENG* |
| 0 | 2.70451337 | 0.967 | 0.207 | 0 | 3 | *IGFBP7* |
| 0 | 4.399283509 | 0.959 | 0.078 | 0 | 3 | *IFI27* |
| 0 | 2.738927553 | 0.771 | 0.012 | 0 | 3 | *EMCN* |
| 0 | 0.898773744 | 0.277 | 0.006 | 0 | 3 | *CD200* |
| 0 | 0.508603323 | 0.118 | 0.011 | 0 | 3 | *C7* |
| 0 | 1.92917101 | 0.96 | 0.344 | 0 | 3 | *VIM* |
| 0 | 0.586422651 | 0.148 | 0.019 | 0 | 3 | *PTGDS* |
| 8.04E-107 | 0.270060139 | 0.139 | 0.049 | 2.24E-102 | 3 | *GJA4* |
| 0 | 1.043936342 | 0.251 | 0.037 | 0 | 3 | *THY1* |
| 0 | 1.223645226 | 0.464 | 0.117 | 0 | 3 | *NRP1* |
| 0 | 1.689318546 | 0.362 | 0.05 | 0 | 3 | *ANGPT2* |
| 0 | 2.214357207 | 0.507 | 0.052 | 0 | 3 | *COL4A1* |
| 0 | 1.723641543 | 0.48 | 0.057 | 0 | 3 | *COL4A2* |
| 3.22E-292 | 0.50093343 | 0.307 | 0.099 | 8.96E-288 | 3 | *COL18A1* |
| 0 | 3.285379263 | 0.997 | 0.242 | 0 | 4 | *IGFBP7* |
| 1.17E-72 | 0.285417617 | 0.077 | 0.019 | 3.26E-68 | 4 | *C7* |
| 0 | 1.785172176 | 0.972 | 0.373 | 0 | 4 | *VIM* |
| 0 | 2.073535706 | 0.567 | 0.022 | 0 | 4 | *GJA4* |
| 0 | 5.252233988 | 0.962 | 0.063 | 0 | 4 | *RGS5* |
| 0 | 1.953973339 | 0.397 | 0.02 | 0 | 4 | *MT1A* |
| 0 | 1.986640327 | 0.543 | 0.018 | 0 | 4 | *COL1A2* |
| 0 | 3.858682673 | 0.736 | 0.027 | 0 | 4 | *MYH11* |
| 0 | 1.319984001 | 0.234 | 0.005 | 0 | 4 | *ACTG2* |
| 0 | 2.739049885 | 0.791 | 0.035 | 0 | 4 | *BGN* |
| 0 | 1.533875376 | 0.354 | 0.04 | 0 | 4 | *THY1* |
| 0 | 2.654415163 | 0.72 | 0.015 | 0 | 4 | *PDGFRB* |
| 2.40E-45 | 0.288416267 | 0.254 | 0.149 | 6.68E-41 | 4 | *NRP1* |
| 0 | 0.652668345 | 0.309 | 0.069 | 0 | 4 | *ANGPT2* |
| 0 | 2.001460618 | 0.415 | 0.019 | 0 | 4 | *COL3A1* |
| 0 | 1.499546085 | 0.519 | 0.074 | 0 | 4 | *COL4A1* |
| 0 | 1.752855641 | 0.559 | 0.072 | 0 | 4 | *COL4A2* |
| 0 | 1.982305788 | 0.686 | 0.082 | 0 | 4 | *COL18A1* |
| 9.79E-62 | 1.3349203 | 0.295 | 0.039 | 2.73E-57 | 5 | *KRT17* |
| 9.91E-32 | 1.83479813 | 0.596 | 0.297 | 2.76E-27 | 5 | *KRT19* |
| 6.18E-40 | 1.520660405 | 0.256 | 0.044 | 1.72E-35 | 5 | *KRT7* |
| 2.02E-27 | 1.627769545 | 0.686 | 0.468 | 5.63E-23 | 5 | *CLDN4* |
| 1.59E-14 | 0.938345484 | 0.244 | 0.082 | 4.44E-10 | 5 | *CLDN5* |
| 1.01E-37 | 1.571856638 | 0.244 | 0.041 | 2.82E-33 | 5 | *SELE* |
| 3.25E-08 | 0.830121412 | 0.269 | 0.13 | 0.00090354 | 5 | *VWF* |
| 3.02E-93 | 1.803145381 | 0.795 | 0.176 | 8.40E-89 | 5 | *IFI27* |
| 3.44E-17 | 0.526733936 | 0.776 | 0.413 | 9.58E-13 | 5 | *VIM* |
| **CRPC** | | | | | | |
| 0 | 2.142571806 | 0.96 | 0.602 | 0 | 1 | *KRT8* |
| 1.83E-294 | 2.079010626 | 0.967 | 0.608 | 3.69E-290 | 1 | *KRT18* |
| 1.16E-167 | 1.310217258 | 0.625 | 0.307 | 2.34E-163 | 1 | *KRT19* |
| 1.23E-99 | 1.535750361 | 0.393 | 0.187 | 2.48E-95 | 1 | *AGR2* |
| 1.28E-100 | 0.865060145 | 0.856 | 0.545 | 2.58E-96 | 1 | *CLDN4* |
| 4.80E-127 | 2.431923192 | 0.552 | 0.355 | 9.67E-123 | 1 | *IFI27* |
| 0 | 3.686620714 | 0.867 | 0.039 | 0 | 2 | *CLDN5* |
| 2.52E-71 | 0.925251403 | 0.253 | 0.058 | 5.08E-67 | 2 | *SELE* |
| 0 | 3.442901284 | 0.905 | 0.078 | 0 | 2 | *VWF* |
| 0 | 2.719498766 | 0.896 | 0.127 | 0 | 2 | *ENG* |
| 4.71E-215 | 1.562455808 | 0.985 | 0.456 | 9.49E-211 | 2 | *IGFBP7* |
| 0 | 2.668469827 | 0.994 | 0.352 | 0 | 2 | *IFI27* |
| 0 | 1.992602956 | 0.793 | 0.035 | 0 | 2 | *EMCN* |
| 0 | 0.832847394 | 0.427 | 0.038 | 0 | 2 | *CD200* |
| 2.23E-219 | 2.131770333 | 0.988 | 0.579 | 4.50E-215 | 2 | *VIM* |
| 8.94E-271 | 1.09576053 | 0.415 | 0.047 | 1.80E-266 | 2 | *GJA4* |
| 1.02E-92 | 0.727339352 | 0.612 | 0.212 | 2.06E-88 | 2 | *BGN* |
| 1.12E-266 | 1.383302312 | 0.618 | 0.107 | 2.26E-262 | 2 | *THY1* |
| 0 | 1.556312274 | 0.701 | 0.118 | 0 | 2 | *NRP1* |
| 0 | 3.064246906 | 0.774 | 0.088 | 0 | 2 | *ANGPT2* |
| 0 | 3.198266903 | 0.9 | 0.135 | 0 | 2 | *COL4A1* |
| 0 | 2.978247589 | 0.898 | 0.157 | 0 | 2 | *COL4A2* |
| 1.18E-282 | 1.858713733 | 0.88 | 0.254 | 2.38E-278 | 2 | *COL18A1* |
| 5.61E-15 | 0.448780197 | 0.103 | 0.07 | 1.13E-10 | 3 | *KRT15* |
| 5.13E-15 | 0.710133902 | 0.135 | 0.099 | 1.03E-10 | 3 | *KRT17* |
| 5.23E-27 | 0.352709399 | 0.342 | 0.266 | 1.05E-22 | 3 | *KRT19* |
| 3.80E-16 | 0.399770685 | 0.133 | 0.097 | 7.67E-12 | 3 | *KRT7* |
| 0 | 0.784937943 | 0.631 | 0.35 | 0 | 3 | *CLDN4* |
| 0 | 2.03607078 | 0.659 | 0.033 | 0 | 4 | *CLDN5* |
| 0 | 3.521621448 | 0.706 | 0.039 | 0 | 4 | *SELE* |
| 0 | 3.040336188 | 0.858 | 0.067 | 0 | 4 | *VWF* |
| 0 | 1.889362169 | 0.785 | 0.119 | 0 | 4 | *ENG* |
| 7.44E-264 | 0.919600392 | 0.926 | 0.45 | 1.50E-259 | 4 | *IGFBP7* |
| 0 | 2.223549478 | 0.952 | 0.344 | 0 | 4 | *IFI27* |
| 0 | 1.575010292 | 0.714 | 0.026 | 0 | 4 | *EMCN* |
| 0 | 0.794896684 | 0.42 | 0.032 | 0 | 4 | *CD200* |
| 3.03E-219 | 0.417831611 | 0.233 | 0.028 | 6.12E-215 | 4 | *C7* |
| 1.63E-219 | 1.304167745 | 0.951 | 0.574 | 3.28E-215 | 4 | *VIM* |
| 9.13E-282 | 0.901447958 | 0.37 | 0.059 | 1.84E-277 | 4 | *PTGDS* |
| 8.48E-225 | 0.653609509 | 0.478 | 0.116 | 1.71E-220 | 4 | *NRP1* |
| 9.42E-58 | 0.472673103 | 0.262 | 0.095 | 1.90E-53 | 4 | *ANGPT2* |
| 3.88E-136 | 0.803911351 | 0.448 | 0.139 | 7.82E-132 | 4 | *COL4A1* |
| 4.25E-129 | 0.676643531 | 0.483 | 0.161 | 8.57E-125 | 4 | *COL4A2* |
| 2.40E-40 | 3.945345822 | 0.618 | 0.069 | 4.84E-36 | 5 | *PTGDS* |
| 1.06E-44 | 2.763195858 | 1 | 0.585 | 2.14E-40 | 6 | *VIM* |
| 6.00E-07 | 0.565939821 | 0.149 | 0.038 | 0.012086745 | 6 | *MT1A* |
| 8.37E-13 | 0.85688274 | 0.473 | 0.171 | 1.69E-08 | 6 | *COL4A2* |
| 2.53E-21 | 1.638762133 | 0.662 | 0.265 | 5.09E-17 | 6 | *COL18A1* |
| 0 | 2.548853003 | 0.997 | 0.432 | 0 | 7 | *IGFBP7* |
| 0 | 2.073102396 | 0.99 | 0.56 | 0 | 7 | *VIM* |
| 0 | 2.099925229 | 0.581 | 0.02 | 0 | 7 | *GJA4* |
| 0 | 4.048084055 | 0.796 | 0.087 | 0 | 7 | *RGS5* |
| 0 | 1.448053196 | 0.243 | 0.025 | 0 | 7 | *MT1A* |
| 0 | 0.99118596 | 0.628 | 0.167 | 0 | 7 | *COL1A2* |
| 0 | 3.092412852 | 0.734 | 0.036 | 0 | 7 | *MYH11* |
| 0 | 2.180044434 | 0.407 | 0.02 | 0 | 7 | *ACTG2* |
| 0 | 2.348671647 | 0.908 | 0.175 | 0 | 7 | *BGN* |
| 0 | 2.063336508 | 0.408 | 0.098 | 0 | 7 | *THY1* |
| 0 | 1.868265836 | 0.63 | 0.068 | 0 | 7 | *PDGFRB* |
| 2.79E-52 | 0.401320744 | 0.244 | 0.121 | 5.63E-48 | 7 | *NRP1* |
| 0 | 1.019419908 | 0.444 | 0.079 | 0 | 7 | *ANGPT2* |
| 0 | 1.071127267 | 0.548 | 0.141 | 0 | 7 | *COL3A1* |
| 0 | 1.688998723 | 0.544 | 0.124 | 0 | 7 | *COL4A1* |
| 0 | 1.991310619 | 0.643 | 0.141 | 0 | 7 | *COL4A2* |
| 0 | 2.543245533 | 0.779 | 0.233 | 0 | 7 | *COL18A1* |
| 0 | 0.807184858 | 0.494 | 0.112 | 0 | 8 | *ENG* |
| 0 | 3.390341151 | 0.991 | 0.421 | 0 | 8 | *IGFBP7* |
| 0 | 1.132780254 | 0.208 | 0.021 | 0 | 8 | *C7* |
| 0 | 1.304688045 | 0.987 | 0.553 | 0 | 8 | *VIM* |
| 2.16E-306 | 2.209867968 | 0.27 | 0.052 | 4.34E-302 | 8 | *PTGDS* |
| 6.71E-80 | 0.271926451 | 0.267 | 0.118 | 1.35E-75 | 8 | *RGS5* |
| 0 | 4.092993228 | 0.967 | 0.129 | 0 | 8 | *COL1A2* |
| 1.24E-44 | 0.422787856 | 0.106 | 0.039 | 2.49E-40 | 8 | *ACTG2* |
| 0 | 3.060465668 | 0.897 | 0.163 | 0 | 8 | *BGN* |
| 0 | 1.736227141 | 0.707 | 0.067 | 0 | 8 | *THY1* |
| 0 | 1.087818668 | 0.595 | 0.06 | 0 | 8 | *PDGFRB* |
| 0 | 3.736083459 | 0.891 | 0.105 | 0 | 8 | *COL3A1* |
| 0 | 0.991342217 | 0.567 | 0.114 | 0 | 8 | *COL4A1* |
| 0 | 1.257207822 | 0.657 | 0.13 | 0 | 8 | *COL4A2* |
| 2.01E-220 | 0.535670388 | 0.563 | 0.241 | 4.06E-216 | 8 | *COL18A1* |

Note: A *p*-value of "0" indicates an extremely small value approaching zero.

**Supplementary Table S8.** Reconstructed prostate fine cell marker gene sets

| **Cell type (fine)** | **Marker genes** |
| --- | --- |
| LE cell | *KLK2, KLK3, KLK4, NKX3-1, KRT8,* *KRT18,* *MSMB,* *NPY, TRGC1, PCA3, RDH11, STEAP2,* *PLA2G2A* |
| BE cell | *KRT5, KRT15, KRT14, KRT17,* *TP63, IER3,* *ID1, EGR1, DST, S100A2, S100A6, KRT19* |
| Club cell | *MMP7, PIGR, CP, LCN2,* *RARRES1, KRT7, AGR2* |
| Hillock cell | *KRT19,* *SERPINB1, CLDN4, WFDC2* |
| NE cell | *KRT4, LY6D, ASCL1, CHGB, SYP, FOXA2, ENO2, LMO3, EZH2, SOX2, SIAH2* |
| Endo cell | *CLDN5, SELE, VWF, ENG, IFI27, EMCN, CDH5* |
| Fib | *C1S, C7, VIM, CFD,* *TNFAIP6, APOD, FBLN1, FGF2, PTGDS,* *GJA4, RGS5,* *MT1A,* *IGF1, PDGFRA, FBLN2, COL1A2* |
| MyoFib | *MYH11, GJA4,* *RGS5, MT1A* |
| SMC | *ACTA2, MYH11, ACTG2, TAGLN, BGN, THY1, MYL9,* *TPM2* |
| Pericyte | *THY1, PDGFRB, KCNJ8,* *GUCY1A2,* *NGPT2, COL3A1,* *COL4A1,* *COL4A2, COL18A1,* *COL5A3,* *COL5A2* |

**Supplementary Table S9.** F1-scores of marker genes for fine cell typing

| **Marker** | **Normal_PZ** | **Normal_TZ** | **AN** | **Benign** | **BPH_GN** | **BPH_SN** | **PCa** | **CRPC** |
| --- | --- | --- | --- | --- | --- | --- | --- | --- |
| **LE cell marker genes** | | | | | | | | |
| *KLK2* | 1 | 1 | 1 | 0.8 | 1 | 1 | 0.4 | 1 |
| *KLK3* | 1 | 1 | 1 | 0.8 | 1 | 1 | 0.4 | 1 |
| *KLK4* | 1 | 1 | 1 | 1 | 1 | 1 | 0.667 | 0.667 |
| *NKX3-1* | 1 | 0.667 | 1 | 1 | 0.667 | 0.667 | 0.4 | 1 |
| *KRT8* | NULL | NULL | 0.333 | 0.571 | 0.333 | 0.5 | 0.667 | 0.364 |
| *KRT18* | NULL | NULL | 0.571 | 0.571 | 0.4 | 0.4 | 1 | 0.4 |
| *MSMB* | 1 | 1 | 0.8 | 0.8 | 1 | 0.667 | 0.5 | NULL |
| *NPY* | 1 | 1 | 0.8 | 0.8 | 1 | 1 | 0.571 | NA |
| *TRGC1* | 1 | 0.667 | 0.5 | 1 | 0.5 | 0.5 | 0.667 | 1 |
| *PCA3* | NA | NA | 0.5 | NA | NA | NA | 0.4 | NA |
| *RDH11* | 1 | 1 | 0.8 | 0.8 | 1 | 1 | 0.4 | 0.4 |
| *STEAP2* | 1 | 1 | 1 | 0.8 | 0.667 | 0.667 | 0.4 | 0.667 |
| *PLA2G2A* | 1 | 0.667 | 1 | 0.667 | 0.667 | 1 | 0.333 | 0.333 |
| **BE marker genes** | | | | | | | | |
| *KRT5* | 0.727 | 0.444 | 0.667 | 1 | 0.857 | 0.667 | − | 0.4 |
| *KRT15* | 0.6 | 0.6 | 1 | 1 | 1 | 0.667 | − | 0.8 |
| *KRT14* | 0.6 | 0.25 | 0.667 | 1 | 0.857142857 | 1 | − | 0.5 |
| *KRT17* | 0.6 | 0.6 | 1 | 1 | 0.667 | 0.5 | − | 0.8 |
| *TP63* | 0.25 | NA | 0.667 | 1 | 0.4 | NA | − | 0.5 |
| *IER3* | 0.2 | 0.167 | 0.667 | 0.286 | NULL | NULL | − | 0.222 |
| *ID1* | 0.25 | 0.222 | 0.4 | 0.857 | 0.667 | 0.333 | − | 0.222 |
| *EGR1* | 0.25 | 0.222 | 0.286 | 1 | 0.286 | 0.4 | − | 0.5 |
| *DST* | 0.444 | 0.444 | 0.4 | 1 | 0.857 | 0.667 | − | 0.25 |
| *S100A2* | 0.833 | 0.444 | 0.667 | 0.5 | 0.667 | 0.667 | − | 0.8 |
| *S100A6* | 0.667 | 0.6 | 0.8 | 0.182 | 0.571 | 0.25 | − | 0.545 |
| *KRT19* | 0.6 | 0.727 | 1 | 0.667 | 0.667 | 0.4 | − | 0.75 |
| **Endothelial cell marker genes** | | | | | | | | |
| *CLDN5* | 1 | 1 | 0.8 | 0.667 | 1 | 1 | 0.667 | 1 |
| *SELE* | 1 | 1 | 0.667 | 1 | 0.667 | 1 | 0.667 | 1 |
| *VWF* | 1 | 1 | 1 | 1 | 1 | 1 | 0.667 | 1 |
| *ENG* | 0.667 | 0.5 | 1 | 1 | 1 | 0.667 | 1 | 0.8 |
| *IFI27* | 1 | 1 | 1 | 1 | 1 | 1 | 0.667 | 0.571 |
| *EMCN* | **1** | **1** | **1** | **1** | **1** | **1** | **1** | **1** |
| *CDH5* | 1 | 1 | 1 | 1 | 1 | 0.667 | 1 | 1 |
| **Fib cell marker genes** | | | | | | | | |
| *C1S* | 0.5 | 0.5 | − | − | 0.667 | 0.8 | − | 0.5 |
| *C7* | 0.667 | 0.5 | − | − | 1 | 1 | − | NULL |
| *VIM* | 0.444 | 0.444 | − | − | 0.286 | 0.4 | − | 0.222 |
| *CFD* | 0.667 | 0.5 | − | − | 1 | 1 | − | NULL |
| *TNFAIP6* | 1 | 1 | − | − | 0.5 | 0.5 | − | 0.667 |
| *APOD* | 0.667 | 0.667 | − | − | 1 | 0.8 | − | NULL |
| *FBLN1* | 0.667 | 0.667 | − | − | 1 | 1 | − | NULL |
| *FGF2* | 1 | 1 | − | − | 0.667 | 0.667 | − | 1 |
| *PTGDS* | 0.5 | 0.667 | − | − | 1 | 1 | − | NULL |
| *GJA4* | NULL | NULL | − | − | NULL | NULL | − | NULL |
| *RGS5* | NULL | NULL | − | − | NULL | NULL | − | NULL |
| *MT1A* | 0.5 | 0.5 | − | − | NULL | 0.4 | − | 0.667 |
| *IGF1* | 0.5 | 0.667 | − | − | 1 | 1 | − | NULL |
| *PDGFRA* | 0.667 | 0.667 | − | − | 1 | 1 | − | NULL |
| *FBLN2* | 1 | 1 | − | − | 0.667 | 1 | − | 0.5 |
| *COL1A2* | 0.667 | 0.8 | − | − | 0.5 | 0.667 | − | NULL |
| **SMC cell marker genes** | | | | | | | | |
| *ACTA2* | 0.5 | − | − | − | − | − | − | − |
| *MYH11* | 0.667 | − | − | − | − | − | − | − |
| *ACTG2* | 0.667 | − | − | − | − | − | − | − |
| *TAGLN* | 0.5 | − | − | − | − | − | − | − |
| *BGN* | 0.5 | − | − | − | − | − | − | − |
| *THY1* | NULL | − | − | − | − | − | − | − |
| *MYL9* | 0.5 | − | − | − | − | − | − | − |
| *TPM2* | 0.4 | − | − | − | − | − | − | − |

"NULL": Both precision and recall were 0.

"NA": Markers were not detected as the differential expression genes.

"-": Cell type was not labeled.

Supplementary Figures

**Supplementary Figure S1**

**
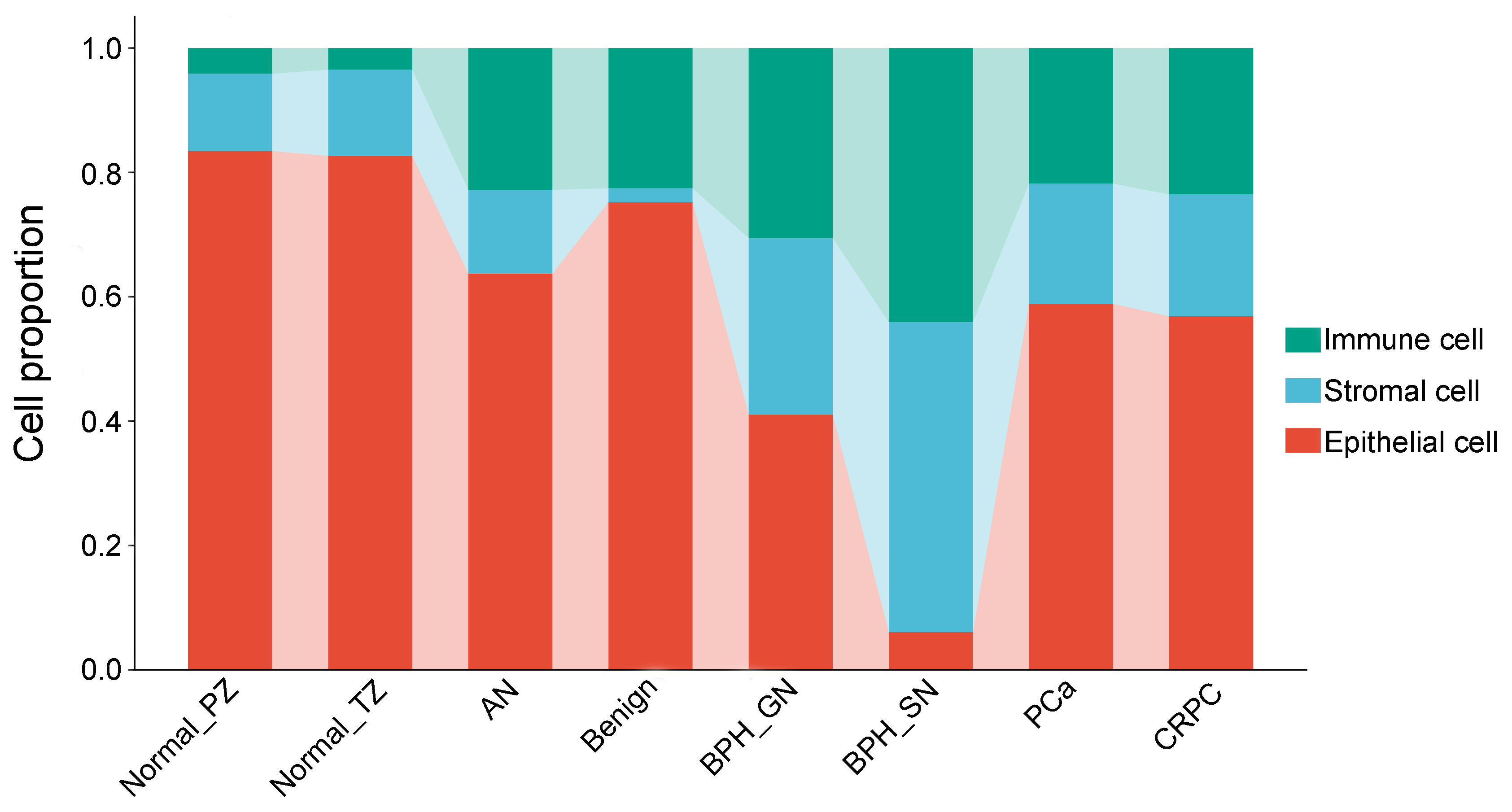
**

**Supplementary Figure S1.** Main cell proportion of the eight integrated human prostate scRNA-seq datasets

**Supplementary Figure S2**


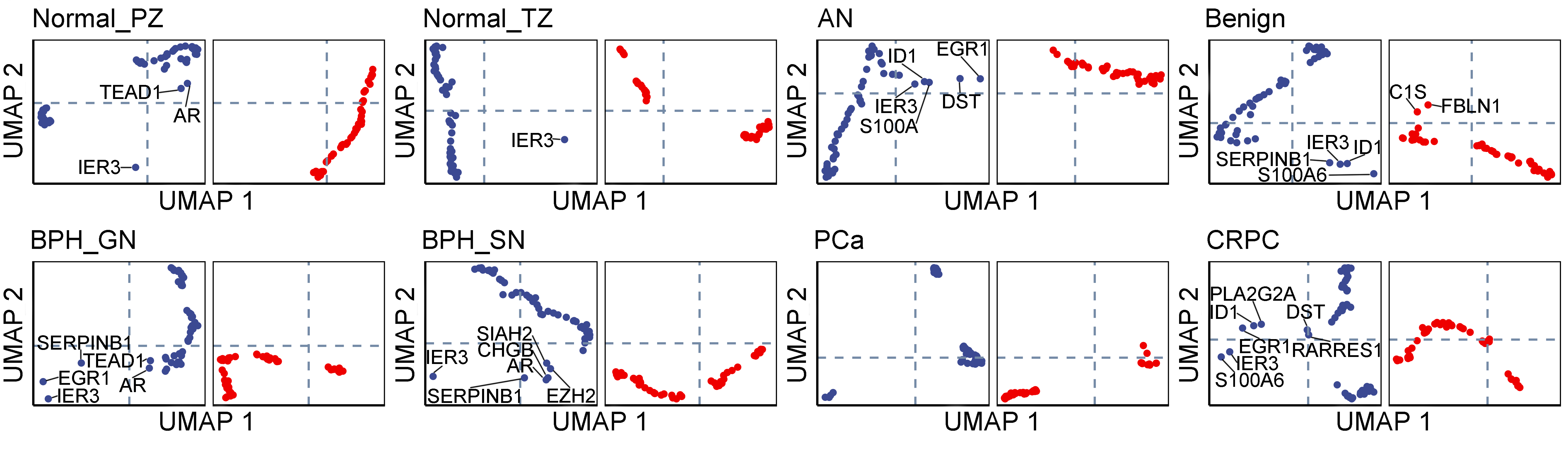


**Supplementary Figure S2.** UMAPs for epithelial and stromal markers of the eight integrated human prostate scRNA-seq datasets

**Supplementary Figure S3**

**
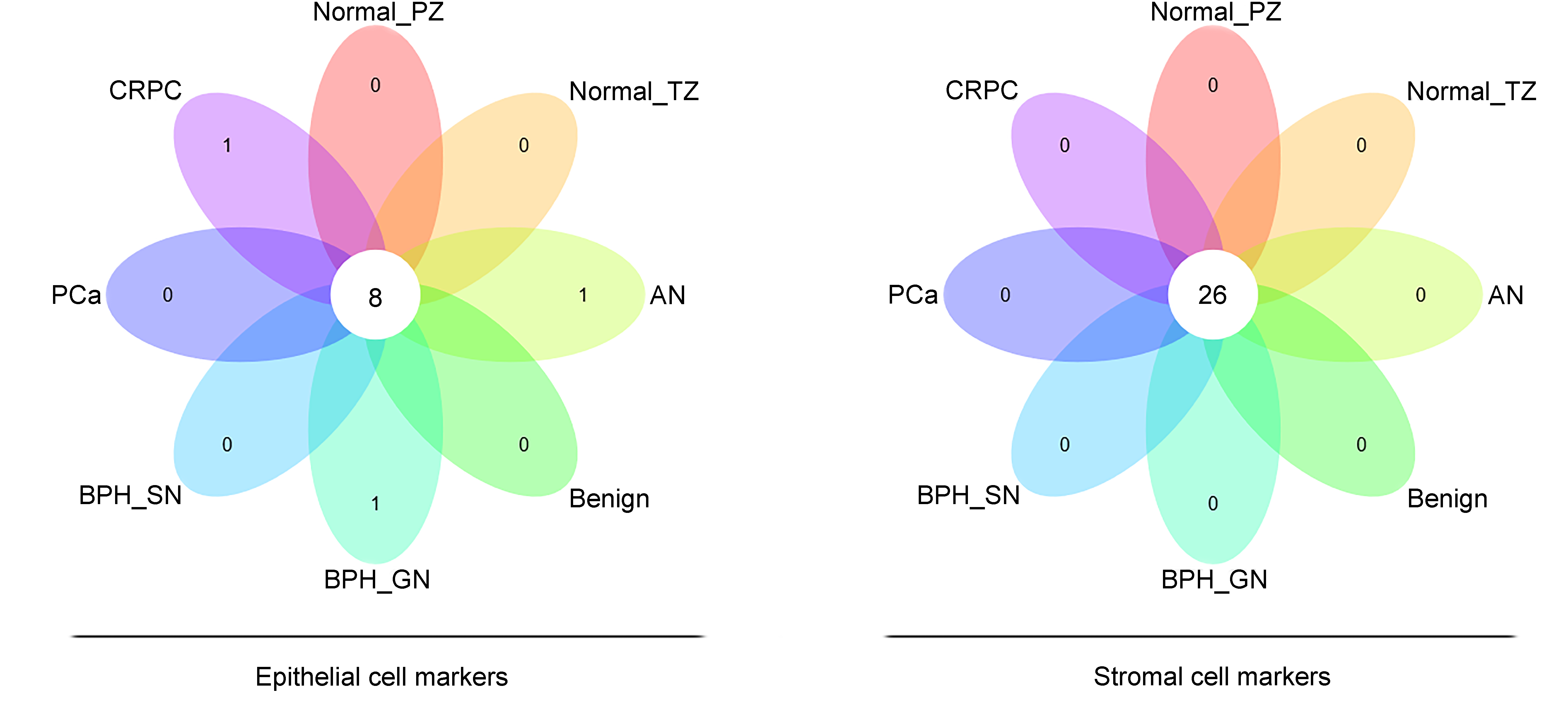
**

**Supplementary Figure S3.** Venn plot of the significantly upregulated epithelial and stromal cell markers

**Supplementary Figure S4**

**
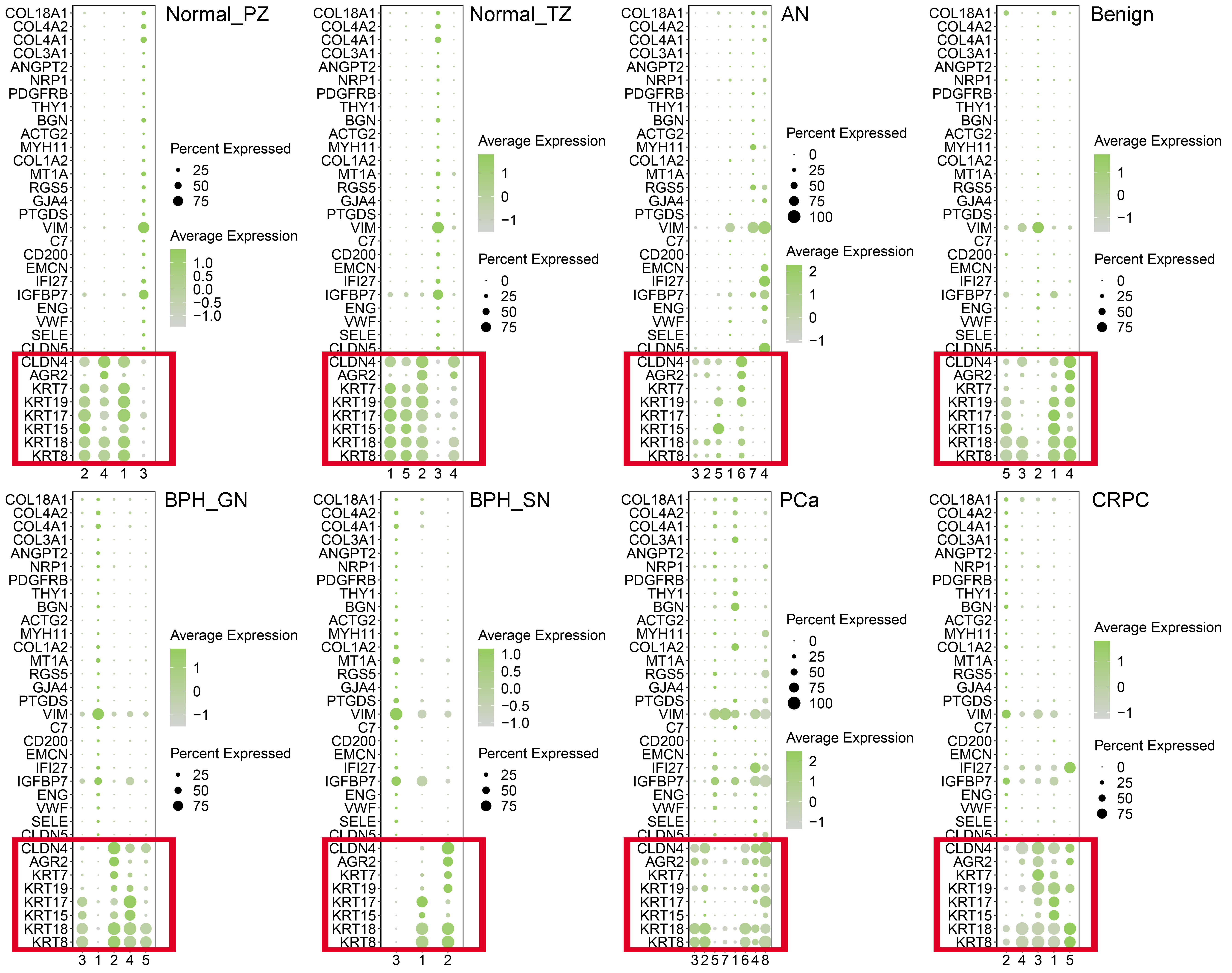
**

**Supplementary Figure S4.** DotPlots of the 34 main cell markers in the KMeans-clusters reclustered according to the average expression levels of the eight epithelial markers

**Supplementary Figure S5**

**
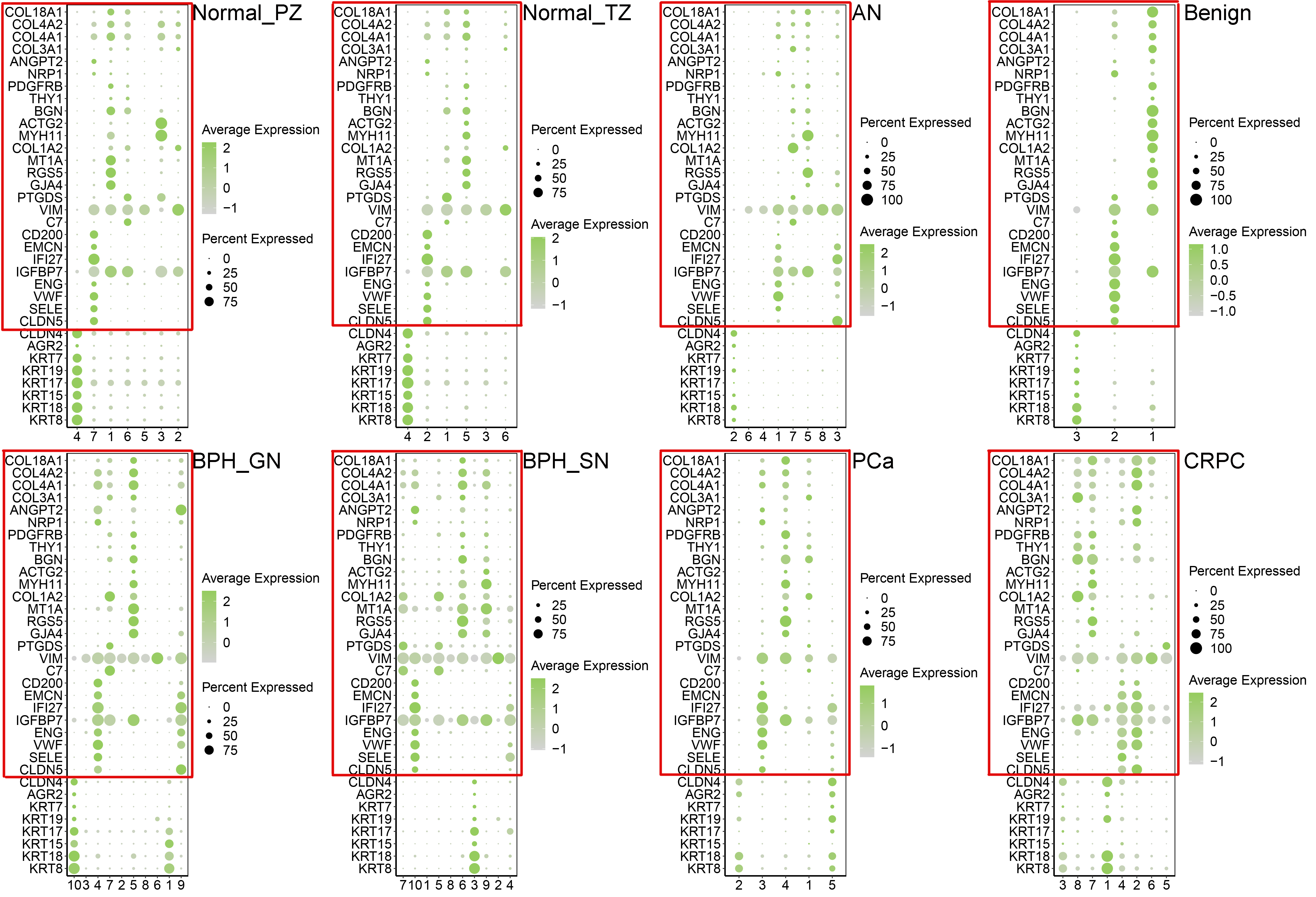
**

**Supplementary Figure S5.** DotPlots of the 34 main cell markers in the KMeans-clusters reclustered according to the average expression levels of the 26 stromal markers

**Supplementary Figure S6**


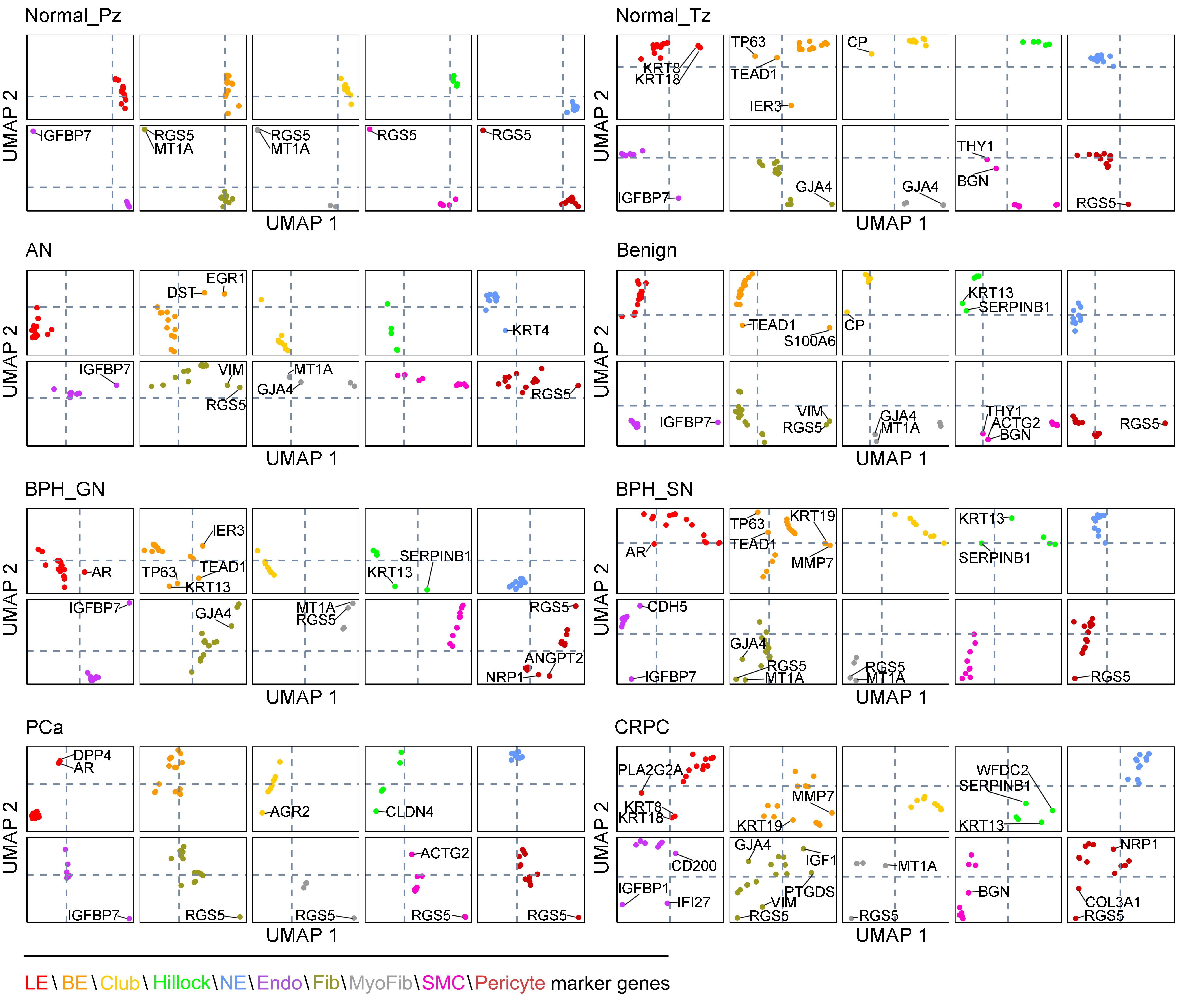


**Supplementary Figure S6.** Evaluating the performance of human prostate fine cell markers using UMAP approach.

Supplementary Data 2

Methods

**Strategy**

Detailed analysis strategy is shown in Figure S7.

**Literature review**

An extensive literature review was conducted to sum prostate cell types and their markers. Accordingly, the inclusion criteria were (i) studies published from January 24, 2018 to January 24, 2023, and (ii) studies performing scRNA-seq with human prostate tissues. In this study, we mainly focused on the ability of a marker to identify a cell type from the whole cell population rather than from certain cell types. Thus, the scRNA-seq studies for a specific cell type (epithelial, stromal, immune, etc.) or human cell lines were excluded. In total, 13 studies were included^1-13^.

**Public human prostate scRNA-seq datasets download.**

37 prostate scRNA-seq data matrices of 41 peoples were downloaded from Gene Expression Omnibus (GEO) public database (https://www.ncbi.nlm.nih.gov/geo/). Based on sample types, they were integrated into seven datasets named Normal, AN, Benign, BPH_GN, BPH_SN, PCa, and CRPC. Notably, considering the predilection of prostate cancer (PCa) for the peripheral zone (PZ) and benign prostatic hyperplasia (BPH) for the transitional zone (TZ), we further integrated normal prostate scRNA-seq data matrices into two datasets, Normal_TZ and Normal_PZ, based on their respective tissue locations. Finally, eight scRNA-seq datasets (Normal_PZ, Normal_TZ, AN, Benign, BPH_GN, BPH_SN, PCa, and CRPC) were gained (Supplementary Table S3 in Supplementary Data 1).

**Public human prostate scRNA-seq datasets processing.**

***Single-cell clustering analysis.*** Matrices were analyzed by using Seurat R package (version 4.3.0)^14^. Firstly, cells with small library size (min.features = 200) and high mitochondrial transcript ratio (percent.mt ≥ 10), and genes expressed in less than three cells (min.cells = 3) were excluded. Then, matrices were further filtered to remove abnormal cells, and a total of 170,438 cells were gained. After treatment with NormalizeData and FindVariableFeatures function, the matrices were integrated into eight datasets and the batch effects were removed using FindIntegrationAnchors and IntegrateData function. Subsequently, the integrated matrices were scaled, and principal component analysis (PCA) was run for linear dimension reduction^1,6^. Subsequently, significant principal components (PCs) were selected and utilized in the FindNeighbors, FindClusters, and RunUMAP functions^1^. Finally, the UMAPs of unsupervised clustering of the eight integrated human prostate scRNA-seq datasets were shown in Figure S8.

***Main cell typing.*** Seurat clusters of each integrated human prostate scRNA-seq dataset were labeled as epithelial, stromal and immune cells (Table S10). This process included three steps (Figure S9). Preliminary cell typing was performed according to the classical immune cell marker *PTPRC* and epithelial marker *EPCAM* (Figure S10). Secondly, epithelial and stromal gene sets (Table S11) were scored to adjust the grouping (Figure S11). Thirdly, DEGA and gene functional analysis were used to further adjust and confirm the grouping. Seurat cluster9 in Benign scRNA-seq dataset was adjusted as epithelial cell, because it highly expressed epithelial markers *KLK4*, *NKX3-1*, *KRT8*, *KRT18*, and *TRGC1*^8-13^ (Figure S12). And Seurat cluster11 in BPH_SN scRNA-seq dataset was adjusted as immune cell, because it highly expressed mast cell markers *TPSB2*, *TPSAB1*, *CPA3*, and *KIT* ^12,13^ (Figure S12). In addition, gene functional analysis showed that the up-regulated genes of Seurat cluster16 in BPH_GN, BPH_SN, and CRPC scRNA-seq datasets were significantly enriched in immune related biological processes (Table S12). Therefore, they were labeled as immune cells. Cell signature gene set was scored by using AddModuleScore function in Seurat R package (version 4.3.0)^1^. Biological process (BP) gene sets of Gene Ontology (GO) was download from Molecular Signatures Database (http://www.gsea-msigdb.org/gsea/), and gene functional analysis was performed by using clusterProfiler R package (version 3.14.3)^15^.

***Fine Cell typing.*** The fine cell markers had lower stability and specificity than the main cell markers. Therefore, they were treated as a whole rather than as individuals for cell typing. Cell signature gene set (Table S11) was scored to identify LE cells, BE cells, Endo cells, Fib and SMCs (Figure S13). The result of cell typing was shown in Table S10.

***DEGA.*** DEGA was performed for each Seurat cluster by using FindAllMarkers function in Seurat R package (version 4.3.0). The parameter “min.pct” was set as “0.10”. The gene with *p*_val_adj<0.05 was regarded as significance.

**Evaluating and validating the performance of prostate cell markers.**

***Evaluating the performance of*** ***prostate cell markers using UMAP and K-Means clustering approach.*** UMAP was performed by using umap function in umap R package (version 0.2.10.0)^16^. K-Means was performed by using kmeans function in factoextra R package (version 1.0.7)^17^, and the parameter “k” was determined by using elbow method.

***Evaluating the performance of*** ***prostate cell markers using entropy evaluation method (EEM).*** The concept of entropy is well suited to measuring the relative strength of comparison criterion to represent the average intrinsic information involved in the decision. This method largely avoids the defects of the subjective assignment method on the weight calculation for each indicator, and a greater value indicates a greater incidence for the assessed indicator within the overall evaluation^18^. Therefore, in this study, we used it to comprehensively evaluate four important parameters in the results of DEGA, including avg_log2FC, pct.1, diff_pct and *p*_value_adj. We provided the rank of cell markers to show their performance intuitively. In addition, we also calculated the total information entropy of markers in the eight integrated human prostate scRNA-seq datasets to identify the robust cell markers.

***Evaluating the performance of prostate cell markers using local outlier factor (LOF) score.*** LOF is an idea of a local anomaly detection algorithm which can overcome the limitation of distance-based outliers detection^19^. The LOF score is the ratio of the local density of a record to that of its k-nearest neighbors. When a point is with LOF score more than one, it is regarded as an abnormal discrete distribution point. And when a point is with LOF score less than one, it is regarded as a normal distribution point. Therefore, we used it to identify the fine cell markers with abnormal discrete distribution in the UMAPs. LOF algorithm was performed using LOF function in DDoutlier R package (version 0.1.0)^20^.

***Validation of the*** ***ability of prostate cell markers.*** To validate the performance of prostate main cell markers, we used K-Means algorithm to regroup the Seurat clusters according to the average gene expression levels of the prostate main cell markers in each Seurat cluster. Then, DEGA was performed to determine the cell type of each KMeans-cluster. The KMeans-cluster significantly highly expressing at least two cell markers was regarded as the corresponding cell type. The KMeans-cluster expressing both epithelial and stromal cell markers equally was regarded as the uncertain cells. Finally, the accuracy, error, precision, recall, and F1-score were calculated. The main cell types of Seurat clusters were regarded as the actual events, and those of KMeans-clusters were treated as the prediction events. For validating the performance of the cell markers to label LE cells, BE cells, Endo cells, Fib, and SMCs, we directly calculated the accuracy, error, precision, recall, and F1-score of each cell marker for the corresponding fine cell typing. The fine cell types of Seurat clusters, determined by the cell signature gene set scores, were regarded as the actual events. The fine cell types of Seurat clusters determined according to whether a cell marker was significantly high expressed were treated as the prediction events.

Four parameters were important in the calculation of accuracy, error, precision, recall and F1-score. They were TP, TN, FP, and FN. TP was the actual true event that was predicted to be positive, and TN was the actual true event that was predicted to be negative. FP was the actual false event that was predicted to be positive, and FN was the actual false event that was predicted to be negative. The calculation formulae are shown below.

|  | $Accuracy=\frac{\left(TP+TN\right)}{(TP+TN+FP+FN)}$ | ⑴ |
| --- | --- | --- |
|  | $Error=1-Accuracy$ | ⑵ |
|  | $Precision=\frac{TP}{(TP+FP)}$ | ⑶ |
|  | $Recall=\frac{TP}{\left(TP+FN\right)}$ | ⑷ |
|  | $F1-score=2*\frac{Precision*Recall}{\left(Precision+Recall\right)}$ | ⑸ |

**Statistical Analysis.**

Statistical analyses were performed using R software (version 4.1.3). Pearson’s correlation analysis was used to determine the correlation between two variables. Statistical significance was set at *p* < 0.05.

**References**

1. Song H, Weinstein HNW, Allegakoen P, Wadsworth MH 2nd, Xie J, Yang H, et al. Single-cell analysis of human primary prostate cancer reveals the heterogeneity of tumor-associated epithelial cell states. Nat Commun. 2022; 13(1): 141.
2. Chen S, Zhu G, Yang Y, Wang F, Xiao YT, Zhang N, et al. Single-cell analysis reveals transcriptomic remodellings in distinct cell types that contribute to human prostate cancer progression. Nat Cell Biol. 2021; 23(1): 87-98.
3. Ma X, Guo J, Liu K, Chen L, Liu D, Dong S, et al. Identification of a distinct luminal subgroup diagnosing and stratifying early stage prostate cancer by tissue-based single-cell RNA sequencing. Mol Cancer. 2020; 19(1): 147.
4. Tuong ZK, Loudon KW, Berry B, Richoz N, Jones J, Tan X, et al. Resolving the immune landscape of human prostate at a single-cell level in health and cancer. Cell Rep. 2021; 37(12): 110132.
5. Henry GH, Malewska A, Joseph DB, Malladi VS, Lee J, Torrealba J, et al. A Cellular Anatomy of the Normal Adult Human Prostate and Prostatic Urethra. Cell Rep. 2018; 25(12): 3530-3542.e5.
6. Chen Y, Zhang P, Liao J, Cheng J, Zhang Q, Li T, et al. Single-cell transcriptomics reveals cell type diversity of human prostate. J Genet Genomics. 2022; 49(11): 1002-1015.
7. Crowley L, Cambuli F, Aparicio L, Shibata M, Robinson BD, Xuan S, et al. A single‐cell Atlas of themouse and human prostate reveals heterogeneity and conservationof epithelial progenitors. eLife 2020; 9: e59465
8. Dong B, Miao J, Wang Y, Luo W, Ji Z, Lai H, et ak. Single-cell analysis supports a luminal-neuroendocrine transdifferentiation in human prostate cancer. Commun Biol. 2020; 3(1): 778.
9. Ge G, Han Y, Zhang J, Li X, Liu X, Gong Y, et al. Single-Cell RNA-seq Reveals a Developmental Hierarchy Super-Imposed Over Subclonal Evolution in the Cellular Ecosystem of Prostate Cancer. Adv Sci (Weinh). 2022; 9(15): e2105530.
10. Yan Q, Wang M, Xia H, Dai C, Diao T, Wang Y, et al. Single-cell RNA-sequencing technology demonstrates the heterogeneity between aged prostate peripheral and transitional zone. Clin Transl Med. 2022; 12(10): e1084.
11. Joseph DB, Henry GH, Malewska A, Reese JC, Mauck RJ, Gahan JC, et al. Single-cell analysis of mouse and human prostate reveals novel fibroblasts with specialized distribution and microenvironment interactions. J Pathol. 2021; 255(2): 141-154.
12. Wong HY, Sheng Q, Hesterberg AB, Croessmann S, Rios BL, Giri K, et al. Single cell analysis of cribriform prostate cancer reveals cell intrinsic and tumor microenvironmental pathways of aggressive disease. Nat Commun. 2022; 13(1): 6036.
13. Heidegger I, Fotakis G, Offermann A, Goveia J, Daum S, Salcher S, et al. Comprehensive characterization of the prostate tumor microenvironment identifies CXCR4/CXCL12 crosstalk as a novel antiangiogenic therapeutic target in prostate cancer. Mol Cancer. 2022; 21(1): 132.
14. Stuart T, Butler A, Hoffman P, Hafemeister C, Papalexi E, Mauck WM, et al. Comprehensive Integration of Single-Cell Data. Cell 2019; 177(7): 1888-1902.e21.
15. Wu T, Hu E, Xu S, Chen M, Guo P, Dai Z, et al. clusterProfiler 4.0: A universal enrichment tool for interpreting omics data. Innovation (Camb) 2021; 2(3): 100141.
16. Tomasz Konopka (2023). umap: Uniform Manifold Approximation and Projection. R package version 0.2.10.0. https://CRAN.R-project.org/package=umap.
17. Alboukadel Kassambara and Fabian Mundt (2020). factoextra: Extract and Visualize the Results of Multivariate Data Analyses. R package version 1.0.7. https://CRAN.R-project.org/package=factoextra.
18. Luo Z, Zhou Z, Hao Y, Feng J, Gong Y, Li Y, et al. Establishment of an indicator framework for the transmission risk of the mountain-type zoonotic visceral leishmaniasis based on the Delphi-entropy weight method. Infect Dis Poverty. 2022; 11(1): 122.
19. Breunig MM, Kriegel HP, Ng RT, Sander J. LOF: Identifying Density-Based Local Outliers. SIGMOD Rec. 2000; 29: 93-104.
20. Jacob H. Madsen (2018). DDoutlier: Distance & Density-Based Outlier Detection. R package version 0.1.0. https://CRAN.R-project.org/package=DDoutlier.

**Figures**


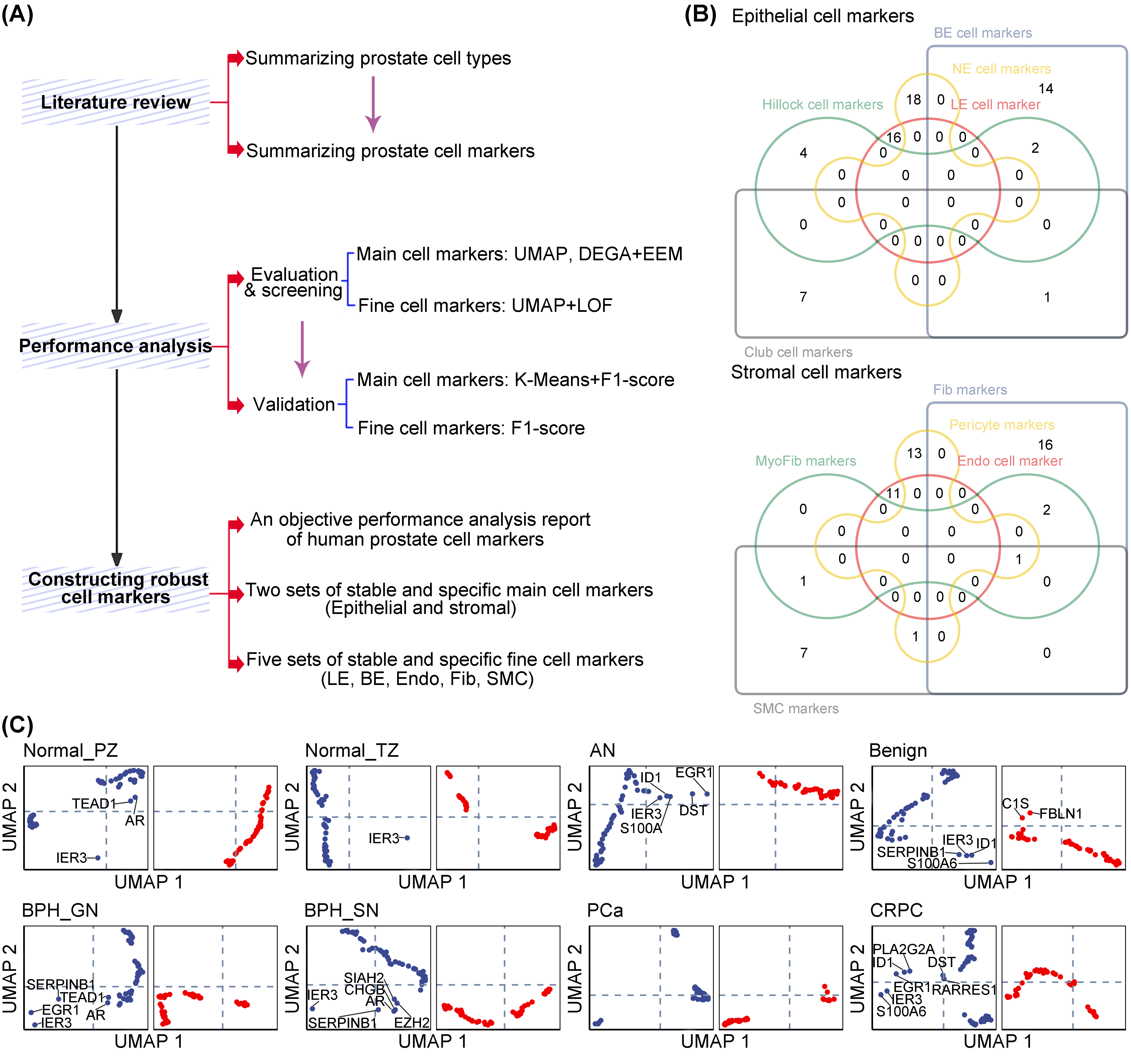


**Figure S7.** Detailed analysis strategy.


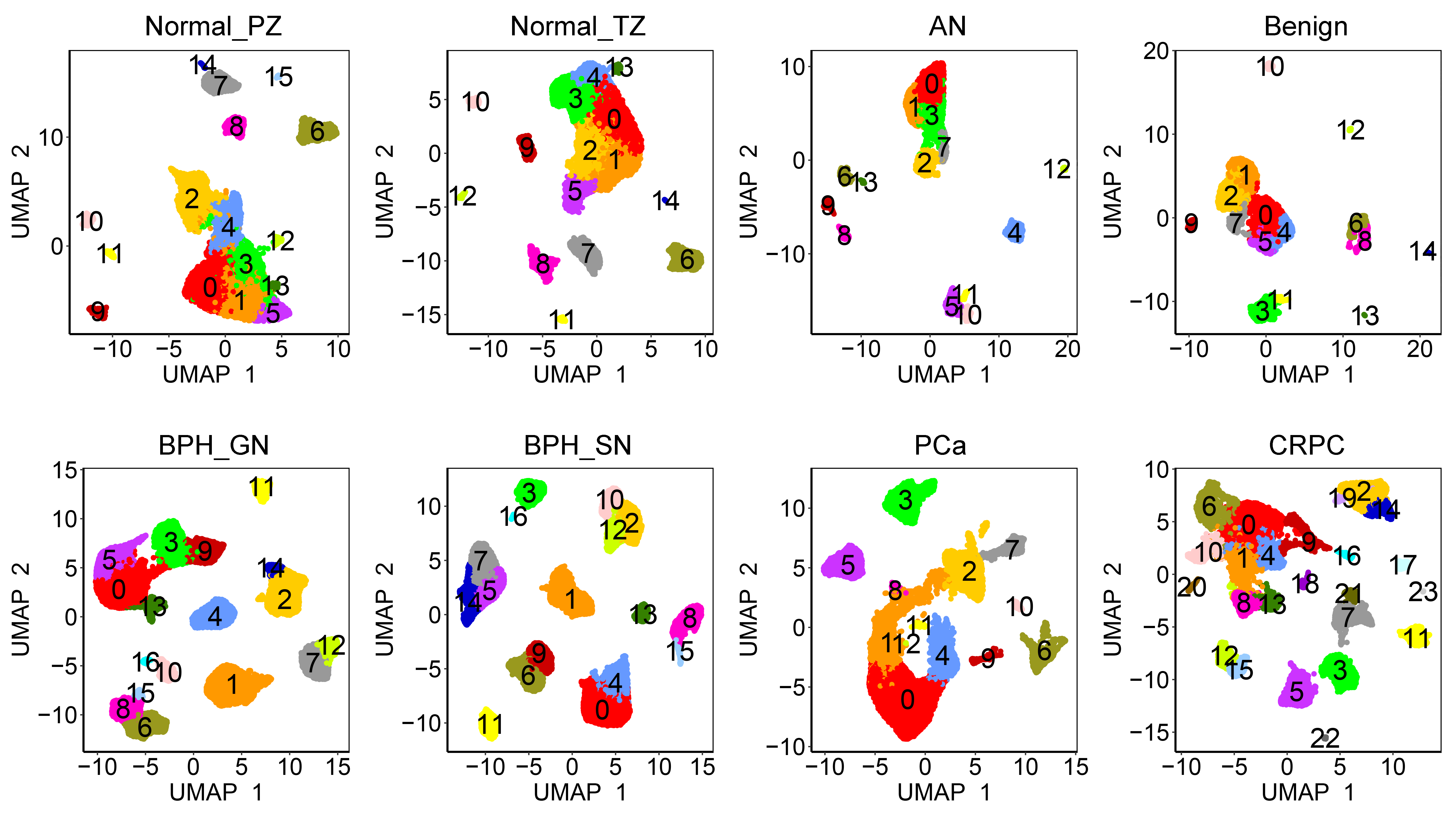


**Figure S8.** UMAPs of unsupervised clustering of the eight integrated human prostate scRNA-seq datasets

**
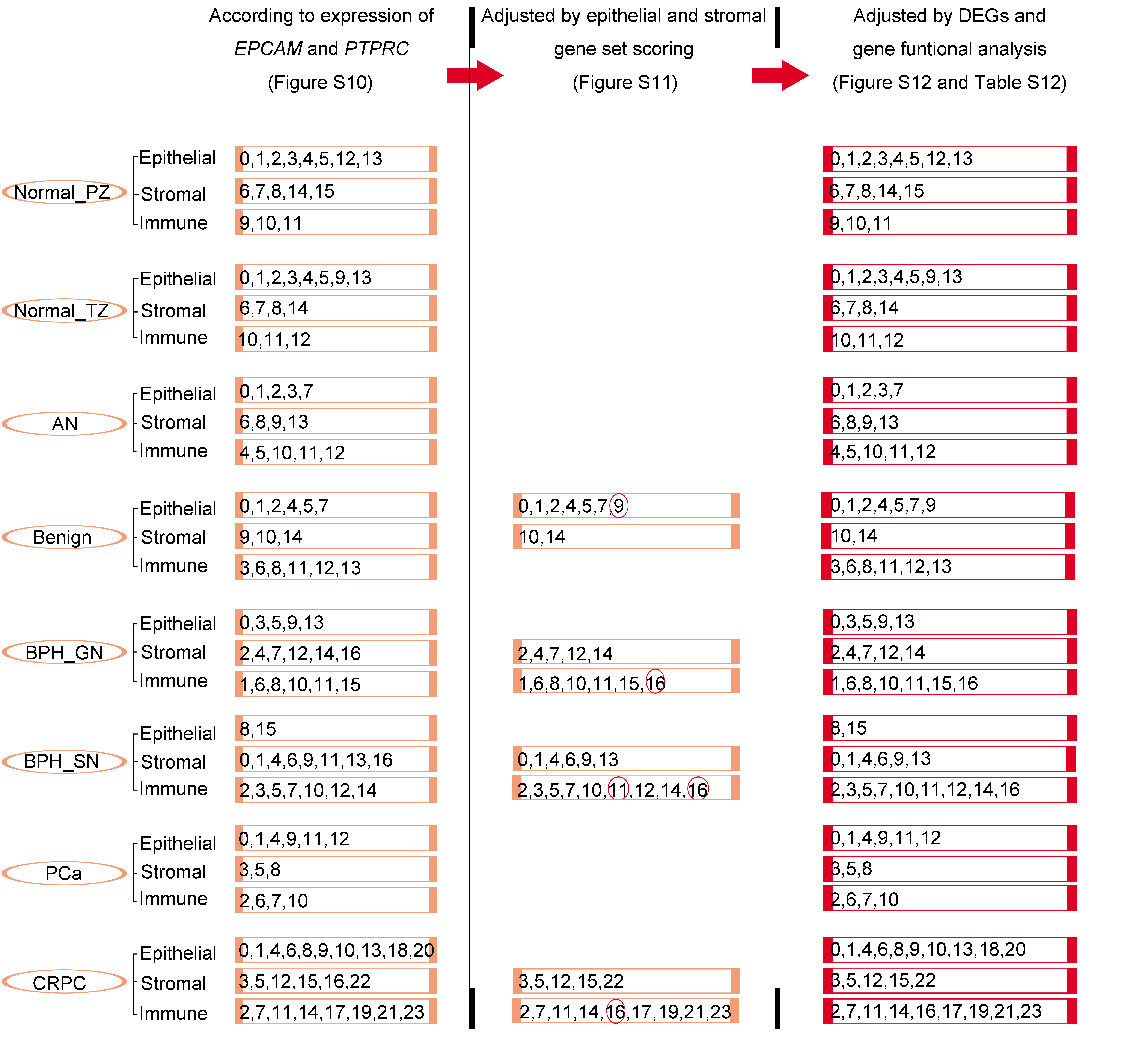
**

**Figure S9.** Process of main cell typing

**
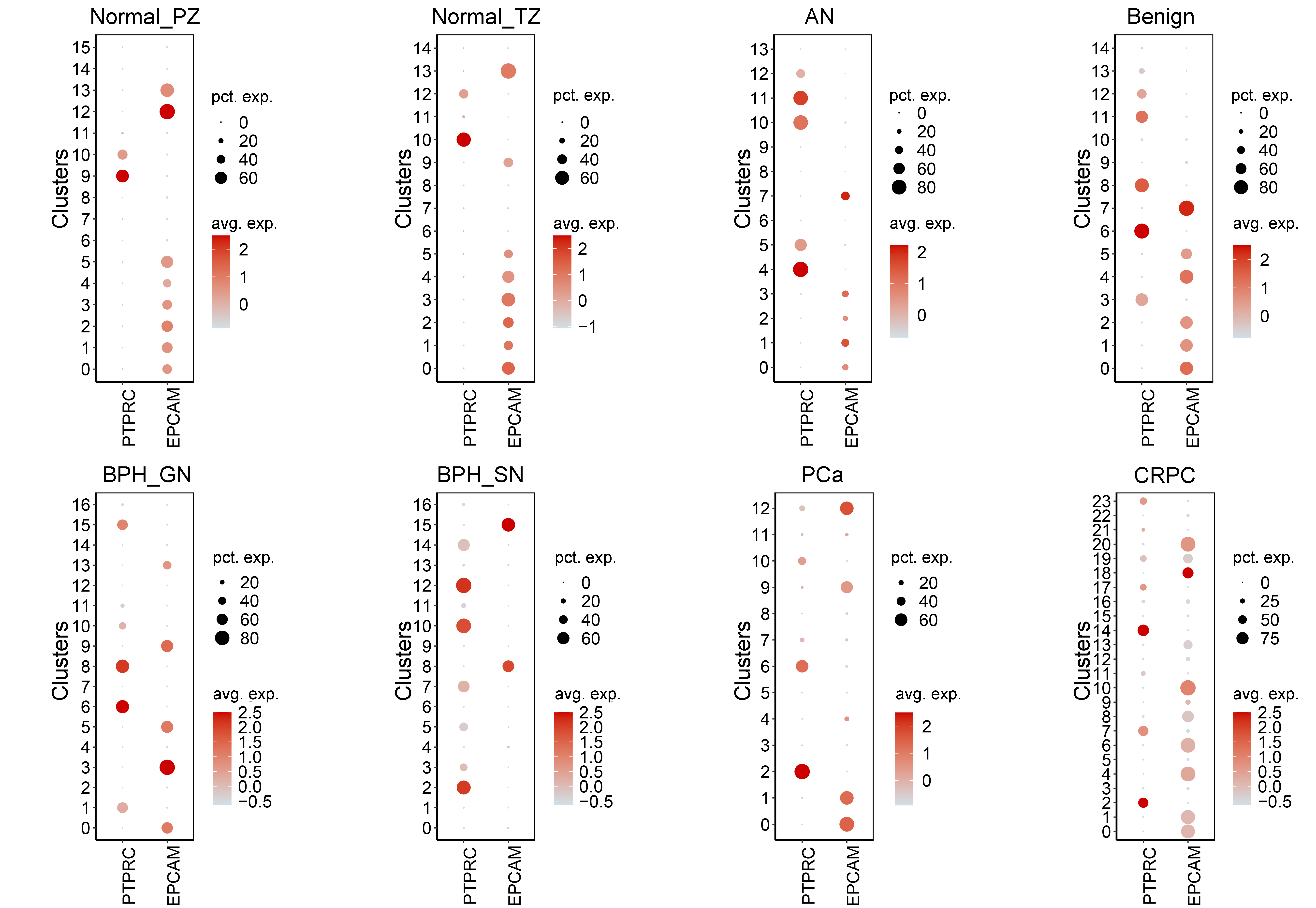
**

**Figure S10.** Expression levels of *PTPRC* and *EPCAM* in each Seurat cluster of the eight integrated human prostate scRNA-seq datasets

**
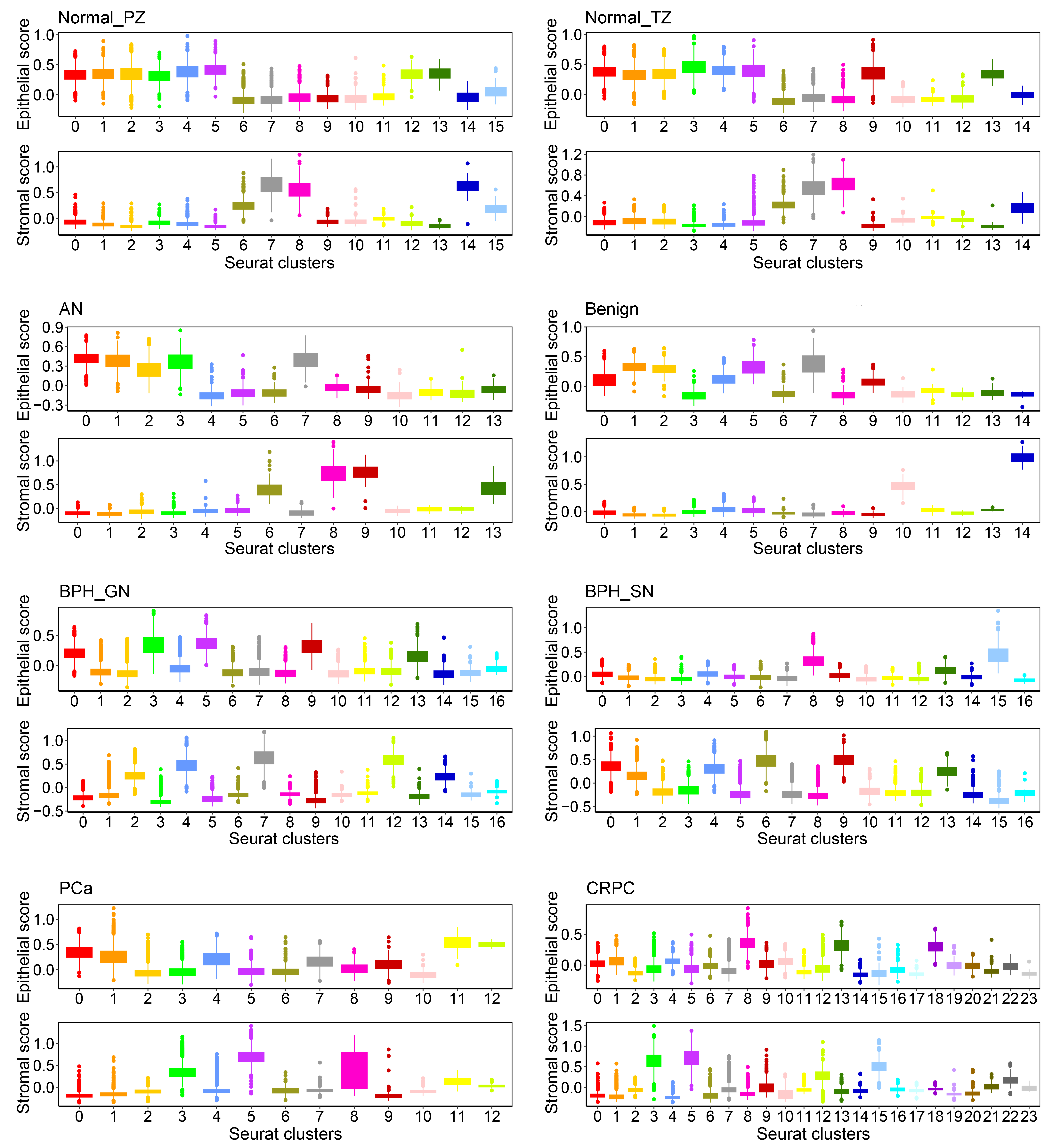
**

**Figure S11.** Epithelial and stromal gene set scores of each Seurat cluster of the eight integrated human prostate scRNA-seq datasets

**
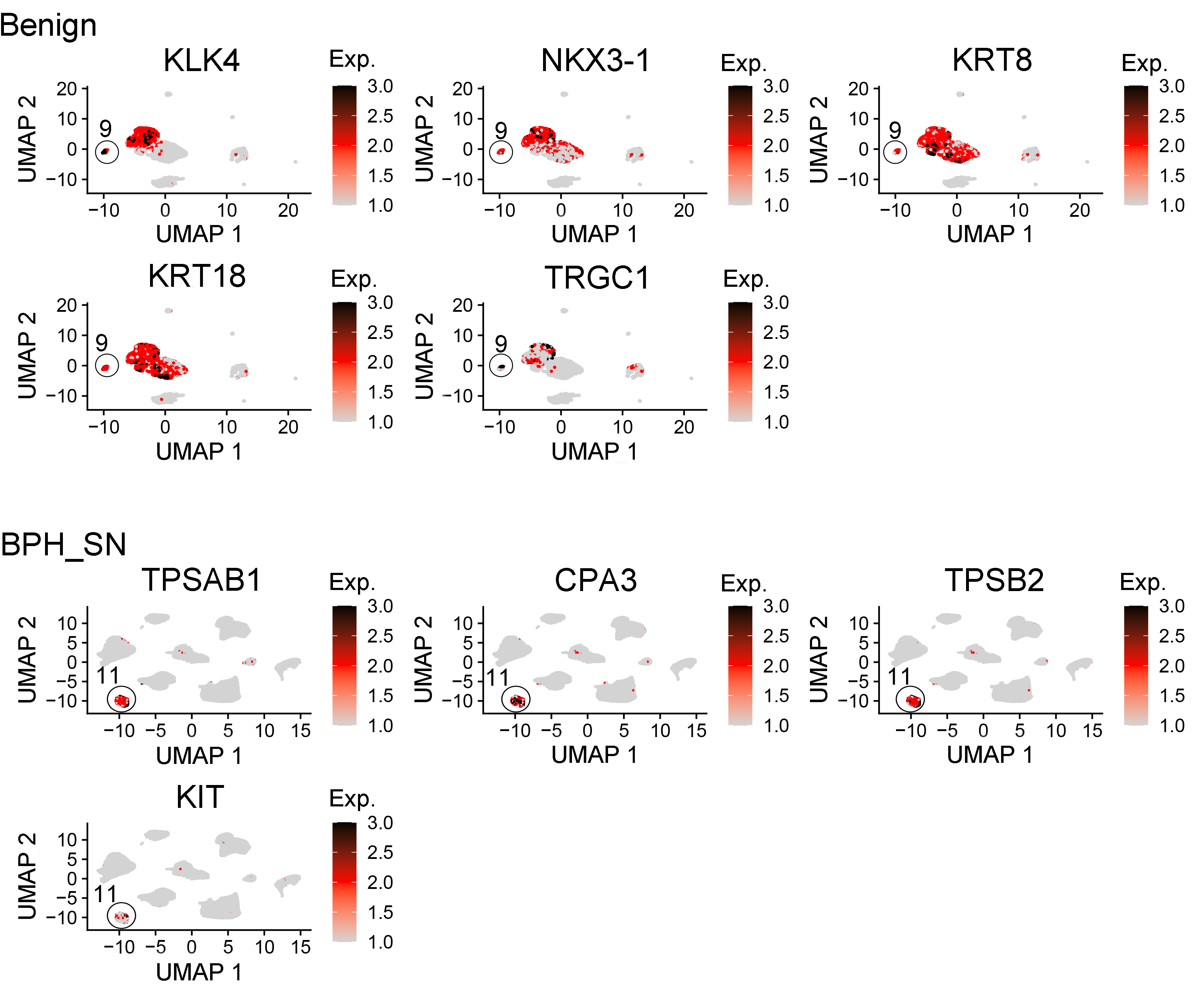
**

**Figure S12.** UMAPs of the differential expression genes in the Benign and BPH_SN datasets. (**Up**) Results of DEGA for Seurat cluster9 of the Benign data set: *KLK4* (avg_log2FC = 2.124, *p*_val_adj < 0.0001); *NKX3-1* (avg_log2FC = 0.941, *p*_val_adj < 0.0001); *KRT8* (avg_log2FC = 0.627, *p*_val_adj = 0.008); *KRT18* (avg_log2FC = 1.185, *p*_val_adj < 0.0001); *TRGC1* (avg_log2FC = 3.123, *p*_val_adj <0.0001). (**Down**) Results of DEGA for Seurat cluster11 of the BPH_SN data set: *TPSAB1* (avg_log2FC = 6.716, *p*_val_adj < 0.0001); *CPA3* (avg_log2FC = 4.304, *p*_val_adj < 0.0001); *TPSB2* (avg_log2FC = 6.824, *p*_val_adj < 0.0001); *KIT* (avg_log2FC = 1.581, *p*_val_adj < 0.0001).


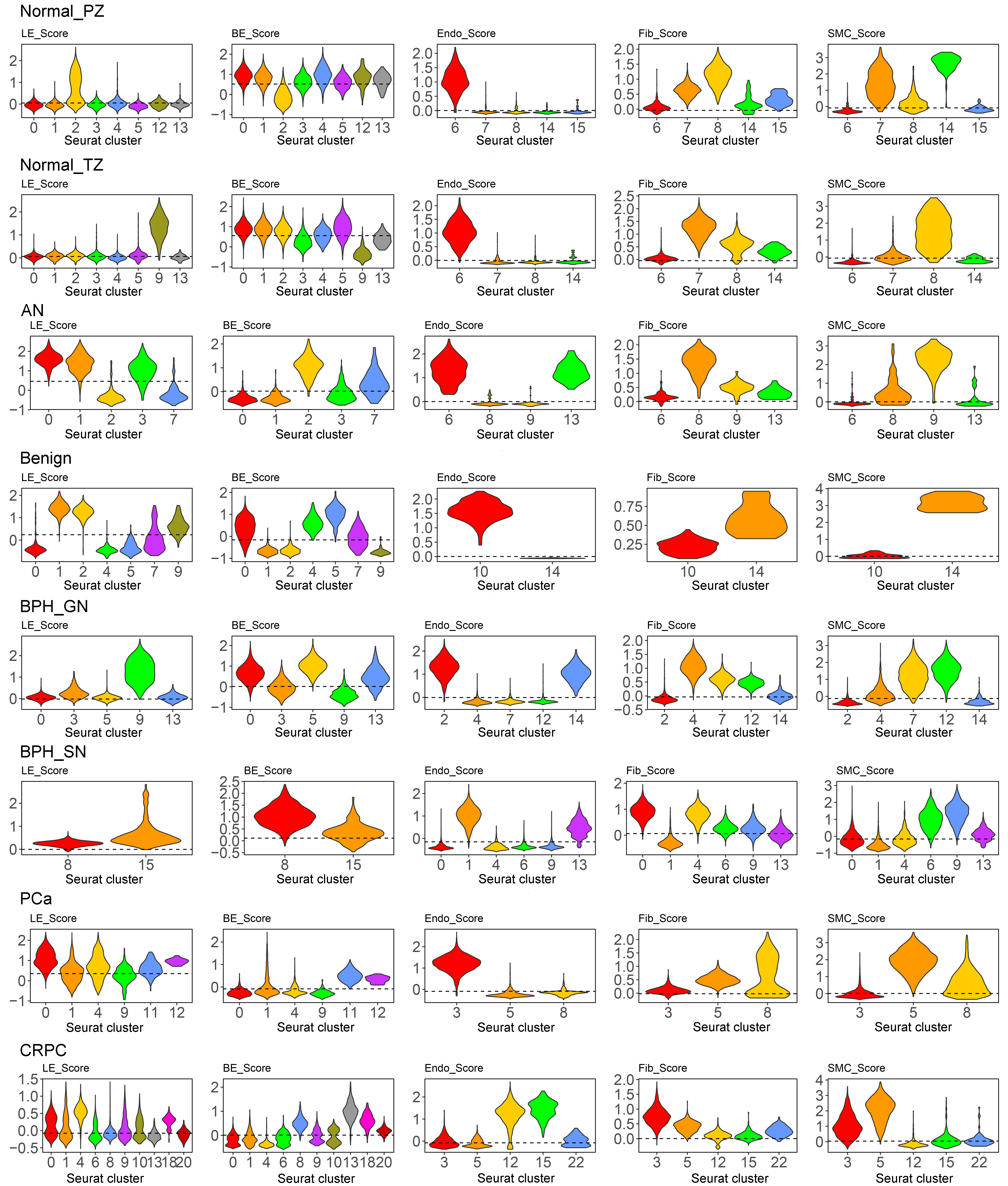


**Figure S13.** LE, BE, Endo, Fib and SMC gene set scores in each Seurat cluster of the eight integrated human prostate scRNA-seq datasets

**Tables**

**Table S10.** Cell types of the eight integrated human prostate scRNA-seq datasets

| **Cell type** | **Normal_PZ** | **Normal_TZ** | **AN** | **Benign** | **BPH_GN** | **BPH_SN** | **PCa** | **CRPC** |
| --- | --- | --- | --- | --- | --- | --- | --- | --- |
| **^1^Main cell types of Seurat clusters** | | | | | | | | |
| Epithelial cell | 0, 1, 2, 3, 4, 5, 12, 13 | 0, 1, 2, 3, 4, 5, 9, 13 | 0, 1, 2, 3, 7 | 0, 1, 2, 4, 5, 7, 9 | 0, 3, 5, 9, 13 | 8, 15 | 0, 1, 4, 9, 11, 12 | 0, 1, 4, 6, 8, 9, 10, 13, 18, 20 |
| Stromal cell | 6, 7, 8, 14, 15 | 6, 7, 8, 14 | 6, 8, 9, 13 | 10, 14 | 2, 4, 7, 12, 14 | 0, 1, 4, 6, 9, 13 | 3, 5, 8 | 3, 5, 12, 15, 22 |
| Immune cell | 9, 10, 11 | 10, 11, 12 | 4, 5, 10, 11, 12 | 3, 6, 8, 11, 12, 13 | 1, 6, 8, 10, 11, 15, 16 | 2, 3, 5, 7, 10, 11, 12, 14, 16 | 2, 6, 7, 10 | 2, 7, 11, 14, 16, 17, 19, 21, 23 |
| **^2^Fine cell types of Seurat clusters** | | | | | | | | |
| LE cell | 2 | 9 | 0, 1, 3 | 1, 2, 9 | 9 | 15 | 0, 1, 4, 9 | 0, 1, 4 |
| BE cell | 0, 1, 3, 4, 5, 12, 13 | 0, 1, 2, 3, 4, 5, 13 | 2, 7 | 0, 4, 5 | 0, 3, 5, 13 | 8 | − | 8, 13, 18 |
| Endo cell | 6 | 6 | 6, 13 | 10 | 2, 14 | 1, 13 | 3 | 12, 15 |
| Fib | 8, 15 | 7, 14 | − | − | 4 | 0, 4 | − | 22 |
| SMC | 14 | − | − | − | − | − | − | − |

^1^ The process of cell typing was shown in Figure 3.

^2^ Cell types were determined based on signature gene set scores.

"-": Cell type was not labeled.

**Table S11.** Gene sets of cell signature

| **Gene sets** | **Markers** |
| --- | --- |
| Epithelial cell | *KLK2, KLK3, KLK4, NKX3-1, AR, KRT8, KRT18, DPP4, MSMB, NPY, TRGC1, PCA3, RDH11, STEAP2, PLA2G2A, KRT5, KRT15, KRT14, KRT17, TP63, MMP7, IER3, ID1, EGR1, DST, S100A2, S100A6, TEAD1, KRT19, KRT13, SCGB3A1, PIGR, CP, LCN2, RARRES1, KRT7, AGR2, SERPINB1, CLDN4, WFDC2, KRT4, LY6D, ASCL1, CHGB, SYP, FOXA2, ENO2, LMO3, EZH2, SOX2, SIAH2* |
| Stromal cell | *CLDN5, SELE, VWF, ENG, IGFBP7, IFI27, EMCN, CDH5, CD200, C1S, C7, VIM, CFD, TNFAIP6, APOD, FBLN1, FGF2, PTGDS, GJA4, RGS5, MT1A, IGF1, PDGFRA, FBLN2, COL1A2, MYH11, ACTA2, ACTG2, TAGLN, BGN, THY1, MYL9, TPM2, PDGFRB, KCNJ8, GUCY1A2, NRP1, ANGPT2, COL3A1, COL4A1, COL4A2, COL18A1, COL5A3, COL5A2* |
| LE cell | *KLK2, KLK3, KLK4, NKX3-1, KRT8, KRT18, MSMB, NPY,TRGC1, PCA3, RDH11, STEAP2, PLA2G2A* |
| BE cell | *KRT15, KRT14, KRT17, TP63, IER3, ID1, EGR1, DST, S100A2, S100A6, KRT19* |
| Endo cell | *CLDN5, SELE, VWF, ENG, IFI27, EMCN, CDH5* |
| Fib | *C1S, C7, VIM, CFD, TNFAIP6, APOD, FBLN1, FGF2, PTGDS, GJA4, RGS5, MT1A, IGF1, PDGFRA, FBLN2, COL1A2* |
| SMC | *ACTA2, MYH11, ACTG2, TAGLN, BGN, THY1, MYL9, TPM2* |

**Table S12.** GO analyses (top ten) of the genes significantly up-regulated in the Seurat cluster16 of the BPH_GN, BPH_SN, and CRPC datasets

| **Description** | ***P* value** | ***P* .adjust** | **Count** |
| --- | --- | --- | --- |
| **Seurat cluster16 of the BPH_GN dataset** | | | |
| B_CELL_RECEPTOR_SIGNALING_PATHWAY | 6.59E-20 | 1.05E-16 | 18 |
| ADAPTIVE_IMMUNE_RESPONSE | 9.41E-17 | 7.49E-14 | 29 |
| ACTIVATION_OF_IMMUNE_RESPONSE | 4.65E-16 | 1.87E-13 | 26 |
| IMMUNE_RESPONSE_REGULATING_SIGNALING_PATHWAY | 4.71E-16 | 1.87E-13 | 25 |
| PHAGOCYTOSIS | 8.00E-16 | 2.55E-13 | 22 |
| IMMUNE_EFFECTOR_PROCESS | 3.49E-15 | 9.28E-13 | 36 |
| ANTIGEN_RECEPTOR_MEDIATED_SIGNALING_PATHWAY | 4.72E-15 | 1.07E-12 | 20 |
| B_CELL_ACTIVATION | 5.65E-15 | 1.13E-12 | 20 |
| POSITIVE_REGULATION_OF_B_CELL_ACTIVATION | 9.65E-15 | 1.71E-12 | 15 |
| HUMORAL_IMMUNE_RESPONSE_MEDIATED_BY_CIRCULATING_IMMUNOGLOBULIN | 1.79E-14 | 2.85E-12 | 15 |
| **Seurat cluster16 of the BPH_SN dataset** | | | |
| DEFENSE_RESPONSE | 1.0845E-14 | 3.75E-11 | 74 |
| CELL_ACTIVATION | 8.18E-14 | 1.41E-10 | 64 |
| IMMUNE_EFFECTOR_PROCESS | 2.17E-12 | 2.50E-09 | 57 |
| REGULATION_OF_IMMUNE_SYSTEM_PROCESS | 3.57E-11 | 3.09E-08 | 62 |
| CYTOKINE_PRODUCTION | 1.05E-10 | 7.25E-08 | 41 |
| POSITIVE_REGULATION_OF_IMMUNE_SYSTEM_PROCESS | 1.44E-10 | 8.32E-08 | 47 |
| IMMUNE_RESPONSE_REGULATING_SIGNALING_PATHWAY | 2.13E-10 | 1.05E-07 | 31 |
| LEUKOCYTE_MEDIATED_IMMUNITY | 6.18E-10 | 2.52E-07 | 41 |
| CYTOKINE_MEDIATED_SIGNALING_PATHWAY | 6.56E-10 | 2.52E-07 | 39 |
| REGULATION_OF_IMMUNE_RESPONSE | 8.35E-10 | 2.89E-07 | 44 |
| **Seurat cluster16 of the CRPC dataset** | | | |
| POSITIVE_REGULATION_OF_B_CELL_ACTIVATION | 5.58E-19 | 1.04E-15 | 19 |
| B_CELL_RECEPTOR_SIGNALING_PATHWAY | 1.51E-18 | 1.04E-15 | 18 |
| HUMORAL_IMMUNE_RESPONSE | 1.66E-18 | 1.04E-15 | 26 |
| REGULATION_OF_B_CELL_ACTIVATION | 4.51E-18 | 2.11E-15 | 20 |
| B_CELL_ACTIVATION | 6.92E-18 | 2.59E-15 | 24 |
| HUMORAL_IMMUNE_RESPONSE_MEDIATED_BY_CIRCULATING_IMMUNOGLOBULIN | 2.85E-17 | 8.89E-15 | 18 |
| LYMPHOCYTE_ACTIVATION | 1.73E-16 | 4.64E-14 | 32 |
| COMPLEMENT_ACTIVATION | 3.43E-16 | 8.05E-14 | 18 |
| POSITIVE_REGULATION_OF_IMMUNE_SYSTEM_PROCESS | 1.82E-15 | 3.43E-13 | 36 |
| B_CELL_MEDIATED_IMMUNITY | 1.83E-15 | 3.43E-13 | 19 |
